# Advanced High Energy Density Secondary Batteries with Multi‐Electron Reaction Materials

**DOI:** 10.1002/advs.201600051

**Published:** 2016-05-17

**Authors:** Renjie Chen, Rui Luo, Yongxin Huang, Feng Wu, Li Li

**Affiliations:** ^1^ Beijing Key Laboratory of Environmental Science and EngineeringSchool of Material Science & EngineeringBeijing Institute of TechnologyBeijing100081P. R. China; ^2^ Collaborative Innovation Center of Electric Vehicles in BeijingBeijing100081P. R. China

**Keywords:** high energy density, multi‐elelctron reaction, secondary battery

## Abstract

Secondary batteries have become important for smart grid and electric vehicle applications, and massive effort has been dedicated to optimizing the current generation and improving their energy density. Multi‐electron chemistry has paved a new path for the breaking of the barriers that exist in traditional battery research and applications, and provided new ideas for developing new battery systems that meet energy density requirements. An in‐depth understanding of multi‐electron chemistries in terms of the charge transfer mechanisms occuring during their electrochemical processes is necessary and urgent for the modification of secondary battery materials and development of secondary battery systems. In this Review, multi‐electron chemistry for high energy density electrode materials and the corresponding secondary battery systems are discussed. Specifically, four battery systems based on multi‐electron reactions are classified in this review: lithium‐ and sodium‐ion batteries based on monovalent cations; rechargeable batteries based on the insertion of polyvalent cations beyond those of alkali metals; metal–air batteries, and Li–S batteries. It is noted that challenges still exist in the development of multi‐electron chemistries that must be overcome to meet the energy density requirements of different battery systems, and much effort has more effort to be devoted to this.

## 1 Introduction

In response to considerations on decreasing the dependence on fossil fuels and related carbon emissions and developing alternative energy sources, the development of high‐efficiency, environmentally friendly, low‐cost, and reliable energy storage systems has become a necessity.^1^ Electrical energy storage (EES) offers a well‐established approach to possibly addressing this matter, providing electricity generated from wind, solar, or nuclear energy, which is essential for sustainable future energy supply.^2^ Technological solutions based on electrochemical energy storage principles have driven dramatic expansion in fields ranging from portable electronics to heavy electric vehicles, and even intermittent transmission and distribution electric grid support, because of their desirable advantages of being pollution‐free, versatile, long lived, and low maintenance.^3^ Thus, the challenge as it now stands is what materials can be used on a large scale with high energy density while still maintaining costs low enough for wide market penetration, rather than how to convert and store energy.^4^ Therefore, electrochemical storage technology needs urgent development to meet the requirements of practical energy storage. Electrochemical batteries represent an excellent class of energy storage technology owing to their high efficiency, flexibility power and energy characteristics, modularity and scalability, which store energy through charge transfer reactions.[[qv: 1a,2]]

Currently available secondary batteries mainly include alkali rechargeable batteries based on Ni‐cathodes (Ni–Cd, Ni–Zn, and Ni–metal‐hydride (Ni–MH) batteries), electric double layer capacitors, and lithium‐ion batteries.^5^ Ni‐cathode batteries have a typical capacity of ≈1.5 kWh, designed to be cathode‐limited even though Ni‐cathodes possess the intrinsic characteristics of two‐electron transfer.^6^ Historically, the rate of increase in energy density seen during the development of various secondary batteries from the start of the 20th century was steady until the appearance of Li‐ion batteries.^7^

Rechargeable lithium‐ion batteries (LIBs) have been comprehensively studied and successfully employed as power sources, with advantages such as high operating voltage, high rate capability, and long cycle life.[[qv: 3b,8]] Li‐ion batteries have already become the choice energy storage system for hybrid electric vehicles, mobile electronic devices, and off‐peak energy storage because of their recognized potential for the realization of sustainable energy development.[[qv: 8a,9]] The wide range of applications of LIBs has initiated intensive research into electrode materials that meet the important criteria of high energy density, safety, and cost reduction.^10^ However, an opinion has been emerging that the state‐of‐the art Li‐ion batteries are far behind the targets for mass market adoption of EVs and large‐scale stationary EES, even with the assumption that the theoretical capacity of the electrode materials will be achieved.^7^, ^11^ Moreover, the expansion of EV and grid applications raises issues related to the uneven global distribution and increasing cost of lithium resources. As a result, significant research efforts have been focused on fundamental investigations into different battery chemistries, known as “beyond Li‐ion”.

Beyond Li‐ion chemistries involve (1) batteries consisting of intercalation‐compounds based on monovalent charge carriers Na and K, and polyvalent Mg, and Al cations, as well as (2) batteries based on conversion reactions such as metal–air batteries and Li–S batteries, in which anions also take part.^2^, ^7^, ^12^ World‐wide research on Na‐ion batteries has increased dramatically owing to the abundance of sodium resources, their environmental and cost friendliness, and similar chemical properties to those of Li‐ion batteries.^13^ A number of cathode and anode materials with attractive properties have been explored during the past few years, even though sodium has a larger ionic radius and heavier atomic mass than lithium, thus leading to smaller theoretical energy density.^14^ As for secondary polyvalent batteries based on Mg and Al, the challenges to confront include finding suitable magnesium insertion compounds and exploring suitable electrolytes for reversible cycling.^15^ Apart from batteries based on intercalation chemistries, metal–air batteries and Li–S batteries involving conversion chemistries have attracted much attention as suitable electrochemical energy storage solutions. Metal‐air batteries and Li–S batteries have been expected to obtain energy densities 2–10 times greater than those of the current generation of Li‐ion batteries, but still have a long way to go in both fundamental research and application.^16^ No currently developed technology can store energy repeatedly and efficiently at low cost; therefore, developing cathode and anode materials and new battery systems with high energy density is an urgent requirement. Theoretical thermodynamic calculations of the energy densities of possible batteries and related materials have been performed to determine the theoretical energy storage limit of many possible power sources, and select systems with high energy densities for development as next generation batteries.^7^

Theoretically, the multi‐electron concept represents a novel horizon for improving battery energy densities. Understanding of the multi‐electron mechanisms of electrochemical processes is crucial to guide the design of advanced electrode materials and their corresponding application in secondary batteries. In this review, we discuss the multi‐electron redox reactions that occur during the electrochemical processes of different battery technologies. Although there are many excellent reviews regarding the electrochemistry of different battery chemistries, our aim is to explore new battery systems and novel electrode materials from the perspective of multi‐electron reactions. We discuss the theoretical foundation of multi‐electron reactions in Section 2, the fundamental reversible reaction mechanisms assigned to multi‐electron transfer in Li‐ion batteries and Na‐ion batteries in Section 3, multi‐electron reactions occurring in secondary polyvalent batteries in Section 4, multi‐electron reactions in metal–air batteries in section 5, Li–S batteries in Section 6, and provide a summary and outlook in Section 7.

## 2 Theoretical Foundation of Multi‐Electron Reactions

For a given chemical reaction (1), electrochemical energy storage occurs when charge transfers.^7^

(1) αA+βB→γC+δD

The Gibbs free energy of the reaction under standard conditions $\Delta_{r}G^{\Theta }$ can be calculated according to the Nernst equation:

(2) $$\Delta_{r}G^{\Theta }=-nEF$$

Here, *n* refers to the number of the charge transferred during the reaction per mole of reactant, *E* is the thermodynamic equilibrium voltage, and *F* is the Faraday constant. Energy density can always be expressed by the gravimetric energy density Wh kg^−1^ or the volumetric energy density Wh L^−1^. Thus, the gravimetric energy density $\varepsilon_{M}$ and volumetric energy density $\varepsilon_{V}$ of a battery can be calculated using Equation (3) and Equation (4), respectively:

(3) $$\varepsilon_{M}=\Delta_{r}G^{\Theta }/\overset{M}{\sum }$$

(4) $$\varepsilon_{V}=\Delta_{r}G^{\Theta }/\overset{V_{M}}{\sum }$$

Where $\sum^{M}$ represents the sum of the formula mole weights of the two reactants and $\sum^{V_{M}}$ is the sum of the formula mole volume of the two reactants.

The specific capacity of a given electrode material can be calculated from Equation (5) (*M* refers to the mole weight of the electrode material):

(5) $$Q=nF/3.6M\;\;\left(\mathrm{mAh}\;g^{-1}\right)$$

Using Equations (1), (2), (3), (4), the theoretical energy density can be calculated when the values of the Gibbs formation energy of the electrode material is known. And if the Gibbs formation energy of the reactant is not known, it can be obtained through first principles calculations.^17^

According to Equations (1), (2), (3), (4), (5), energy density can be improved by i) using electrode materials with high specific capacity, ii) using cathode materials with high redox potential, iii) using anode materials with low redox potential, iv) using active materials that transfer more electrons per molecule.[[qv: 8a,13a]] However, any corresponding increase in battery voltage may lead to irreversible side reactions in terms of electrolyte decomposition and unfavorable safety problems. Present commercial electrolytes under development are only stable up to 5 V. The theoretical lithium storage capacity of a material is determined by the number of transferable charges and amount of Li ion or Na ion uptake. It is easy to estimate the number of exchanged electrons. Obviously, developing multi‐electron electrode materials with smaller mole weight may be an effective approach to further increase the energy density, and the concept was first put forward by Xue‐Ping Gao and Han‐Xi Yang in 2010.^6^ The possibility of multi‐electron reaction is determined by the characteristics of active materials with a variety of chemical valences in the accessible potential window.

**Figure 1:**
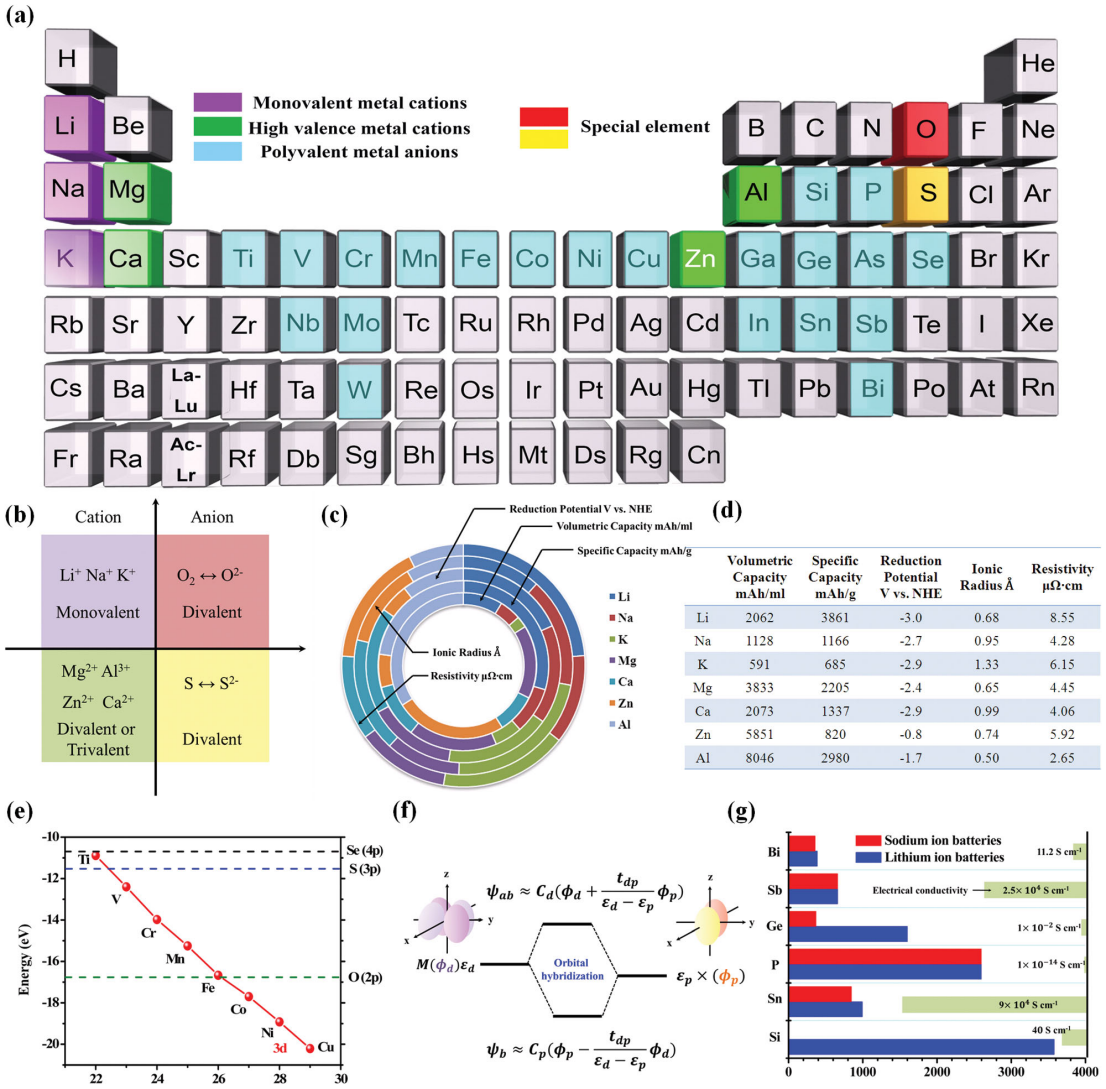
a) Periodic table of elements investigated for multi‐electron reaction. Purple exhibits alkali metal, green is cations with polyvalent, light blue is transition metals with variable valence. b) Four parts involved in multi‐electron reactions in this review. c,d) Comparison of physical properties for alkali metal such as Li, Na and K, and multivalent cations such as Mg, Ca, Zn, and Al as charge carrier for rechargeable batteries. e,f The binding energy of transition metals. g) Comparison of different specific capacity of elements involved in alloying reactions in Li–ion batteries and Na–ion batteries.

**Figure 2:**
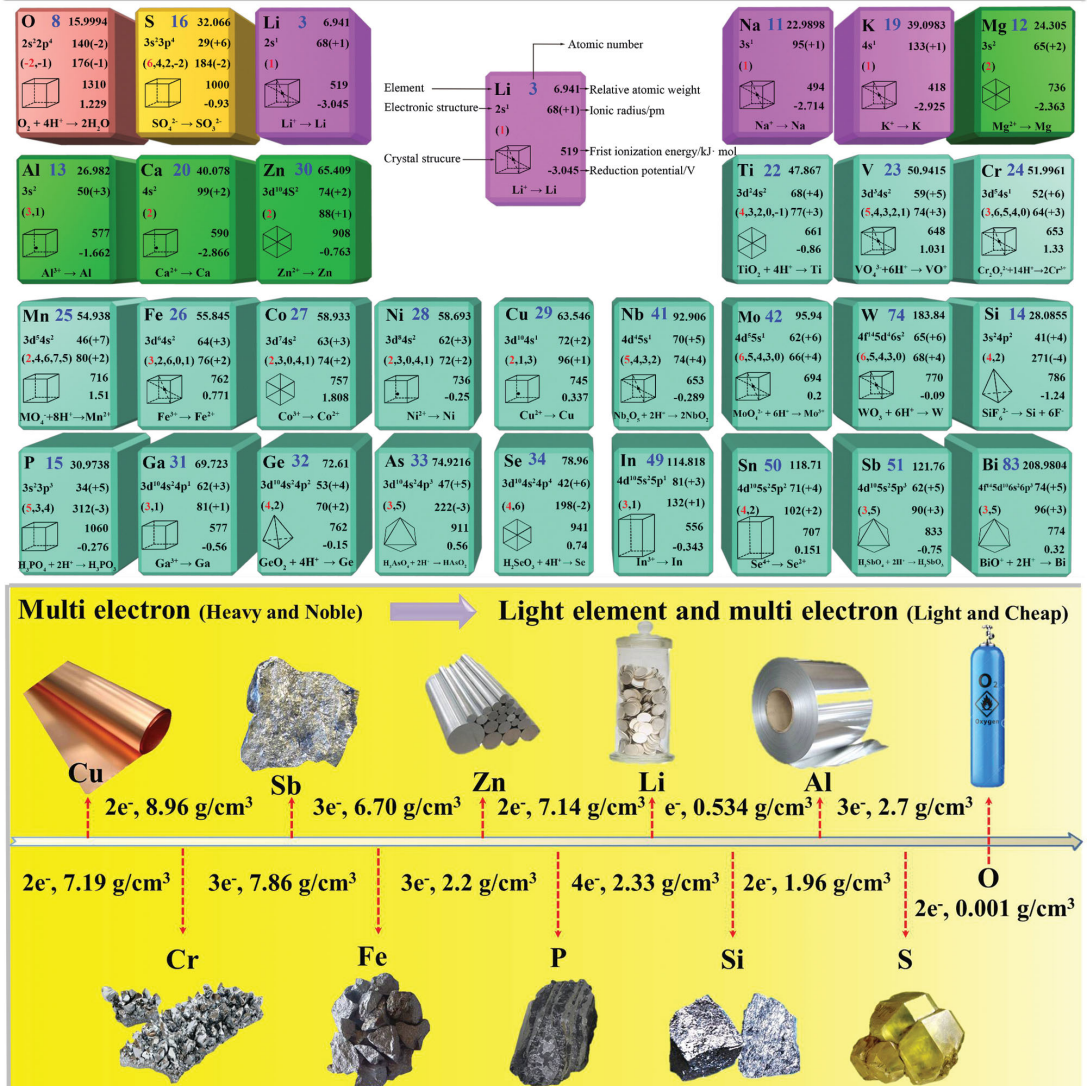
a) Definite characteristics of elements involved in multi‐electron reactions, b) future development trend for new battery technologies with multi‐electron reductions based on light and inexpensive elements.

Careful analyses of the periodic table of elements have been performed to explore active elements suitable for multi‐electron electrochemical reactions. **Figure** **1** and **Figure** **2** highlight the elements that have previously been reported to transfer more than one electron per mole of active material. The transition metals Cu, Fe, Cr, Co, Mn, Ni, V, Nb, and Mo have been considered as redox active elements that exchange more than one electron per transition metal as a result of their redox couples Ti^+2^/Ti^+4^, V^+2^/V^+5^, Cr^+2^/Cr^+6^, Mn^+2^/Mn^+4^, Fe^+2^/Fe^+4^, Co^+2^/Co^+4^, Ni^+2^/Ni^+4^, Cu^+1^/Cu^+3^, Nb^+3^/Nb^+5^, and Mo^+3^/Mo^+6^.^18^ However, layered lithium transition metal oxides exchange no more than one electron during their electrochemical process. Certain kinds of polyanionic compounds based on transition metals and polyanions (XO_4_)*^n^* ^−^ have been extensively studied and are known to exhibit the characters required for an multi‐electron reaction mechanism. Transition metal oxide‐based materials can be classified as conversion‐type active materials and have high theoretical capacity as anodes for LIBs and NIBs (sodium‐ion batteries). Active elements (such as Si, Ge, Sn, Pb, Sb, and P) capable of electrochemically alloying with Li and Na have high charge capacity owing to the relatively high stoichiometric ratio of Li that electrode can accommodate. A comparison of these capacities is shown in Figure 1g.

In these systems, Li and Na are charge carriers, and charge transfers from the variably valent transition metal cations upon Li and Na intercalation into the hosts. Thus, intercalation more than one Li or Na ion is required for multi‐electron transfer during the electrochemical process. Multi‐electron reactions can be achieved by insertion of a polyvalent cation. Comparisons of the physical properties of alkali metals such as Li, Na, and K, and multivalent cations such as Mg, Ca, Zn, and Al as charge carriers for rechargeable batteries are shown in Figure 1c,g. Anions can also take part into redox reactions, such as in Li–S batteries and metal–air batteries.^7^ Thus, the contribution of both cations and anions in a multi‐electron reaction is expected to break through the energy density bottleneck that Li‐ion batteries currently suffer from. We have divided the following discussion of secondary batteries into four parts, correlating to the different multi‐electron reaction types shown in Figure 1b.

Multi‐electron reaction materials are of obvious interest for high capacity battery materials. During the past few years, in‐depth investigation of multi‐electron mechanisms has promoted the further exploration of novel electroactive materials. Moreover, it need to meet new challenges of innovation of the design and optimization of new materials. It has been said that “technology is always limited by the materials available”, and this is reasonably applicable to battery technology and science at present.^4^ However, the way in which multi‐electron mechanisms may be applied in battery systems is still a challenge and any breakthrough will certainly bring about a great rush for battery applications in EES.

## 3 Multi‐Electron Reactions in LIBs and NIBs

**Figure 3:**
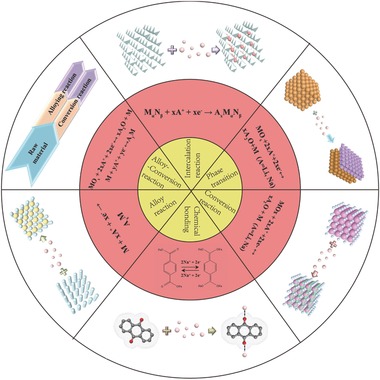
Scheme of reactions based on multi‐electron mechanisms in LIBs and NIBs.

Multivalent chemistries have been intensively researched since multi‐electron reactions were first proposed to offer the possibility of improving battery energy density.^6^ As mentioned above, for secondary batteries based on alkali metals such as Li and Na, multi‐electron reduction requires the insertion of more than one Li^+^ or Na^+^ into the host structure. This corresponds to a reduction in the valence of the metal redox center to maintain electroneutrality.^19^ Intercalation continues until structural expansion and electronic structural changes occur.[[qv: 15c]] In this section, the reaction mechanisms related with multi‐electron transfer are discussed. Six possible reversible energy storage mechanisms are listed in **Figure** **3** and further summarized.

### 3.1 Intercalation Reactions

Intercalation refers to the reversible intercalation of mobile guest ions such as Li^+^ or Na^+^ into a crystalline host lattice. Intercalation reactions involve layered host lattices owing to their structural flexibility and free adjustment of their interlayer spacing according to the geometry of the intercalated guest species.^20^ Thus, the intercalation reaction is accompanied by a series of changes in the structure of the host lattice during the electrochemical process.

Research on cathode materials for lithium‐ion batteries has been almost completely focused on layered LiCoO_2_ and LiNi_1/3_Co_1/3_Mn_1/3_O_2_. LiCoO_2_ is the most commonly used positive material, and has a high theoretical capacity of 274 mAh g^−1^. However, LiCoO_2_ yields a specific capacity of no more than 150 mAh g^−1^ because its structure tends to collapse when more than 50% of the Li‐ions have de‐intercalated from the host lattice.^4^, ^21^ As lithium is removed from Li*_x_*CoO_2_, Co^3+^ is oxidized to unstable Co^4+^, and a high concentration of Co^4+^ damages the cathode crystallinity and leads to a phase transformation.^22^ Efforts have been made to partially substitute Co with other 3d metals such as Ni and Mn to form LiNi_1/3_Co_1/3_Mn_1/3_O_2_, a more stable structure against collapse at higher voltage with a sustainably reversible capacity of 190 mAh g^−1^. However, during lithium insertion these 3d metal oxides still cannot reversibly change their oxidation state by accommodating more than one lithium ion per metal without the destruction of their crystalline structure. Intercalation in this review refers to the reversible intercalation of mobile guest ions such as Li^+^ and Na^+^ into the crystalline host lattice.

#### 3.2.1 V_2_O_5_

**Figure 4:**
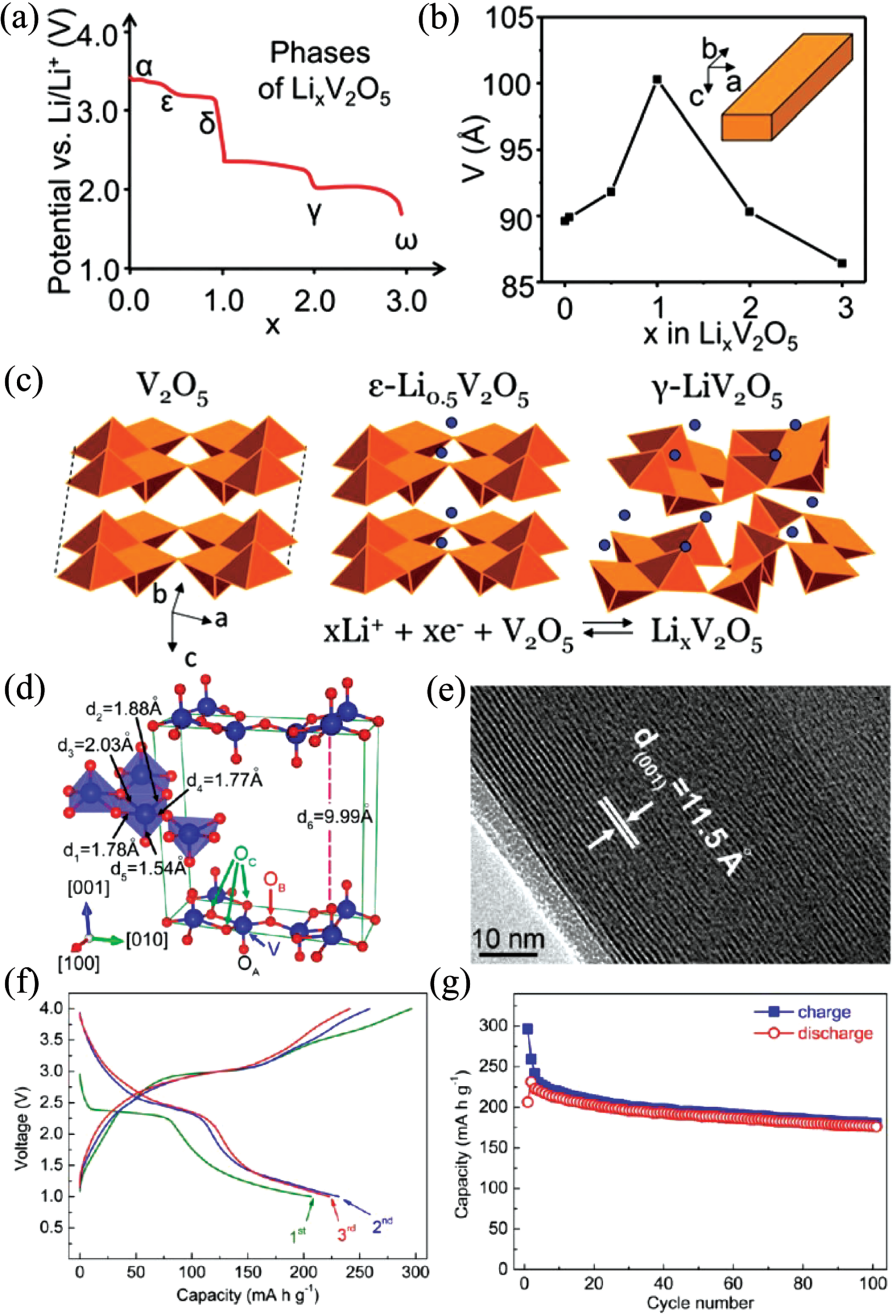
Li*_x_*V_2_O_5_ bronzes: a) potential‐stoichiometry dependence of a Li/V_2_O_5_ cell showing 5 phases. . b) Volume of the unit cell per V_2_O_5_ structure as a function of bronze stoichiometry, c) structures of pure V_2_O_5_ and two bronze phases showing puckering of the tetrahedral layers as a consequence of lithium intercalation. e) Crystal structure, f) lattice resolution TEM image, g,h) electrochemical performance of bilayered V_2_O_5_. a) Reproduced with permission.[[qv: 27c]] Copyright 1994, Elsevier. b,c) Reproduced with permission.^29^ Copyright 2015, Royal Society of Chemistry. e,f,g,h) Reproduced with permission.^37^ Copyright 2013, American Chemical Society.

Vanadium pentoxide (V_2_O_5_) belongs to the transition metal oxide family and is often employed as a secondary battery cathode. V_2_O_5_ has a layered structure held together by weak van der Waals interactions, described by a square‐pyramidal coordination of V^5+^ with five oxygen atoms (**Figure** **4**c).^23^ Thus, V_2_O_5_ is considered to be a typical intercalation compound. Intercalated guest ions are stored in the interspace between two VO_5_ pyramid layers.^24^ This layer host material has a theoretical capacity of 294 mAh g^−1^, corresponding to two lithium insertion/extraction, which is much higher than most commonly used cathode materials. In the potential range of 4.0–2.0 V vs Li/Li^+^, the reversible charging and discharging reactions can be written as:^25^

(6) $$V_{2}O_{5}+x{\mathrm{Li}}^{+}+xe^{-}↔{\mathrm{Li}}_{x}V_{2}O_{5}\;(x\approx 2.0)$$

Li–V_2_O_5_ undergoes several first‐order phase transformations during intercalation (as shown in Figure 4a). For example, the α‐phase of ${\mathrm{Li}}_{x}V_{2}O_{5}$ exists at *x* < 0.01, ε‐phase (3.36 V) at 0.35 < *x* < 0.7, δ‐ phase (3.15 V) at 0.7 < *x* < 1, and γ‐ phase (2.21 V) at 1 < *x* < 2.^26^ Further insertion of a third lithium into V_2_O_5_ leads to the irreversible formation of ω‐phase (2 < *x* < 3) ${\mathrm{Li}}_{x}V_{2}O_{5}$, which has a rock salt type structure.[[qv: 25b,27]] Reports have demonstrated that all these phases can be reversibly cycled in the stoichiometric range of 0 ≤ *x* ≤ 3, even though the structure undergoes irreversible transformation.^28^ Intercalation of lithium in the interlayer space leads to puckering of the layers and partial reduction of the oxidation state from +5 to +4 (shown in Figure 4c).^29^

Previous studies have indicated that the low diffusion coefficient of lithium ions and moderate electronic conductivity of crystalline V_2_O_5_ combined with structural change during the deep charge–discharge process hinders its development as cathode for secondary batteries.[[qv: 27b,30]] Electrochemical performance is influenced by a number of physicochemical properties such as structure and morphology. The fabrication of electrode materials in nanoscale and architectured forms is a common approach used to improve electrochemical performance. Nanostructured V_2_O_5_ modified towards a more open structure (such as nanorods,[[qv: 27a,31]] nanotubes,^26^, ^32^ nanosheets,^33^ hollow microspheres,^30^, ^34^ and hierarchical structures^35^) has been extensively studied to shorten the diffusion distance for both lithium ions and electrons and increase the electrode‐electrolyte contact area.^36^ For example, a highly integrated V_2_O_5_/PEDOT core–shell nanobelt array on 3D graphite foam was prepared as a high‐rate, ultrastable and freestanding cathode for lithium‐ion batteries.[[qv: 27b]] The nanoarray structure is favorable for accommodating the strain caused by Li^+^ insertion/extraction and is more desirable than powder nanostructures in terms of shorter Li^+^ diffusion and more direct electron transport. Moreover, the conductive PEDOT coating not only facilitates electron transfer and preserves the whole structure of the array during cycling, but also prolongs the discharge plateau above 3.0 V, resulting in an increased high‐voltage capacity and energy density. This special architecture results in an electrode that exhibits superior electrochemical performance.

V_2_O_5_ also shows great potential for NIBs. Few‐layered V_2_O_5_ can shorten the diffusion distance for sodium ions. Single‐crystalline bilayered V_2_O_5_ nanobelts have been successfully synthesized and used as a cathode material for NIBs.^37^ Bilayered V_2_O_5_ nanobelts with a large (001) interlayer spacing of 11.53 Å (along the c‐axis) can accommodate Na insertion and extraction. The crystal structure is described in Figure 4e. Such V_2_O_5_ nanobelts have exhibited a high capacity of 231.4 mAh g^−1^ at 80 mA g^−1^ (see Figure 4f,g) as a cathode material in Na‐ion batteries, which is very close to the theoretical capacity of 236 mAh g^−1^ (corresponding to the formation of Na_2_V_2_O_5_).

Under the classical charge‐storage mechanism discussed above, only 2 Li^+^ or 2 Na^+^ can be inserted into crystalline V_2_O_5_. It is worth noting that V_2_O_5_ aerogel stores charge through a special mechanism which enables it to reversibly uptake more than 2 Li^+^. Chemical lithiation studies have shown that up to 5.8 equivalents of Li per mole of V_2_O_5_ aerogel can be inserted, corresponding to a specific capacity of 650 mAh g^−1^.^38^ A recent investigation reported that V_2_O_5_ aerogel delivered 345.5 (corresponding to the insertion of 2.3 equivalent of Li^+^ per mole of host material), 324, 291, and 240 mAh g^−1^ at C/20, C/10, C/4 and 1 C rate, respectively. When used as a sodium‐ion battery cathode, the electrode delivered about 150 mAhg^−1^, corresponding to the insertion of 1 Na^+^ equiv. per mole of V_2_O_5_, indicating a more pronounced spread of sodium‐site energies.^39^

The electrochemical studies show that V_2_O_5_ aerogels provide enhanced capacity for both lithium and polyvalent cation (corresponding electrochemical behavior will be discussed in Section 4) as compared to the corresponding crystallites. The reason for this special behavior is associated with the mesoporous nature of the material which enable the diffusion distances to be traversed by Li^+^ are quite small as kinetic within the solid phase is diffusion‐limited.^40^ Moreover, strong capacitive response was observed when employing the sticky‐carbon electrode for V_2_O_5_ aerogels.^41^ The issue is that how the enormous surface area contributes to the enhanced capacity for lithium. XAS (X‐ray absorption spectroscopy) and XPS (X‐ray photoelectron spectroscopy) results showed that little change in the oxidation state of the vanadium is observed and the species of V^3+^ do not formed during Li^+^ electrochemically inserts into V_2_O_5_ aerogel. This special storage mechanism can be explained by Ruetschi's cation‐vacancy hypothesis, even though the existence of cation vacancies has not been directly confirmed through high‐resolution imaging.^42^ Aerogels amplify the surface character of the metal oxides, defects and cation vacancies contribute strongly to the electrochemical properties of aerogels,^43^ The accessibility of Li^+^ to cation vacancies in V_2_O_5_ aerogel would produce an ion current but no accompanying electron transfer to vanadium cations thus no generation of V^3+^. The above mechanism would explain why V_2_O_5_ aerogel could uptake more than 2 Li per V_2_O_5_ and the high capacity obtained with lithium and polyvalent cations.^44^ Other examples of this enhancement include the charge‐storage properties of certain‐deficient oxides such as γ–MnO_2_ and γ–Fe_2_O_3_.[[qv: 42a]] Furthermore, V_2_O_5_ aerogel can also serve as a host structure for polyvalent cations such as Mg^2+^, Al^3+^, and Zn^2+45^ and cations with an ionic radius significantly larger than that of Li^+^ such as K^+^ and Ba^+^.^46^

Some metal oxides in the large family of Wadsley–Roth‐type phases with ReO_3_ type structure like Nb_2_O_5_ can insert about 14 Li per host metal at ambient temperature with minimal structural distortion.^47^ Another, PNb_9_O_25_, exhibits a shear structure, the electrochemical insertion of lithium reaches a value of *x* ≈ 13.5 between 3.0 and 1.0 vs Li+/Li at a slow cycling rate.^48^ With reduction of Nb^5+^ to Nb^4+^ and then partly to Nb^3+^, the the lattice parameters undergo a total change in variation of ∆*V*/*V* = +10%. Other shear structures^49^ like HNb_2_O_5_, GeNb_18_O_47_, VNb_9_O_25_ also exhibit a similar multi‐electron transfer/multi‐Li‐insertion mechanism.

### 3.2 Phase Transition Reactions

During charge and discharge, the reactants transforms from the initial phase to another, such as LiMPO_4_, and Li_4_Ti_5_O_12_. The most‐studied polyanionic compounds, such as phosphates and silicates, have been proposed as an alternative to lithium transition metal oxide cathodes. Polyanionic compounds possess specific advantages, such as increased safety, higher thermal stability, owing to strong covalently bonded oxygen atoms, and the possibility of using cheaper, non‐toxic precursors. 3D frameworks built on transition metals and polyanions (XO_4_)*^n^* ^−^, in which tetrahedral (XO_4_)*^n^* ^−^ and MO_6_ octahedra combine via strong covalent bonding, have become a very extensive area of cathode research besides oxides.^50^ However, constraints on operating voltage owing to the stability of organic electrolytes, as well as to cathode structural stability, have made the multi‐electron transfer process difficult to achieve. Much effort has been made to find polyanionic compounds permitting multi‐electron reactions using multivalent active transition metals.

Phosphates (LiMPO_4_, M = Fe, Mn, Co, Ni, or V), which contain phosphorus atoms tetrahedrally coordinated by oxygen, have been the most intensively studied. LiFePO_4_ was first reported by J. Goodenough^51^ and was later more widely studied and fully reported. For the Fe^+2^/Fe^+4^ two‐electron redox couple, only the Fe^3+^/Fe^2+^ redox energy lies close to the Fermi energy; the +3/+4 voltage is too high for current electrolytes, thus restricting it to a suitable level of 3.4 V that allows one Li^+^ reversible extraction/insertion per formula unit. This voltage issue excludes the practical use of LiFePO_4_ based on the +2/+4 couple.

Another extensively studied polyanion compound is Li_3_V_2_(PO_4_)_3_ (LVP), which has two polymorphs, i.e., rhombohedral and monoclinic phases. Rhombohedral Li_3_V_2_(PO_4_)_3_ was first prepared by ion exchange from Na_3_V_2_(PO_4_)_3_ and first investigated for its electrochemical behavior upon Li extraction.[[qv: 50b]] The charge–discharge profile of Li_3_V_2_(PO_4_)_3_ confirms that two electrons can be extracted during the first charge process, the potential of which is up to 4.1 V. However, a polarization is observed in the discharge curve and only 1.3 Li are reinserted. These results suggest a kinetic limitation to the reinsertion of Li, which is caused by structural change upon the extraction of two Li^+^. The monoclinic phase (theoretical capacity of 197 mAh g^−1^) is thermodynamically stable and preferable for use as a cathode owing to its 3D open framework for fast lithium ion diffusion and accommodation of up to 2 Li^+^ in the 3.0–4.3 V vs Li/Li^+^ window.^52^

**Figure 5:**
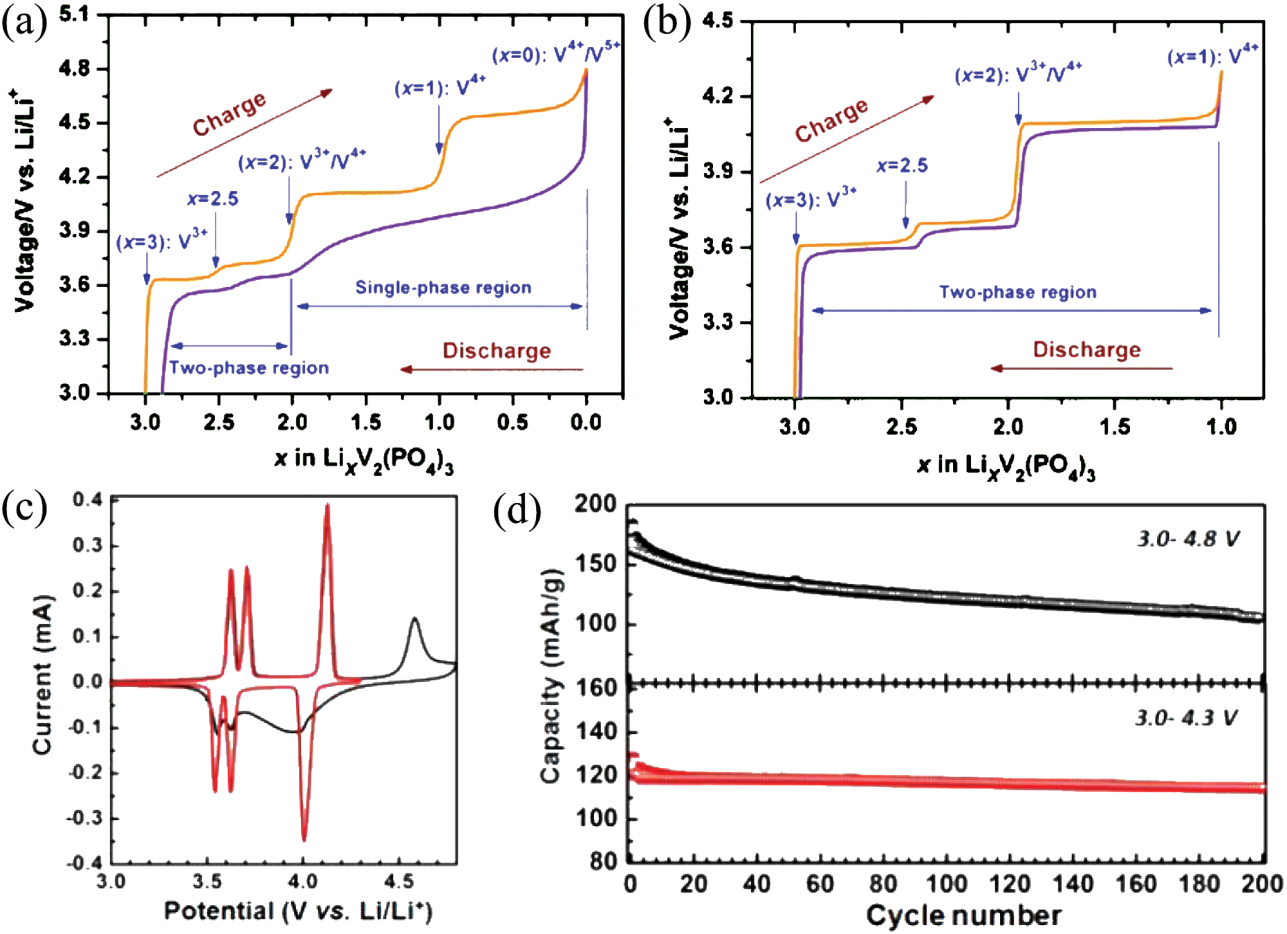
The electrochemical voltage‐composition curves of Li_3_V_2_(PO_4_)_3_ in the voltage ranges of a) 3.0‐4.8 V and b) 3.0–4.3V vs Li/Li^+^. c) Cyclic voltammetry (CV) curves at a scan rate of 0.1 mV·s^−1^, and d) cycling performance at 0.1C in the two different voltage windows of 3.0−4.8 V (black) and 3.0−4.3 V (red). a,b) Reproduced with permission.[[qv: 53a]] Copyright 2014, Elsevier. c,d) Reproduced with permission.[[qv: 52a]] Copyright 2015, American Chemical Society.

In this window, no solid solution region is observed. The corresponding phase transition processes of Li_3_V_2_(PO_4_)_3_ → Li_2.5_V_2_(PO_4_)_3_ (≈3.6 V vs Li/Li^+^), Li_2.5_V_2_(PO_4_)_3_ → Li_2_V_2_(PO_4_)_3_ (≈3.7 V vs Li/Li^+^), and Li_2_V_2_(PO_4_)_3_ → LiV_2_(PO_4_)_3_ (≈4.1 V vs Li/Li^+^) are exhibited in **Figure** **5**B.^53^ A wider voltage window of 4.8 V allows for the extraction of three Li^+^.[[qv: 52b,54]] The third lithium, extracted at voltage of 4.6 V vs Li/Li^+^, induces a two‐phase reaction between LiV_2_(PO_4_)_3_ and V_2_(PO_4_)_3_ associated with partial oxidation of V^4+^ to V^5+^.^55^ V(1) and V(2) in V_2_(PO_4_)_3_ display fairly close average bond distances and average vanadium valencies of +4.5, resulting in a disordered lithium reinsertion (seen in Figure 5A characterized as solid‐state region). However, a higher voltage such as 4.8 V causes structural instability and side reactions between the electrolyte and Li*_x_*V_2_(PO_4_)_3_, further leading to rapid specific capacity fading.^56^ A comparison of the electrochemical performance of these systems is shown in Figure 5c,d.

Orthosilicates (Li_2_MSiO_4_, M = Fe, Mn, Co, Ni) are another type of polyanion cathode material. The 3d metal elements usually have multiple oxidation states, which allow for multi‐electron reactions during the charge–discharge process. Orthosilicates are able to allow the transfer of more than one electron per 3d metal owing to their special framework.^57^ The (SiO_4_)^4−^ group can theoretically allow the 3d metal to exchange its valence state between +2 to +4, thus enabling two lithium to be reversibly de‐/inserted during the electrochemical process.[[qv: 50a]] Li_2_FeSiO_4_ provides the ability to exchange two‐electron reaction for Fe (II) to Fe (IV) with a theoretical specific capacity of 331 mAh g^−1^. During Li^+^ extraction/insertion between Li_2_FeSiO_4_ and LiFeSiO_4_ (i.e., oxidation of Fe^2+^ to Fe^3+^), the volume change of the crystal structure is reported to be only 2.8%.^57^ If the second Li ion is extracted from the host structure without the further oxidation of Fe^3+^, the resulting significant structural collapse of the FeSiO_4_ leads to drastic capacity fading. Moreover, the calculated de‐intercalation potential of the second Li ion in Li_2_FeSiO_4_ is 4.79 V, which is beyond the limit of most electrolytes. Hence, this two‐electron reaction is hard to access at room temperature. Intergrown nanocomposite Li_2_FeSiO_4_•LiFePO_4_–C has been successfully synthesized using the strategy of introducing an electrochemically active component, LiFePO_4_, to Li_2_FeSiO_4_. This composite has been reported to reversibly react with 1.26 Li at 15°C and 1.72 Li at 45 °C.^58^

Another key member of the silicate family is Li_2_MnSiO_4_, which has a high theoretical capacity of 333 mAh g^−1^ based on the Mn^2+^/Mn^3+^/Mn^4+^ redox couples.^59^ Li_2_MnSiO_4_ exhibits a number of different structures, namely, orthorhombic forms (*Pmn*2_1_ and *Pmn*b) with two‐dimensional pathways for Li‐ion diffusion and monoclinic forms (*P*2_1_/n and *P*n) with Li‐ion pathways that are interconnected in three dimensions.^60^ The various structures possess similar electrochemical performance and electronic conductivity but varying ionic conductivity owing to the differently interconnected lithium sites of the different polymorphs. The calculated average 2Li^+^ intercalation potentials for Pmnb, Pmn2_1_, and P2_1_/n are are 4.18, 4.19, and 4.08 V, respectively.^57^, ^61^ However, transfer of only 1.56 electrons, corresponding to a high discharge capacity of 257.1 mAh g^−1^, was achieved during the first cycle, and the discharge capacity suffered drastic fading on subsequent cycles.[[qv: 59a]] When more than 1.66 Li^+^ was delithiated from the host, the crystalline structure could not be recovered after the subsequent discharge. The main reason for this large irreversible capacity and drastic capacity fading of Li_2_MnSiO_4_ is the formation of amorphous‐like regions and continuous changing of the lattice parameters during the extraction and insertion of Li ions. Another factor leading to the destruction of the crystalline structure is the Jahn–Teller effect associated with Mn^3+^ of Li_2_MnSiO_4_. Thus, limited progress has been attained for Li_2_MnSiO_4_ so far.

Because (BO_3_)^3−^ is the lightest polyanion group, borate compounds would be very attractive cathode materials. Although LiMBO_3_ (M = Mn, Fe, Co) cathode materials have been easily synthesized by solid state reaction,^62^ they have shown very poor electrochemical performance corresponding to less than 0.04 Li reversibly inserted/de‐inserted per formula unit.

The key limiations of polyanion compounds have been their intrinsic low electronic conductivity and slow lithium‐ion diffusion rate. Various methods have been adopted in attempts to improve their conductivity: 1) introducing guest atoms into the crystal structure;^63^ 2) reducing the particle size;^64^ 3) coating with electrochemically conductive materials,^65^ especially carbon coating. Carbon coating is the most effective strategy because it can contribute towards the control of the growth of the particle, thus making the particle more homogeneous, and also forms a conductive network, which is favorable for electron and ion transfer.^66^

Meanwhile, it is known that the choice of synthetic method can greatly affect the structure and morphology, and subsequently the electrochemical performance of cathode materials. Thus, the synthesis–structure–performance relationship of novel electrode materials should be further investigated. Investigations in the development of polyanion materials into matured electrode materials that to meet the requirements of high energy density are still in their early stages. Although polyanion compounds provide the possibility of multi‐electron reaction with specific characteristics, the development of stable polyanion frameworks for hosting multi‐electron reactions is still a challenge. As discussed above, in the 3.0–4.5 V voltage window, active redox couples Fe^2+^/Fe^3+^, Mn^2+^/Mn^3+^, and Cu^1+^/Cu^2+^ exhibit only one‐electron reactions combined with the safety requirements for phosphate. Only vanadium (V^3+^ to V^5+^) and molybdenum (Mo^3+^ to Mo^6+^) have redox couples that could provide two‐electron activity in phosphates. Hence, much improvement could be expected for multi‐electron phosphates if the voltage window could be increased to a larger region. A voltage design strategy, computed by state‐of‐the‐art ab initio calculations, involving mixing a metal active on the +2/+3 couple (e.g., Fe) with an element which can be active at up to +5 or +6, such as V and Mo, has been proposed.^67^ With this strategy more than one Li atom per transition metal could be theoretically exchanged, giving a higher theoretical capacity. However, the overall expected gain in energy density is not clear owing to the “weight penalty” arising from the presence of polyanion groups.^65^

#### 3.2.1 Li_4_Ti_5_O_12_

**Figure 6:**
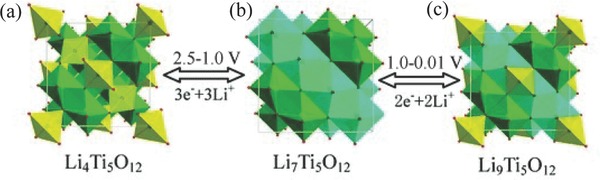
The reversible insertion/de‐insertion process within 2.5–0.01 V vs Li/Li^+^.

Spinel Li_4_Ti_5_O_12_ (LTO) is considered to be a promising anode material for Li‐ion batteries because of its excellent characteristics such as low cost, safety, negligible volume change during the charge–discharge process, and long life cycling.^68^ The lattice of LTO is a spinel framework structure with the F*d*3–m space group, which forms a 3D tunnels with octahedral sites sharing edges.^69^ The tetrahedral 8a sites of the framework are occupied by Li^+^ ions, while the octahedral 16d sites are occupied by both Li^+^ ions and Ti^4+^ ions randomly, with a Li^+^:Ti^4+^ ratio of 1:5.[[qv: 69b,70]] Spinel‐LTO can be described as Li_3(8a)_[LiTi_5_]c_(16d)_O_12(32e)_ (crystal units as shown in **Figure** **6**a). LTO can accommodate up to three Li^+^ to form Li_7_Ti_5_O_12_ (≈1.55 V vs Li^+^/Li), leading to a theoretical capacity of 175 mAh g^−1^. The corresponding electrochemical reaction can be expressed as shown in Equation:

(7) $${\mathrm{Li}}_{4}{\mathrm{Ti}}_{5}O_{12}+3{\mathrm{Li}}^{+}+3e^{+}↔{\mathrm{Li}}_{7}{\mathrm{Ti}}_{5}O_{12}$$

Reduction of three Ti^4+^ to Ti^3+^ occurs during insertion, causing a phase transition from spinel LTO to rock‐salt‐Li_4_Ti_5_O_12_.^70^, ^71^ The lithiation process causes Li^+^ to transfer from 8a to 16c sites, resulting in an end composition of Li_6(16c)_[LiTi_5_]_(16d)_O_12(32e)_ (crystal units as shown in Figure 6b). The lattice constants of spinel‐LTO and rock‐salt‐LTO have been calculated to be 0.8364 nm and 0.8353 nm, and the volume change during conversion between the two phases is only 0.2%. For this reason, LTO is referred to as a zero‐strain material.^71^, ^72^ The reaction is a typical two‐phase conversion during the lithiation/delithiation process, and thus the charge–discharge curves are characterized by a flat and extended voltage plateau at around 1.55 V.^73^

It should be noted that spinel‐LTO is a good lithium‐ion conductor because of the existence of available free octahedral sites in its lattice structure, but a poor electronic conductor because the oxidation state of Ti in spinel‐LTO is +4.^74^ Thus, its inherently low electronic conductivity of <10^−13^ S cm^−1^ at room temperature hinders the electrochemical performance of spinel‐LTO. Reported strategies such as surface coating with conductive species, doping, and fabrication of nanostructured materials are effective ways to improve its conductivity.^72^

As stated previously, spinel‐LTO can accommodate up to three lithium ions, giving a theoretical capacity of 175 mAh g^−1^ in the voltage range 1.0–2.5 V. However, some researchers have reported that spinel‐LTO can deliver a theoretical capacity of 293 mAh g^−1^ within the voltage range 2.5–0.01 V.^75^ Li‐ion insertion can occur at (8a), (16c), and (48f) sites at low potentials (<1.0 V vs Li/Li^+^), and the additional Li^+^ inserts at octahedral (16c) and tetrahedral (8a) sites.^76^ The other 2 mol of Li‐ions intercalated into Li_7_Ti_5_O_12_ at 1.0–0.01 V vs Li/Li^+^ as can be presented as:

(8) $$\begin{array}{l} {\mathrm{Li}}_{6\left(16c\right)}{\left[{\mathrm{LiTi}}_{3}{}^{3+}{\mathrm{Ti}}_{2}{}^{4+}\right]}_{\left(16d\right)}O_{12\left(32e\right)}+2e^{-}+2{\mathrm{Li}}^{+}\\ \;↔{\mathrm{Li}}_{2\left(8a\right)}{\mathrm{Li}}_{6\left(16c\right)}{\left[{\mathrm{LiTi}}_{5}{}^{3+}\right]}_{\left(16d\right)}O_{12\left(32e\right)} \end{array}$$

The additional two Li‐ions inserted at vacant tetrahedral sites at low potential enhance the reversible capacity to 215.1 mAh g^−1^ within 2.5–0.01 V vs Li/Li^+^ at 0.2 C. The whole insertion process is shown in Figure 6. The theoretical capacity of LTO is limited by the number of tetravalent titanium ions, not the number of octahedral or tetrahedral sites available to accommodate lithium ions. This explains why the maximum number of lithium ions taken up by LTO is five even though L_9_T_5_O_12_ still has tetrahedral (8a) sites.^75^

Very recently, spinel Li_4_Ti_5_O_12_ has also been reported as an anode material for Na‐ion batteries.^77^ During the sodium insertion process, three Na ions can be inserted per mole LTO, and the Na^+^ insertion/extraction potential is ≈0.91 V. The reaction can be represented as:

(9) $$2{\mathrm{Li}}_{4}{\mathrm{Ti}}_{5}O_{12}+6{\mathrm{Na}}^{+}+6e^{-}\to {\mathrm{Li}}_{7}{\mathrm{Ti}}_{5}O_{12}+{\mathrm{LiNa}}_{6}{\mathrm{Ti}}_{5}O_{12}$$

**Figure 7:**
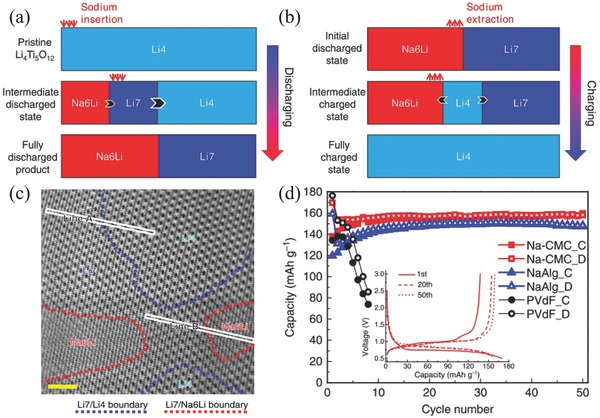
a) Discharging and b) charging processes of LTO in a sodium‐ion batteries; c) ABF image in the half electrochemically sodiated LTO; d) electrochemical performance of LTO in sodium‐ion batteries. Reproduced with permission.[[qv: 77b]] Copyright 2013, Nature Publishing Group.

The sodium insertion process can be explained by a three‐phase separation mechanism, during which Na^+^ ions occupy 16c sites exclusively to form a Na_6_Li phase, while at the same time, Li_8a_ ions are pushed to the nearest neighbor Li4 phase to form a Li7 phase, thus two new phases are created (as shown in **Figure** **7**a).[[qv: 77b]] Spinel‐LTO shows a stable specific capacity of 155 mAh g^−1^ with Coulombic efficiency >99 %, even though the lattice volume expansion is 12.5 % (Figure 7d).

The diffusion of Na^+^ in the structure requires a large barrier to be overcome and the Na^+^ diffusion coefficient of Li_4_Ti_5_O_12_ is remarkably low (10^−16^ cm^2^ s^−1^)[[qv: 77a]] compared with that of Li^+^ (10^−13^–10^−9^ cm^2^ s^−1^).^78^ The inherently poor electric conductivity of Li_4_Ti_5_O_12_ can be resolved by forming the Li_4+δ_Ti_5_O_12_ phase, which has a higher conductivity during Li^+^ and Na^+^ intercalation. Li_4_Ti_5_O_12_ has a relatively low energy density when compared with conventional anodes such as hard carbon, but its high operating voltage dispels the concern of metal dendrite formation. As an anode, the practical specific capacity of LTO is about 150–160 mAh g^−1^, which is lower than that of other anode materials such as graphite. Strategies that have been reported to improve the anode performance of LTO include nanostructuring,[[qv: 77a,78,79]] lattice ion‐doping with metal cations[[qv: 69a,80]] such as Co^3+^, V^5+^, Cu^2+^, and Mn^4+^, non‐metallic ion doping^81^ including with Br^−^, Cl^−^, and F^−^, or surface coating.^82^

### 3.3 Reversible Reactions of Organic Compounds

Organic electrode materials usually provide a high transfer electron number, low weight, and flexible structure resulting in a great potential for high energy density and rapid charge and discharge.^83^ Such organic materials are considered as promising electrodes for superior batteries because of the following reasons: (i) Alkali metal ion insertion/extraction in inorganic materials are mainly controlled by the reaction kinetics, while such concerns are not present for organic electrodes. Surprising rate performance can be delivered as a result. (ii) Some alkali metal ions with large ionic radius are difficult to insert into inorganic materials. Organic materials with flexible molecular chains may adjust their structure as their ionic size changes. (iii) The main components of organic molecules are C, H, O, N, and so on, which are very light and small, leading to high weight energy density. These kinds of materials need to be systematically studied and exploited for energy storage devices in the future.

Although the mechanisms of sodium ion storage in the following two kinds of organic materials are different, previous studies have found that both can be used as electrode materials with two‐ or multi‐electron transfer during charge and discharge processes. Especially for Na‐ion batteries, these flexible host materials can provide a retractable space for storage of the large ionic radius Na ions.

#### 3.3.1 Organic Chemical Bonding Reactions1

**Figure 8:**
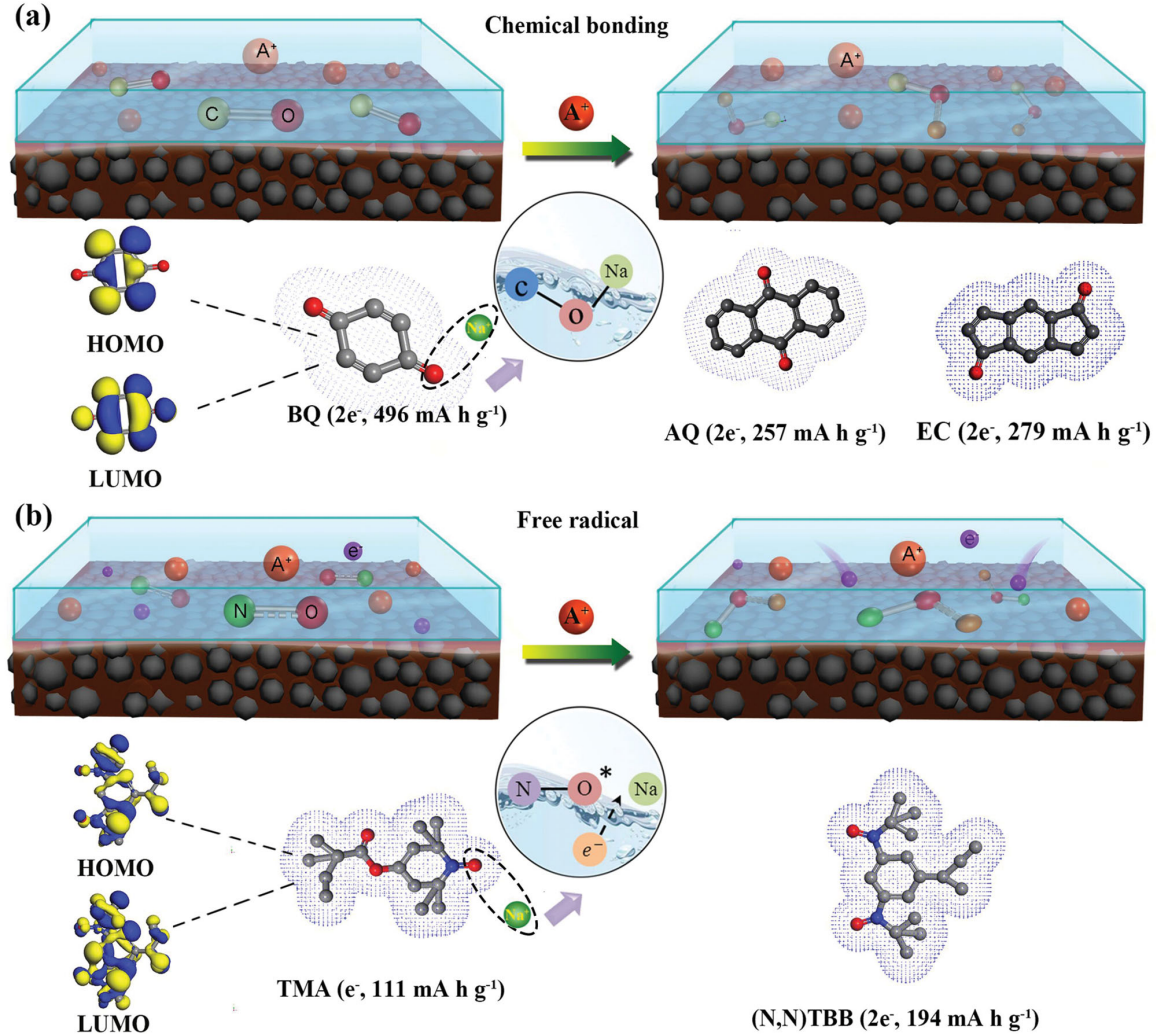
Highest occupied molecular orbital (HOMO), lowest unoccupied molecular orbital (LUMO), molecular structure and theoretical specific capacity of the representative a) organic carbonyl and b) radical compounds.

**Figure 9:**
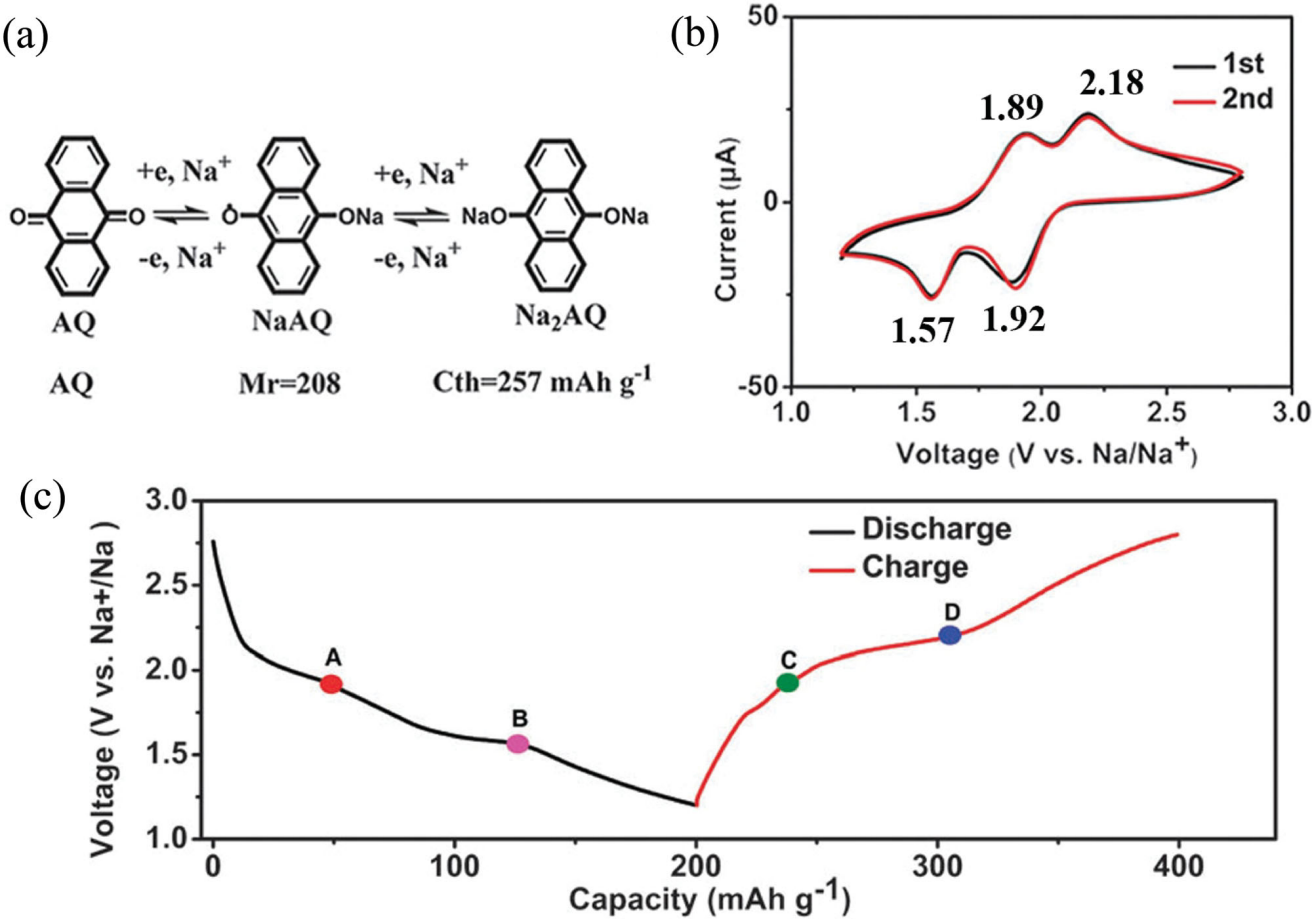
a) Electrochemical redox reaction mechanism, b) CV curves and c) initial charge–discharge curve of anthraquinone (AQ) which was tested as cathode for sodium ion battery. Reproduced with permission.[[qv: 84b]] Copyright 2015, Royal Society of Chemistry.

Organic carbonyl compounds represent the earliest discovered organic electrode materials. They deliver a good multi‐electron transfer process and high theoretical specific capacity. The sodium storage mechanism of carbonyl compounds is regarded to be a reversible chemical bonding reaction that involves the breakage of the C=O bond and connection between Na and O (inset in **Figure** **8**a). It is worth noting that the relationship between the electrochemical window and highest occupied molecular orbital (HOMO) and lowest occupied molecular orbital (LUMO) values of these organic compounds is different to that of the electrolyte. Among them, anthraquinone (AQ), benzoquinone (BQ), and ethoxycarbonyl (EC) based materials are emerging as promising choices for organic electrodes.^84^ Taking the redox reaction of an AQ‐based material used as cathode for sodium ion batteries as an example, the further mechanism is shown in **Figure** **9**a.

As shown in Figure 9a, AQ electrodes can deliver two electrons through a multi‐step reaction corresponding to the redox peaks at 1.89/1.57 V and 1.92/2.18 V, respectively. As a result, these electrodes can provide theoretical capacities greater than 250 mAh g^−1^.

Certainly, there are some problems with organic carbonyl electrode materials. First, the conductivity of these materials is very poor. Most organic materials do not have the ability to transmit electrons, except some conducting polymers. However, even if these conducting polymers can conduct electricity, their conductivity is not very good. Second, the dissolution problem of small molecules is fatal to the use of organic electrodes. Small molecule based electrode materials easily dissolve in the organic electrolytes used in most rechargable batteries, leading to poor cycling performance.

Carbon materials have high electronic conductivity, which can effectively promote the alkali metal ion diffusion kinetics of organic materials. Chen and co‐workers reported that a AQ/CMK‐3 cathode for sodium ion batteries had an optimized mass ratio of 1:1.[[qv: 84b]] The composite showed a discharge capacity of 214 mAh g^−1^ at 0.2C and maintained 88% of its initial capacity after 50 cycles. Compared with pure AQ, which delivered an initial capacity of 178 mAh g^−1^ at the same current density and retained 71% of its initial capacity after 50 cycles, it represented an obvious improvement. A poly(anthraquinonyl sulfide) (PAQS)–graphene composite electrode synthetized by Wang et al. was found to discharge about 50% of its theoretical capacity within a very short period of 16 s.^85^

Using polymerization to increase the molecular weight of the material can restrict the dissolution of small molecules. Poly(2,5‐dihydroxy‐1,4‐benzoquinone‐3,6‐methylene) (PDBM), reported by Owen et al.,^86^ exhibited an enhanced cycling stability which retained nearly 110 mAh g^−1^ after 100 cycles. In contrast, latest research results have shown that there is a nanoeffect for Na_2_C_8_H_4_O_4_ anodes that causes the insertion/extraction process of Na^+^ to involve a one‐step electron transfer mechanism.^87^ Because the formation of an intermediate was omitted during the charging/discharging process, the dissolution of the anode material was limited and therefore the cycling stability was enhanced. Furthermore, using organic lithium salts instead of inorganic salts can reduce the solubility of organic molecules in the electrolyte.^88^ The development of an all‐solid‐state battery will thoroughly solve the organic material dissolution problem. For example, a Pillar^85^ quinone (P5Q) cathode in an all‐solid‐state battery^89^ displayed an average operating voltage of 2.6 V and amazing capacity of 418 mAh g^−1^. Its unprecedented cycling stability meant that 94.7% of its initial capacity was retained after 50 cycles at 0.2C.

#### 3.3.2 Organic Free Radical Mechanism

In recent years, organic radical polymers—which mainly refers to aliphatic or non‐conjugated polymers—have attracted wide attention owing to their high charge and discharge capacity, excellent rate performance, and long cycle‐life. These advantages are attributed to the electrochemically active “nitroxide radical” group, which has rapid electron‐transfer ability and high chemical stability.^90^ The sodium storage mechanism of these polymers is described as shown in inset in Figure 8b. Because radical groups are neutral and without excess electrons, the ions of the electrolyte need to compensate for the unbalanced state at electrode upon massive electron transfer. The charge transfer numbers and theoretical capacities of a number of organic compounds are shown in Figure 8b.^91^

The first reported organic radical cathode was poly(2,2,6,6‐tetramethylpiperidinyloxy‐4‐yl methacrylate) (PTMA).[[qv: 91c]] It was reported to exhibit a high operating voltage of 3.6 V, high capacity (70% of the theoretical capacity) and long cycle life (retained above 90% of initial capacity after 500 cycles). In addition to being used as a cathode material, some radical polymers have also been proved to function as anode materials. For example, the poly(nitroxylstyrene)s produced by Nishide et al. could be used as both positive and negative electrodes.[[qv: 91b]]

Although there are a lot of highlights to organic radical materials, the development of these batteries is still subject to some problems. The most important factor is their poor conductivity. In an attempt to overcome this problem, poly(2,2,6,6‐tetramethylpiperidinyloxy‐4‐ylmethacrylate) (PTMA)–Ketjenblack developed by Lemmon et al. achieved a good conductivity determined by electrochemical impedance spectroscopy (EIS).[[qv: 91a]] Compared with pure PTMA (specific capacity of around 110 mAh g^−1^ after 5 cycles), the composite delivered an increased initial capacity of about 300 mAh g^−1^ and about 250 mAh g^−1^ after 100 cycles. The multi‐electron transfer process of PTMA can be illustrated as the transformation of an n‐type aminoxy anion into a p‐type oxoammonium cation through an intermediate with one free radical. Overall, the capacities of organic radical materials still need to be further improved.

### 3.4 Alloying–Dealloying Reactions

Metals (Sn, Pb, Bi), metalloids (Si, Ge, As, Sb), and polyatomic nonmetal P can alloy with lithium and sodium or form A‐Me (Me = Sn, Pb, Bi, Si, Ge, As, Sb, P) binary compounds.[[qv: 9b]] These electrode materials have the ability to interact with more than one lithium and sodium per metal atom, leading to a much higher specific capacity than that of common carbon anodes. Thus, these materials seem to be ideal candidates for the next generation of high energy density anode materials.

As in the case for Li‐ion batteries, alloy materials as electrodes have been extensively studied in the past decades because of the much higher reversible capacity than that of the Li‐graphite systems (Li_4.4_Si:4200 mAh g^−1^, Li_4.4_Ge: 1600 mAh g^−1^, Li_4.4_Sn:990 mAh g^−1^, Li_3_Sb:665 mAh g^−1^, Li_3_P: 2596 mAh g^−1^). As in the case of Li‐ion batteries, alloy based electrodes have been extensively studied in past decades because of their much higher reversible capacity than that of Li‐graphite systems (Li_4.4_Si: 4200 mAh g^−1^, Li_4.4_Ge: 1600 mAh g^−1^, Li_4.4_Sn: 990 mAh g^−1^, Li_3_Sb: 665 mAh g^−1^, Li_3_P: 2596 mAh g^−1^).^92^ Si and Sn have attracted more attention for Li‐ion batteries than Ge and Sb because of their better electrochemical performance, including their higher specific capacity and lower redox potential. Certainly, a lower redox potential anode material is beneficial for achieving higher energy density. Moreover, Ge and Sb are more expensive than Si and Sn. As for sodium‐ion batteries, the redox potentials of Si and Sn are very close to that of Na metal, which is higher than that of Li metal by 0.3 V. That is, their redox potentials, close to 0 V versus Na^+^/Na, are detrimental to Na‐ion batteries because the resulting large polarization imposes kinetic limitations on the electrochemical insertion of Na ions, which causes rapid capacity fading. Additionally, no experimental report has yet been published on the electrochemical sodiation of Si‐based materials, although it is known that Si can theoretically alloy with Na based on the formation of NaSi phase.[[qv: 9b,13a,93]] Note that Na alloying compounds possess about two‐fold lower capacity density than Li alloying compounds at the same volume expansion rate. This is because the ionic radius of Na is considerably larger than that of Li. The above issues indicate the significant challenges in employing alloying compounds as negative electrodes.

#### 3.4.1 Si

Silicon is an attractive material for anodes in energy storage devices, and can store several lithium atoms through the growth of a Li‐rich amorphous product:

(10) $$\mathrm{Si}+x\mathrm{Li}↔{\mathrm{Li}}_{x}\mathrm{Si},\;x\approx 3.75$$

This leads to an extremely high theoretical lithium storage capacity of 3579 mAh g^−1^ at room temperature.^6^, ^94^ However, the electrochemical lithiation of silicon to form lithium silicide Li_15_Si_4_ (Li_3.75_Si) at full lithiation could have a significant negative impact on stress generation and fracture, leading to capacity fading.[[qv: 8a,94a,95]] Si anodes have been extensively studied, including both their electrochemical performance advantages and operational limitations, and the knowledge gained could serve as a foundation to further understand other types of anode electrodes undergo similar alloying process.

Studies have revealed that during the initial charge, amorphous phase growth in the crystalline Si leads to the formation of sharp interfaces (≈1 nm thick).[[qv: 94a]] Investigation into the atomistic mechanism of dynamic lithiation using in situ transmission electron microscopy (TEM) has revealed that the lithiation kinetics are anisotropic and controlled by the migration of the interface.^96^ Very recently, real‐time analyses of silicon nanomaterials to elucidate the changes in the dimensions and diffraction intensity indicate that the initial lithiation of Si nanoparticles results in anisotropic volume expansion favoring the (110) direction owing to the smaller Li diffusion energy barrier at the Si‐electrolyte interface along this direction.^97^ In other words, a buildup of Li near the surface of the Si caused by the sluggish transport of lithiated Si upon electrochemical alloying of Si with Li will cause large stress and volume changes. Such stress and volume changes further cause surface fracture and limit capacity by inducing a large polarization.

Several strategies have been experimentally evidenced to solve these problems:[[qv: 95c,98]] 1) nanostructured particles such as nanowires,[[qv: 95b,98a,99]] thin films,[[qv: 95a,100]] and mesoporous structures^101^ have been suggested to better accommodate large (de)lithiation strain without fracture; 2) combining Si nanoparticles with a carbon matrix to suppress local mechanical stress and avoid the formation of two‐phase regions to prevent large volume expansion during the cycling process.^97^, ^102^ Various silicon–carbon composites have been used to enhance the electrochemical performance of silicon anodes. Moreover, the properties of Si–C composites have been found to be highly dependent on the precursors used. As for nanosized silicon, its cycle life is still limited owing to the unstable solid‐electrolyte interphase (SEI) on the surface and high specific surface area. Various studies have demonstrated that the SEI is very dynamic, with a composition that is greatly dependent on cycle life and voltage,[[qv: 8b]] and thickness changes during cycling, becoming thinner during delithiation and thicker during lithiation.^103^ Silicon electrodes with a secondary structure inspired by pomegranate fruit^103^ have been successfully fabricated to control SEI formation because it contributes to low Coulombic efficiency.

These results indicate that the combination of composite structure and geometry to overcome the challenges confronting silicon anode electrodes still needs further investigation to meet high energy density requirements.

#### 3.4.2 Sn

Research on Sn has been focused on tin‐based oxides, which has shown significantly improved cyclic performance compared with that of stand‐alone Sn electrodes.^104^ Sn forms cubic phase Li_22_Sn_5_ in the highest lithiation, leading to a theoretical capacity of 990 mAh g^−1^, which was later clearly identified by Mössbauer spectroscopy.^105^ Thus, the electrochemical performance of Sn for Li‐ion batteries is dependent on the voltage cut‐offs because the Sn lithiation process follows the formation of intermediate lithiated crystalline phases:

(11) $$\begin{array}{l} \mathrm{Sn}\to {\mathrm{Li}}_{2}{\mathrm{Sn}}_{5}\left(0.69\;V\right)\to \mathrm{LiSn}\left(0.57\;V\right)\to {\mathrm{Li}}_{7}{\mathrm{Sn}}_{3}\left(0.4\;V\right)\\ \;\to {\mathrm{Li}}_{5}{\mathrm{Sn}}_{2}\to {\mathrm{Li}}_{13}{\mathrm{Sn}}_{5}\to {\mathrm{Li}}_{7}{\mathrm{Sn}}_{2}\to {\mathrm{Li}}_{17}{\mathrm{Sn}}_{4} \end{array}$$

**Figure 10:**
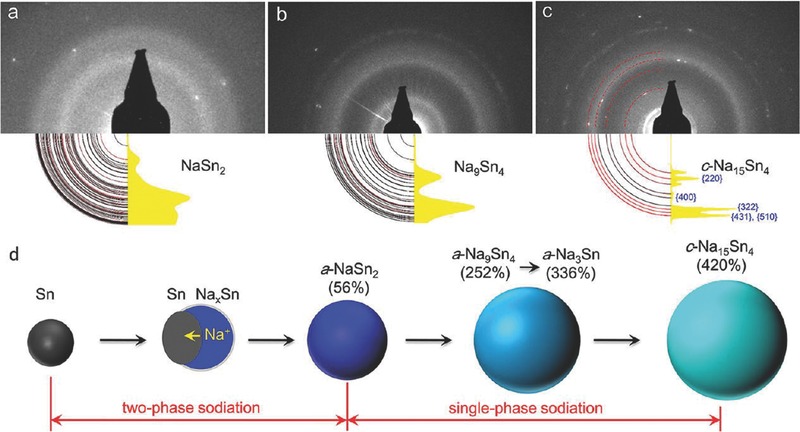
Three a‐Na_x_Sn phases in the single‐phase sodiation. a) EDP (electron diffraction pattern) of the first α‐Na*_x_*Sn phase. b) EDP of the second a‐Na*_x_*Sn phase. c) EDP of the third α‐Na*_x_*Sn phase, d) Schematic illustration of the structural evolution of Sn NPs during the sodiation. Reproduced with permission.^107^ Copyright 2012, American Chemical Society.

As an anode electrode, Sn is expected to react with sodium to form fully sodiated crystalline Na_15_Sn_4_, delivering a theoretical capacity of 847 mAh g^−1^.^106^ Furthermore, the redox potentials for the formation of Na‐Sn alloys are a few hundred mV lower than those of Li–Sn alloys.[[qv: 106b]] In situ TEM analysis has also confirmed that Sn nanoparticles are expanded by about 420% after full sodiation without the observation of structural cracking or fracture (see in **Figure** **10**).^107^ The EDP (electron diffraction pattern) was taken when the reaction front had just swept the entire Sn nanoparticles (NPs). The simulated EDP indicated that the composition of the first α‐Na*_x_*Sn phase was close to that of the NaSn_2_ phase. The third α‐Na*_x_*Sn phase was identified a‐Na_3_Sn based on volumetric expansion measurements.

#### 3.4.3 Ge and Sb

Germanium is an extensively studied anode material owing to its superior intrinsic electrical conductivity (10^4^ times higher than that of silicon) and fast lithium diffusion (400 times faster than in Si at room temperature).[[qv: 8b,108]] These advantages ensure a high rate capability and efficient charge transport, which offsets the effect of the relatively large atomic size of Ge. The Ge lithiation process ultimately forms Li_15_Ge_4_,^109^ leading to a high lithium storage capability of 1600 mAh g^−1^, and the delithiation process results in the formation of a porous amorphous phase.^110^ During the alloying process, the change in properties would be continuous from Si to Sn and then to Ge. J. Nelson Weker et al. reported the first observation of the complete macroscopic fracturing of Ge anodes in lithium‐ion batteries, thus elucidating the failure mechanism of alloying anode materials.^111^ Their results demonstrated that small particles experience volume change and cracking before their larger counterparts during lithiation. Ge shows electrochemically reversible sodiation/desodiation with Na, assuming the formation of NaGe at about 0.4 V versus Na^+^/Na with a theoretical capacity of 369 mAh g^−1^,^112^ and small feature sizes are critical for rapid, reversible electrochemical sodiation of Ge.

As for the study of Sb, a large amount of research has been published despite its high voltage plateau at over 0.8 V vs lithium. The lithiation process

(12) $$\mathrm{Sb}\to {\mathrm{Li}}_{2}\mathrm{Sb}\to {\mathrm{Li}}_{3}\mathrm{Sb}$$

forms intermediate Li_2_Sb and cubic Li_3_Sb phases at room temperature, which correspond to two plateaus in the voltage curve at 0.956 and 0.948 V.[[qv: 8b,113]] During delithiation, Li_3_Sb is directly converted to Sb. Note that Li_2_Sb (18 atoms per unit cell) does not form during delithiation owing to its high melting point of 825 °C. Recently, many research groups have demonstrated that Sb based Na‐ion batteries show very stable capacity retention over 100 cycles and a high reversible capacity of about 500–600 mAh g^−1^, with current peaks observed at 0.77 and 0.90 V vs Na^+^/Na.^114^ Sb reversibly alloyed with Na, transforming into hexagonal Na_3_Sb despite the bulk Sb particles used, and showed better cycle performance in Na‐ion batteries than in Li‐ion batteries.[[qv: 113c]]

(13) $$\mathrm{Sb}\to {\mathrm{Na}}_{x}\mathrm{Sb}\to {\mathrm{Na}}_{3}\mathrm{Sb}\;$$

The volume changes of Sb to Li_3_Sb and Na_3_Sb are about 135% and 293%, respectively.^115^ The better cycle performance of Na‐ion batteries can attributed to reduced anisotropic mechanical stress because of its repeated formation of only one crystalline phase, Na_3_Sb (during desodiation, hexagonal Na_3_Sb converts directly to amorphous Sb). In Sb based Li‐ion batteries, the Sb electrodes suffer from detrimental volume changes during cycling, which degrades the electrochemical performance.

Alloy binary compounds have been explored to improve electrochemical performance. Active‐inactive and active‐active binary alloys are two types of binary compounds widely investigated. Active elements can be alloyed with inactive elements to alter electrochemical performance, grain structure, and reduce alloy volume change during electrochemical processes.[[qv: 8b]] Addition of an active phase can lead to higher energy density, and even take part in lithiation reactions in some examples. Alloys of two active elements possess characteristics different from those of single‐active materials. Here, we only address Sn‐based alloy examples, because different alloy combinations are complex. Sn‐Sb alloys have attracted great attention owing to their superior electrochemical performance as an active/active binary compound. SnSb alloys undergo a two‐step reaction as shown in Equations (14) and:

(14) $$\mathrm{SnSb}+3A+3e^{-}\to A_{3}\mathrm{Sb}+\mathrm{Sn}\;\left(A=\mathrm{Li},\mathrm{Na}\right)$$

(15) $$\begin{array}{l} \mathrm{Sn}+{\mathrm{Na}}^{+}+e^{-}\to {\mathrm{Na}}_{3.75}\;\mathrm{Sn}\\ \mathrm{or}\;\mathrm{Sn}+{\mathrm{Li}}^{+}+e^{-}\to {\mathrm{Li}}_{4.4}\;\mathrm{Sn} \end{array}$$

SnSb lithiation resulting in a theoretical capacity of 825 mAh g^−1^. In the first step, the conversion reaction occurs first and forms Li_3_Sb rearranged in Sn domains, which buffer the volume expansion of the Sn phase. Li–Sn alloy forms in the subsequent step (Equation (15)). Both active elements in this system are electrochemically active with Li (and Na), and the presence of Sb improves lithiation kinetics. Compared with the simple metals, SnSb/C shows better capacity retention and good rate capability, and the electrochemical behavior of the SnSb is different from the simple metals.[[qv: 9b]]

#### 3.4.4 P

Phosphorus is another attractive anode material that has shown promising electrochemical performance for both lithium‐ion batteries^116^ and sodium‐ion batteries.^117^ P is a Group V nonmetallic element in the periodic table and has three allotropes known as white, red, and black phosphorus.[[qv: 116b]] White phosphorus begins to burn at 30°C, so it is not a suitable electrode material in terms of safety. Red P and black P have been studied as anode materials because of their chemical stability at room temperature and atmospheric pressure.

P can take up three Li or Na atoms to form Li_3_P^118^ and Na_3_P[[qv: 117b,119]] respectively, according to the following Equation

(16) $$P+3A+3e^{-}↔A_{3}P\;\left(A\;\;=\;\;\mathrm{Li},\;\mathrm{Na}\right)$$

This three‐electron‐transfer reaction of P provides an extremely high theoretical specific capacity of 2596 mAh g^−1^, the highest among all known anode materials for both LIBs and NIBs. The reversible lithiation peaks for phosphorus appear in the 0.6–0.9 V voltage range,[[qv: 116b,118a,120]] a little higher compared with other anodes, while the sodiation peaks for phosphorus appear in the 0.5–0.0 V range,[[qv: 117a,121]] which is suitable for a Na‐ion battery anode. Therefore, phosphorus‐based materials are very promising anodes for secondary batteries because of their high reversible capacity and high energy density.

Black phosphorus is the most thermodynamically stable allotrope of P, and has a high bulk electrical conductivity (≈10^2^ S m^–1^), which are beneficial for realizing both high power and energy density.[[qv: 9b,122]] Black P can be synthesized from commercially available amorphous red P through a simple high energy mechanical milling technique at high temperature and pressure. This simple transformation method is expected to be widely applicable in the electronics industry, owing to the semiconducting nature of black P. It has a similar structure to graphite, with a layered structure of puckered sheets. Graphite is comprised of stacks of six membered carbon rings located in the same plane and has an interlayer distance of 3.35 Å. In the case of black P, each phosphorus atoms is bonded with two other atoms lying on the same plane and with one phosphorus atom in a different plane. This creates a network of six‐membered P_6_ rings that further forms a flaky layered material in which the interplanar distance is 3.09 Å.[[qv: 116b,123]] However, black P does not undergo the intercalation reaction characteristic to graphite. The uptake of lithium ions or sodium ions by black P to the final compositions of Li_3_P and Na_3_P, respectively, occurs with the breaking of P–P bonds, which is different to the intercalation reaction in graphite, where the lithium ions are stored between the graphene layers without inducing any C–C bond breakage.

**Figure 11:**
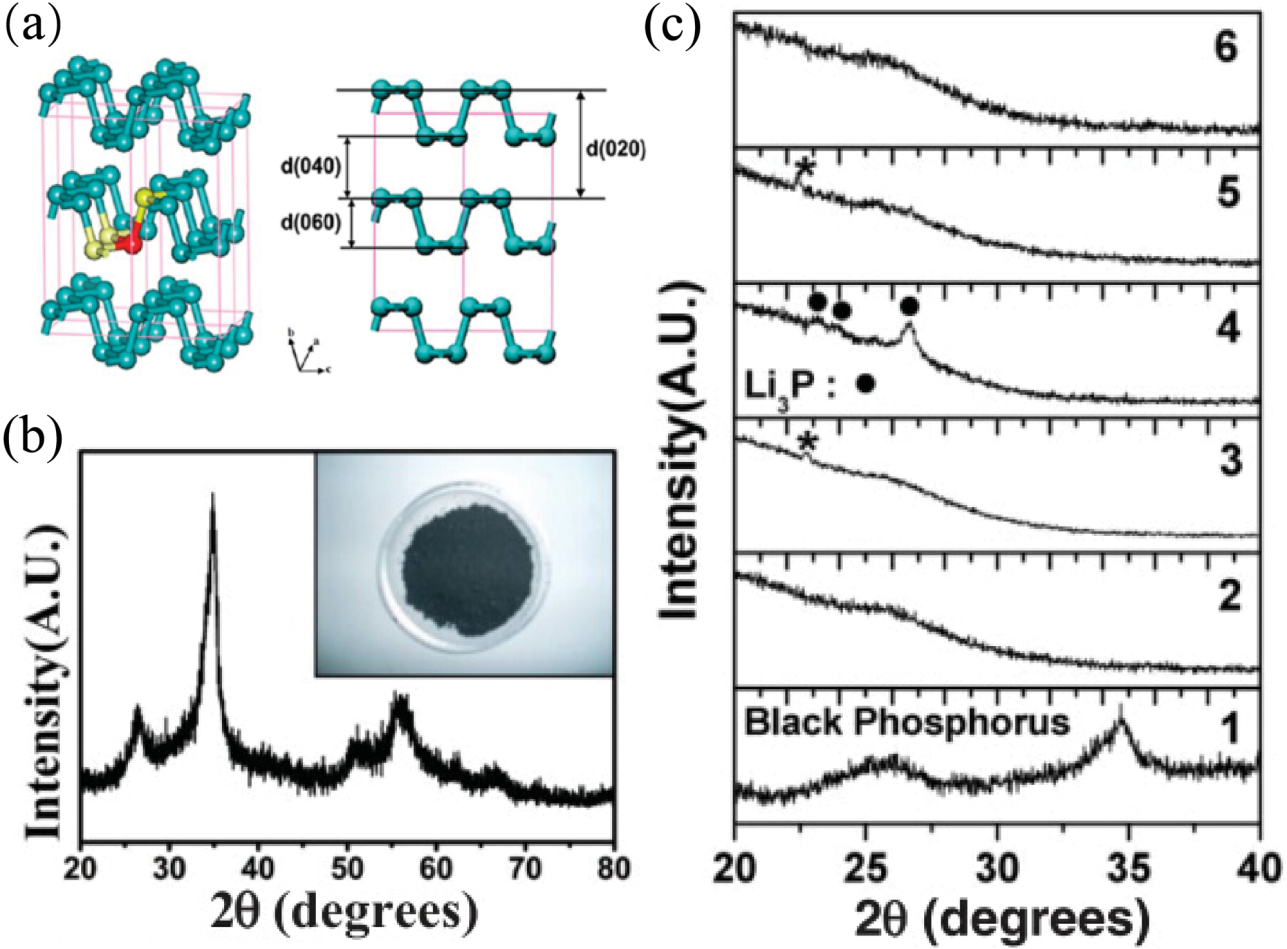
a) Crystal structure, b) color photo image, and c) X‐ray diffraction patterns of black P. a) Reproduced with permission.^122^ 2012, J. Phys. Chem. C b,c) Reproduced with permission.[[qv: 116b]]

Black P was first used as an anode material for lithium‐ion batteries in 2007, prepared by the high‐energy mechanical milling method.[[qv: 116b]] The obtained black P–carbon composites (crystal structure and color photographic images are shown in **Figure** **11**a,b) showed a charge capacity of 1279 mAh g^−1^ as an anode material. However, its capacity decreased drastically in the voltage range of 0.0–2.0 V owing to the large volume change arising from the formation of the Li_3_P phase (confirmed by the X‐ray diffraction (XRD) pattern shown in Figure 11c). Careful control of the voltage range to between 0.78 and 2.0 V led to excellent cycle performance and a capacity of more than 600 mAh g^−1^ over 100 cycles. The reactions involved in this voltage range followed the order Black P → Li_x_P → LiP, corresponding to one electron transfer. Cui's group also successfully demonstrated high‐performance black phosphorus–graphite composite anodes for lithium‐ion batteries prepared by mechanochemical reaction through high energy mechanical milling.[[qv: 118a]] Robust P–C bonds have been generated between black P and a variety of carbon materials. The choice of carbon structure was found to play an essential role in the formation of stable P–C bonds, with graphite the best, retaining a reversible capacity of 1840 mAh g^−1^ after 100 cycles. Very recently, the discovery of a new 2D compound, monolayer black P, has attracted a great deal of attention.^124^ Monolayer black P is, besides graphene, the only stable 2D elemental material that can be mechanically exfoliated.^125^ This few‐layer black P not only has a large surface‐volume ratio, but also a puckered surface. Therefore, few‐layer black P may be able to serve as a high‐capacity host of Li and Na in secondary batteries.^126^

Red P is amorphous and abundant, but shows poor experimental performance owing to its electrically insulating properties. Chou's group^119^ prepared a P/carbon nanotube (CNT) composite simply by hand grinding commercial red phosphorus with CNTs, and unexpectedly achieved a high reversible capacity of 2210 mAh g^−1^; however, only 76.6 % of the initial capacity was delivered after 10 cycles. The network structure of the CNTs effectively buffered the enormous stress arising from the volume expansion of the P particles, and provided pathways for electron transport.

However, the battery performance of these materials is still unsatisfactory, particularly in regards to fast capacity fading and low Coulombic efficiency. The main reason for this is the large volume change (volume change between P and Na_3_P of 491%, electrode thickness increase of 187%, and volume change during lithiation of over 300%), which causes poor electrical contact between phosphorus particles and the conducting matrix, pulverization of the phosphorus particles, and continuous growth of the solid electrolyte interphase. A novel phosphorus/graphene nanosheet (P/G) hybrid nanostructures anode was developed through the ball‐milling of graphene stacks and red phosphorus.[[qv: 117a]] The graphene attached to the P particles via chemical bonding behaved as a conductive matrix that maintained electrical contact with the phosphorus during the volume change. This strategy dramatically improved the electrochemical performance of the P, with a reported specific capacity of 2077 mAh g^−1^ and enhanced retention of 95% after 60 cycles.

Moreover, various transition metal phosphides have been introduced as anodes for LIBs and NIBs.^127^ Take Co_3_P as example, redox behavior is based on shuttling between two different phosphorus oxidation states, and the lithiation/delithiation mechanism in the first cycle is as given in Equation:^128^

(17) $${\mathrm{CoP}}_{3}+9{\mathrm{Li}}^{+}+9e^{-}\to 3{\mathrm{Li}}_{3}P+\mathrm{Co}$$

This process is comparable to the behavior for SnSb as mentioned above, which has been proposed to transform into LiSb and Sn on Li uptake.[[qv: 8b]] Further, XPS spectrum confirms the existence of LiP during subsequent cycling, and the simplified mechanism can be written as follows:

(18) $$3{\mathrm{Li}}_{3}P↔3\mathrm{LiP}+6{\mathrm{Li}}^{+}+6e^{-}$$

In this system, initial uptake of Li forms highly dispersed cobalt clusters embedded in a matrix of Li_3_P; extraction of Li from this ion‐conductive matrix on charge yields nano‐particles of LiP, with little change evident in the oxidation state of the Co site.

In conclusion, P anodes undergo volume expansion owing to the destruction of their structure by alkali metals such as Li and Na. Current research is essentially focused on controlling the unfavorable effects of volume changes during cycling and enhancing the electronic conductivity of the composite electrode. There are other tough challenges confronting the development of phosphorus based electrodes. Moreover, the safety issues of phosphorus should also be considered; as is well known, alkali metals and phosphorus can react to form phosphine, which is highly flammable and toxic.

Alloying anodes are promising electrode materials exhibiting high energy density because of their ability to uptake more than one Li atom. Si has especially been studied owing to its favorable properties. P and Ge also show high specific capacity and good rate capability. Other metals such as Sn possess some of the desired characteristics but also face challenges such large polarization and pulverization. However, these alloying anodes always exhibit rapidly decaying specific capacity during continuous cycling owing to their dramatic volume expansion and contraction during lithiation and sodiation. As for Li‐based alloys, Li atoms occupy a volume of 14.8 Å Li^–1^ and 8.9 mL mol^–1^. Na atoms have been found to occupy a volume of 30.3 Å Na^–1^, corresponding to 18.2 mL mol^–1^, during sodiation in a series of Na–Me alloy systems. These results demonstrate that the volume change is essentially independent of Me (Si, Ge, Sn and Pb) and the level of lithiation and sodiation.^115^ Such huge volume expansion is detrimental to the electrodes; the resulting fracture of the active material into fragments causes loss of electrical contact. The mechanisms of fracture failure were first investigated using transmission X‐ray microscopy with Ge anodes in lithium‐ion batteries.^111^ Preventing the fracture of anode materials during electrochemical processes is essential to utilize their high specific capacity for long‐term cycling, high energy density secondary batteries. Various approaches have been employed to suppress the volume change of alloying anodes materials. Nanosizing (nanoparticles, nanowires, nanotubes) is the most extensively studied method owing to the significant relationship between the physical and electrochemical performance of the active materials. Other methods used include creating hierarchical structures that combine the advantages of both nanomaterials and bulk materials, and dispersing active alloy materials into conductive matrices to form composite structures.

### 3.5 Conversion Reactions

#### 3.5.1 Metal fluoride‐based cathode materials

The growing demand for electric vehicles and electronic devices has heightened research enthusiasm for high performance positive materials. If we want to obtain a breakthrough in cathode materials, complete utilization of high oxidation state transition metals should be key. The reversible electrochemical conversion reaction of high valence metal compounds can be described as follows:^129^

(19) $$mnA^{+}+M_{n}X_{m}+mne^{-}↔mA_{n}X\;+nX^{0}$$

In which A represents the alkali metal ions (A = Li^+^, Na^+^, K^+^, etc.), M represents the transition metal ions (M = Fe^3+^, Cu^2+^, Ni^2+^, etc.), and X denotes certain anions (X = F^−^, O^2−^, S^2−^, etc.). In particular, owing to their transfer of three electrons during the reaction process, metal fluoride‐based cathodes meet the target of light weight and multi electron transferring electrodes.^130^ Because various metals can form compounds with fluoride ions, many kinds of metal fluoride‐based cathodes have been investigated and found to deliver different electrochemical performances in batteries.^131^ As shown in **Table** **1**, these metal fluorides universally exhibit higher operating voltages and capacities than those of metal oxides and sulfides. The reason for this performance improvement is mainly attributed to the electronegativity of F^−^ ions relative to the covalent characteristics of the Me–O and Me–S bonds.

**Table 1:** A comparison of the theoretical cell voltage (E^0^) and Li storage capacity of various metal fluorides.[[qv: 130a]]

	E^0^/V	Capacity/mAh g^−1^		E^0^/V	Capacity/mAh g^−1^
**CoF_3_**	3.617	694	FeF_3_	2.742	712
**CuF_2_**	3.553	528	VF_3_	1.863	745
**NiF_2_**	2.964	554	TiF_3_	1.396	767

In fact, metal fluoride‐based cathodes were originally used for the primary batteries reported by Amatucci and co‐workers.^132^ Deep investigation into the reaction mechanism of metal fluorides revealed that some metal fluorides (FeF_3_, VF_3_, TiF_3_, etc.) can be used in secondary cells.^133^ However, these batteries only delivered reversible capacities of less than 100 mAh g^−1^, far below their theoretical capacity.

**Figure 12:**
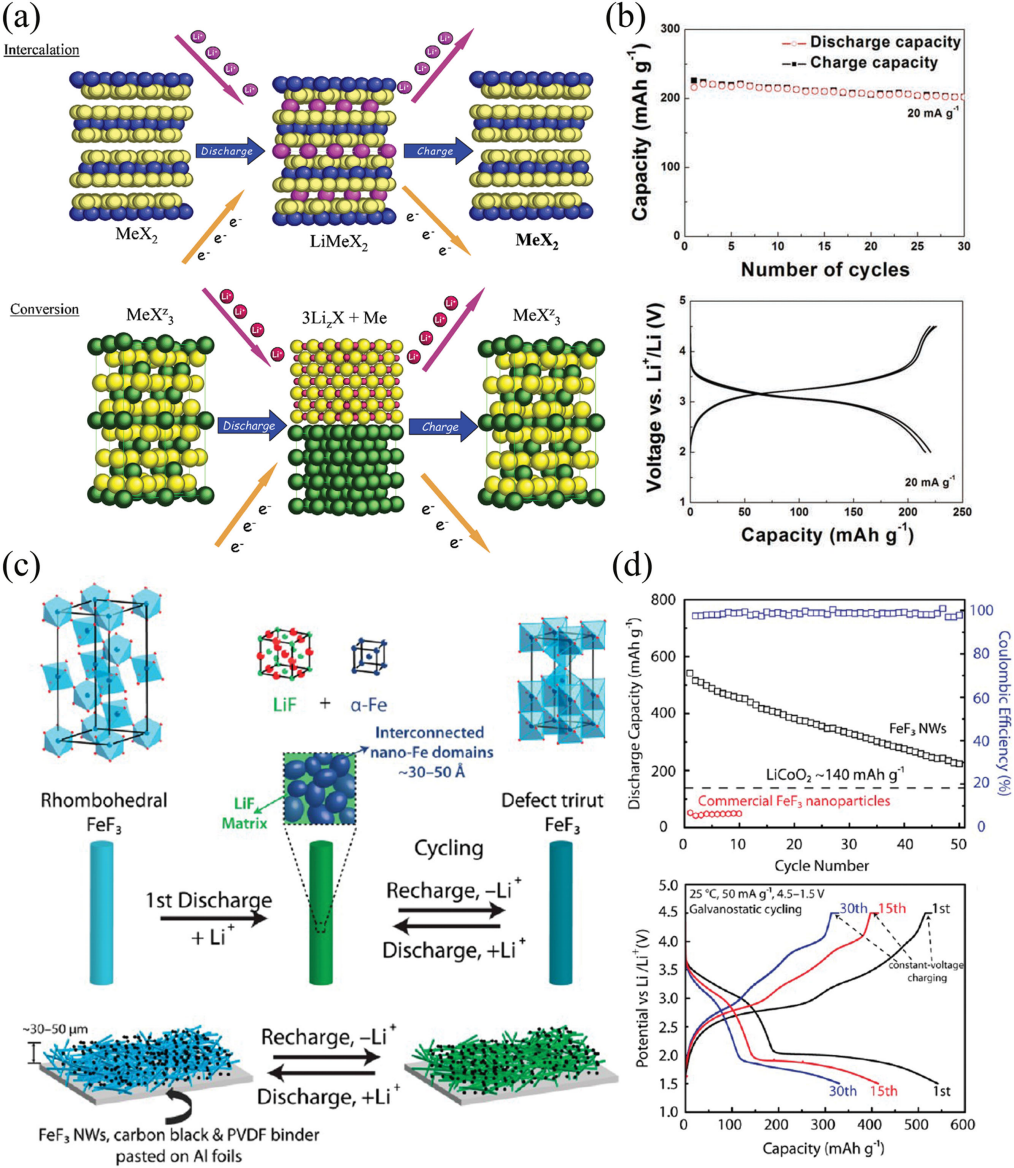
a) Schematics of reaction mechanisms based on intercalation and reversible conversion. b) The electrochemical properties corresponding to the above mechanism. c) Schematics of conversion mechanism with phase transformation at first cycle. d) The electrochemical properties corresponding to the above mechanism. a) Reproduced with permission.[[qv: 130a]] Copyright 2007, Elsevier. b) Reproduced with permission.^211^ Copyright 2014, Royal Society of Chemistry. Copyright 2014, American Chemical Society. c) Reproduced with permission.^138^ Copyright 2012, American Chemical Society.

Multi‐electron transfer is not a one‐step process, which can be divided into multiple steps that involve different reaction mechanisms (**Figure** **12**a).[[qv: 130a]] For instance, metal trifluorides exhibit a two‐step reaction process: First, one lithium ion inserts into the interlayer of FeF_3_ through one electron transfer, which is an intercalation mechanism like that of conventional transition metal oxide positive materials; secondly, the LiFeF_3_ product of the first step and two further lithium ions are transformed into LiF and Fe, which is a conversion mechanism. Researchers have designed batteries with high capacity and cycling stability based on this series of reactions. Fichtner and co‐workers^134^ synthesized a carbon–iron lithium fluoride nanocomposite which exhibited an initial discharge capacity of 324 mAh g^−1^ that remained at 270 mAh g^−1^ after 200 cycles. Their experiment proved that mixing FeF_3_ with carbon material can effectively improve the performance of FeF_3_. Subsequently, FeF_3_ nanoflowers on CNT branches obtained by a self‐assembly process were reported to display a high specific capacity of 210 mAh g^−1^ based on the conversion reaction process and excellent rate performance of about 150 mAh g^−1^ even at 500 mA g^−1^ current density (Figure 12b).^135^

Amatucci et al. proposed that the size of metal fluoride particles might influence their conversion efficiency.^136^ To enhance their conversion rate, the particle size of fluorides should be limited to the nanoscale. A complex porous FeF_3_ nanowire structure reported by Li et al. showed an outstanding initial capacity of 500 mAh g^−1^. A porous FeF_3_ nanosphere structure also exhibited a high initial capacity of more than 220 mAh g^−1^.^137^ These experimental results are in accord with the explanation above. Recently, Jin and co‐workers introduced a FeF_3_ nanowire structure that yielded a discharge capacity of as high as 543 mAh g^−1^ and remained 223 mAh g^−1^ after 50 cycles, and revealed a dimensional effect on the conversion process.^138^ As depicted in Figure 12c, the initial FeF_3_ particles with rhombohedral structure transform into a mixture of LiF and α‐Fe during the first discharge process. Subsequently, this mixture and the remaining FeF_3_ particles, which contain defect trirutile, undergo reciprocal transformation. This phase evolution during the electrochemical conversion process primarily depends on the contact area between the Fe particles and LiF particles. Therefore, the size of the FeF_3_ particles is crucial to their electrochemical performance. The phase transformation in the first cycle and reversible conversion reaction in subsequent cycles led to the high initial capacity and good stability after 50 cycles shown in Figure 12d. However, knowledge of the fundamentals of the conversion mechanism of FeF_3_ is still lacking. In addition to cathodes for Li‐ion batteries, FeF_3_ also can be used in positive electrodes for Na‐ion batteries. The sheet‐like FeF_3_/graphene composite reported by Wang et al. showed a high initial discharge capacity similar to the performance measured in Li‐ion batteries.^139^

Some other metal fluorides have been used as cathodes for secondary cells or doped into FeF_3_. For example, a Co‐doped FeF_3_/C nanocomposite cathode offered a new way to improve the performance of FeF_3_ through cationic doping.^140^ The cathode showed a discharge capacity of 151.7 mAh g^−1^ at 1C, and 92% of its initial capacity after 100 cycles. Both CuF_2_ 141 and MoF_3_ 142 materials have been validated as suitable for cathode use. These studies have provided the basis for future investigations into positive materials exhibiting multi‐electron transfer.

#### 3.5.2 Metal oxides

Transition metal oxides, including binary and mixed oxides, have been the most widely investigated high‐performance anode materials for both LIBs and NIBs in recent years because of their good safety, environmental friendliness, abundance, and notably high specific capacity achieved by conversion reactions.[[qv: 13a,143]] Taking Co_3_O_4_ (Co^II^Co^III^_2_O_4_) as an example, full reduction of the Co^2+^/Co^3+^ to form Co and Li_2_O leads to a capacity of 890.4 mAh g^−1^. In comparison, reducing Co^4+^ to Co^3+^ in the classical LiCoO_2_ intercalation compound leads to a capacity of 273.8 mAh g^−1^ (corresponding to about 0.5 lithium atoms per formula reversibly intercalated).^144^

With the rapid development of materials design strategies and synthesis techniques, great progress has been achieved in the fabrication of transition metal oxides with various nanostructures and enhanced electrochemical performance.[[qv: 1c,3a,8c,145]] As anode materials for LIBs and NIBs, metal oxides typically store Li^+^ and Na^+^ via a redox conversion reaction, which results in the reversible formation of metallic nanoparticles dispersed intimately within the Li_2_O matrix that maintain electronic conductivity.[[qv: 3c,8c,8e,143d,146]] However, it is well known that metal oxides go through an electrochemically induced volume expansion upon the formation of Li_2_O during initial charging.^147^

Binary transition metal oxides (MO*_x_*, M = Ni, Fe, Mn, Co, etc.), in which M may have a variety of chemical valences, react with alkali ions (Li^+^ and Na^+^) via a multi‐electron conversion process, as given in Equation .

(20) $${\mathrm{MO}}_{x}+2xA^{+}+2xe^{-}↔xA_{2}O+M\;\left(A\;\;=\;\;\mathrm{Li},\;\mathrm{Na}\right)$$

Because of the multi‐electron redox reactions involved, these materials often exhibit high specific capacity. In spite of this, however, most metal oxides have exhibited relatively lower reversible capacities of less than 400 mAh g^−1^, and suffer from similar issues to those observed for alloying electrodes.[[qv: 8a]] Among the metal oxides used as anode materials for LIBs and NIBs, Fe, Mn, and Ni‐based oxides have been extensively investigated. Taking iron‐based oxides as an example, Fe_2_O_3_ and Fe_3_O_4_ have drawn particular attention because of their high theoretical capacity of ≈1000 mAh g^−1^, non‐toxicity, high abundance, and low cost.[[qv: 8c,148]] The forward conversion reaction of Fe_2_O_3_ with Li^+^ is thermodynamically feasible and the formation of Fe° from Fe^3+^ involves the transfer of multiple electrons per metal atom. In comparison, Fe_3_O_4_ delivers about 900 and 400 mAh g^−1^ in Li‐ion and Na‐ion batteries, respectively.^149^ However, metal oxides always suffer from low conductivity and drastic volume expansion, which cause performance degradation. Strategies such as nanoscaling, structural modification, and the introduction of conductive material have been proposed as ways to facilitate the reverse extraction of Li^+^ from the Li_2_O, mitigate the pulverization, and further enhance the structural stability of these electrode materials.[[qv: 1b,1c,3c,143d]]

### 3.6 Conversion and alloying reactions

In the second type of reaction, the M in MO_*x*_ is an electrochemically active element such as Zn, Ge, Sn, Sb, In, and Pb. Most metal oxides with active metal elements deliver high reversible capacity. An alloy is formed by the reaction between a metal oxide and lithium or sodium and proceeds in two distinct steps, as shown in Equations and below:[[qv: 1c,13a]]

(21) $${\mathrm{MO}}_{x}+2xA^{+}+2xe^{-}↔xA_{2}O+M\;\left(A\;=\;\;\mathrm{Li},\;\;\mathrm{Na}\right)$$

(22) $$M+yA^{+}+ye^{-}↔A_{y}M\;\left(A\;\;=\;\mathrm{Li},\;\mathrm{Na}\right)$$

First, the metal oxide is reduced to the elemental metal by a displacement reaction, which, depending on the metal oxide, may be irreversible or poorly reversible owing to the poor conductivity of the metal oxide. As a result, the practical capacity of this group of oxides is based on the alloying reaction between the metal and lithium, which is the second step of the mechanism. The alloying and de‐alloying of the metals listed above with lithium is typically accompanied by a large change in the volume of the electrode. Repeated expansion and contraction has been shown to cause cracking and eventual disintegration of such electrodes on extended cycling, which results in poor cycling performance.

SnO_2_ has long been regarded as a promising anode material candidate for LIBs and NIBs owing to its high theoretical capacity (782 mAh g^−1^ for LIBs and 667 mAh g^−1^ for NIBs), low cost, low toxicity, and natural abundance.^150^ Moreover, this material is representative of a special category of metal oxides that exhibit solid state amorphization upon initial charging.

The results of in situ observation, using a nanoscale electrochemical device inside a transmission electron microscope, of the lithiation of single SnO_2_ nanowires during electrochemical charging have suggested that the following electrochemical reactions occur:^147^

(23) $$\;{\mathrm{SnO}}_{2}+4{\mathrm{Li}}^{+}+4e^{-}\to 2{\mathrm{Li}}_{2}O+\mathrm{Sn}$$

(24) $$\mathrm{Sn}+x{\mathrm{Li}}^{+}+xe^{-}\to {\mathrm{Li}}_{x}\mathrm{Sn}\;\left(0\leq x\leq 4.4\right)$$

**Figure 13:**
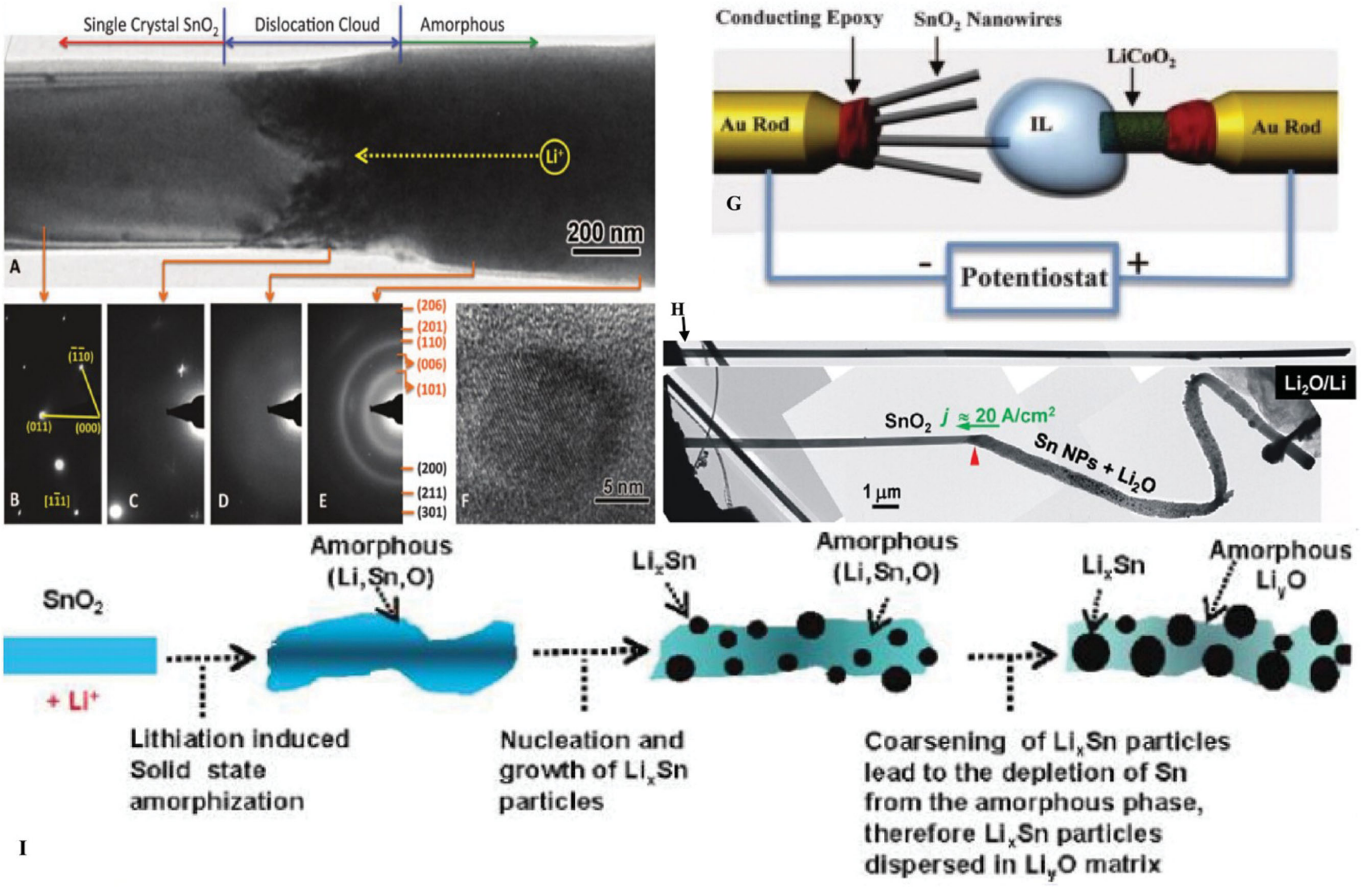
A)TEM image of the nanowire contain different sections during lithiation. B–E) EDPs (electron diffraction pattern) from the different sections of the nanowire. F) An HRTEM (high resolution transmission electron microscopy) image from a charged nanowire showing Sn nanoparticles dispersed in an amorphous matrix. G) Schematic drawing of the experimental assembly of nanoscale device inside a transmission electron microscope.(H) High‐magnification images showing the morphology change of the nanowire during lithiation. (I) Schematic drawing showing the structure and phase evolution of the SnO_2_ upon initial charging. A–G) Reproduced with permission.^147^ Copyright 2010, American Association for the Advancement of Science. H) Reproduced with permission.^154^ Copyright 2012, Elsevier. I) Reproduced with permission.^151^ Copyright 2011, American Chemical Society

Upon initial charging to ca. 0.9 V vs Li^+^/Li, the SnO_2_ is transformed to Li_2_O and Sn.^151^ This conversion reaction is irreversible, and the formation of Li_2_O causes instant volume expansion. The subsequent charging and discharging are related to the second reversible alloying reaction between Li*_x_*Sn and Sn (24); amorphous Li_2_O does not participate in the electrochemical reaction. The in situ experiments described above confirmed that the nanowire consisted of nanocrystalline Li*_x_*Sn and Sn dispersed in an amorphous Li_2_O matrix. The nanowire underwent dynamic structural evolution after charging, as can be seen in **Figure** **13**A–E and H.^147^, ^151^ Further, detailed observation of the microstructural evolution of electrodes has provided direct evidence regarding the spatial relationship between newly formed Li_2_O and Li*_x_*Sn.^151^ The structural evolution of SnO_2_ upon initial charging is summarized by the schematic drawing in Figure 13I. The second step may not occur if the current passing through the nanowire is large, especially for bulk electrodes in real batteries. In particular, the Li cycling performances of Sn‐based oxides were found to be superior in comparison to Sn, due to the presence of electrochemically formed amorphous Li_2_O.^152^ The Li_2_O behaves as buffering domain during Li–Sn alloy formation and decomposition, thus the dispersion of nano‐Sn in amorphous Li_2_O can maintain the structural integrity of the anode. It also provides ionically conducting medium for the Li ion migration and helps to keep the electrochemically formed nano‐Sn metal particles apart and prevents their agglomeration.^153^ However, the current density across the electrode may not be homogeneous, and such inhomogeneous current density may cause abnormal and hazardous Sn precipitation during the lithiation process.^154^ Besides volume expansion, the growth of Sn particles on the anode is another cause of large irreversible capacity fading in SnO_2_ based electrodes. Sn precipitation causes loss of contact of the Sn particles, possible short‐circuit hazard, and Sn‐catalyzed electrolyte decomposition.^154^, ^155^ A thin coating of Sn on SnO_2_ electrodes is an effective strategy to prevent Sn further growth that may short the battery, by changing the kinetics of Sn precipitation. A number of composite electrodes have been developed and extensively studied in an attempt to obtain a more homogenous current density for better electrochemical performance.^154^

The theoretical reversible lithium storage capacity for the second reaction is readily calculated to be 790 mAh g^−1^. Further, Wang and co‐workers showed that octahedral SnO_2_ nanoparticles (≈60 nm) obtained using a hydrothermal method delivered a reversible capacity of about 500 mAh g^−1^ with stable cycle performance as an anode for sodium‐ion batteries. The improved cyclability was attributed to the retardation of Sn aggregation during cycling by the Na_2_O matrix. However, the actual deployment of its bulk counterpart is restricted by some intrinsic drawbacks: (1) The huge volume change upon alloying and de‐alloying (≈300 %) leads to pulverization of the electrode materials and loss of electrical contact with binders and current collectors. (2) A poor electrical conductivity at room temperature further restricts the electrode kinetics at high rates and comprises the cyclability of the electrode.[[qv: 146d,150d]]

Nanostructuring is therefore necessary to relieve the stress at the electrode surface and provide necessary void space for expansion.^147^ Nanostructured SnO_2_/carbon composites have been extensively studied. These composites not only accommodate the volume change and protect the SnO_2_ from aggregation and pulverization, but also enhance the electronic conductivity of overall electrode.^147^, ^156^ For example, an amorphous carbon‐coated SnO_2_‐electrodeposited porous carbon nanofiber (PCNF@SnO_2_@C) composite was introduced as a sodium‐ion battery anode material.[[qv: 146d]] Electrochemical performance studies revealed that the as‐prepared composite anode possessed high‐capacity (374 mA g^−1^), good capacity retention (82.7%), and large Coulombic efficiency (98.9% after the 100th cycle). A free‐standing composite of carbon foam encapsulated SnO_2_ nanocrystallites with tunable size range was developed as an anode for LIBs, in which the carbon foam served as a build‐in hierarchical porous current collector to enhance the electrical conductivity of the anode.^157^ This composite exhibited good capacity retention (93.6% after 100 cycles at 500 mA g^−1^), impressive rate capability of up to 8 A g^−1^, and long cycle life of 250 cycles. This good cycling performance was attributed to the synergistic effect of the electrically conductive network, percolating macro‐pores, large surface area, and SnO_2_ nanocrystallites with tunable size range. These encouraging results emphasize that the synergistic effects produced by multiple modification strategies such as nanoscale and morphology engineering, integrated electrode design, and hierarchical porosity control can be comprehensively employed in the right balance to obtain facile kinetics and excellent structural robustness.[[qv: 150b]]

A good number of tin oxides with the inverse spinel structure, M_2_SnO_4_, M = Co, Mn, Mg, Zn, have been studied. In this structure, tetravalent Sn ions occupy tetrahedral sites and bivalent M ions occupy octahedral sites. Doping of electrochemically inactive element prevents structural changes and enhances internal conductivity of Sn to involve all redox sites in conversion reaction.[[qv: 8b,127d]] In some cases, the inactive element can also take part in lithiation reactions, which can significantly affect the voltage curve, cycling performance, and lithiation kinetics.

In recent years, studies on the Li cycling of Zn_2_SnO_4_ prepared by solid state reaction or by hydrothermal synthesis, as well as by co‐precipitation, have been reported, either in pure form or as composites with carbon.[[qv: 143d,158]] It has a large theoretical reversible capacity of 1231 mAh g^−1^ as a result of contributions from the alloying and de‐alloying of Sn and Zn and the conversion reactions involving Sn, Zn and Li_2_O. Spinel Zn_2_SnO_4_ particles prepared via a hydrothermal reaction with 0.4 m NaOH solution at 200 °C for 24 h are composed of uniform cube‐shaped crystallines.^159^ The discharge capacity of the solid was found to be 1384 mAh g^−1^ in the first lithium insertion process, and 580 mAh g^−1^ was retained after 50 cycles. Moreover, HRTEM (high resolution transmission electron microscopy) and XRD data further confirmed the co‐existence of metallic Sn and Zn, and alloy precipitates of about 20 nm in size around the amorphous Li_2_O matrix.

Also, EXAFS (extended X‐ray absorption fine structure) and Sn Mossbauer studies conducted on Co_2_SnO_4_ have confirmed that both alloying and de‐alloying processes and conversion reactions of Sn and Co with Li_2_O are involved in the Li cycling. However, pure cubic spinel Co_2_SnO_4_ (1105 mAh g^−1^) suffers from particle agglomeration and severe volumetric changes that result in poor cyclability.^160^ Very recently, Co_2_SnO_4_/C composites with a mixed amorphous and crystalline structure were prepared for the first time, using a stepwise synthesis technique based on high‐energy ball milling, and showed better capacity retention and rate performance.^161^ Additionally, CoSnO_3_@C nanoboxes synthesized by thermal annealing have exhibited exceptional long‐term cycling stability over 400 cycles for highly reversible lithium storage.^162^

Of particular note, mixed transition metal oxides with a spinel structure like ZnM_2_O_4_ (M = Co, Fe) and CdFe_2_O_4_ are another kind of oxide that has been exploited for both alloying and de‐alloying and conversion reactions.^152^, ^163^

For example, ZnCo_2_O_4_ is a cubic spinel structure oxide with bivalent Zn ions occupying the tetrahedral sites and Co ions occupying the octahedral sites. ZnCo_2_O_4_ has been considered an attractive candidate for the substitution of conventional graphite anodes in lithium ion batteries owing to its superiorities such as better reversible capacity, enhanced cycling stability, and good environmental benignity. So far, nanoparticles and porous nanoflakes of ZnCo_2_O_4_ have been synthesized and shown good first‐cycle reversible capacity as Li‐ion battery anode materials.

The metallic Zn can form an alloy with Li, and with cobalt can participate in the conversion as follows below:[[qv: 163b,164]]

(25) $${\mathrm{ZnCo}}_{2}O_{4}+8{\mathrm{Li}}^{+}+8e^{-}\to \mathrm{Zn}+2\mathrm{Co}+4{\mathrm{Li}}_{2}O$$

(26) $$\mathrm{Zn}+{\mathrm{Li}}^{+}+e^{-}↔\mathrm{ZnLi}$$

(27) $$\mathrm{Zn}+2\mathrm{Co}+3{\mathrm{Li}}_{2}O↔\mathrm{ZnO}+2\mathrm{CoO}+6{\mathrm{Li}}^{+}+6e^{-}$$

(28) $$2\mathrm{CoO}+\frac{2}{3}{\mathrm{Li}}_{2}O↔\frac{2}{3}{\mathrm{Co}}_{3}O_{4}+\frac{4}{3}{\mathrm{Li}}^{+}+\frac{4}{3}e^{-}$$

When nanosized particles of ZnCo_2_O_4_ are electrochemically discharged with Li‐metal, destruction of their crystal structure occurs followed by the formation of metallic Zn and Co nanoparticles and Li_2_O. Upon deep discharge, Zn can reversibly react with Li to form Li–Zn alloy at potentials below 1.0 V, and contribute to the anodic capacity, as shown in Equation (28). In addition, during charge–discharge at potentials above 1.0 V, both the Zn and Co nanoparticles can reversibly react with Li_2_O via the displacement reaction to form nanoparticles of the respective metal oxides. In the most favorable case, CoO can further react reversibly with Li_2_O to form Co_3_O_4_, as shown in Equation (28).

Nano‐phase ZnCo_2_O_4_ prepared by a simple low‐temperature urea based combustion method was reported to yield a reversible capacity of ≈900 and ≈960 mAh g^−1^ when cycled at 25 °C and 55 °C, respectively, corresponding to 8.35 ± 0.05 mol of recyclable Li per mol of ZnCo_2_O_4_.[[qv: 163b]] This is the first time that Zn has been made to contribute to reversible capacity through both alloy formation and displacement reaction in a mixed oxide, and both metallic Zn and Co appear to act as matrix ions. However, the simple structures, poor electric conductivity, and large volume change of the ZnCo_2_O_4_ nanostructures during the electrochemical reaction led to fast capacity decrease.

As for CdFe_2_O_4_, cadmium can also form an alloy with Li in a similar manner to Zn, and under optimal conditions Li_3_Cd is formed during Li cycling.[[qv: 163a]] 8.7 mol of Li can be reversibly cycled (810 mAh g^−1^) at 0.07 C in the range 0.005–3.0 V at ambient temperature. The underlying reaction mechanism is combination of alloying, de‐alloying and conversion reactions of the “Li–Cd–Fe–Li_2_O” composite. However, cadmium is toxic and not suitable for wide application.

In conclusion, transition metal oxides tend to exhibit large polarization and suffer from low electrical conductivity. Additionally, poor capacity retention and unstable solid electrolyte interphase formation at high current densities are common for these electrode materials because of their large volume changes during cycling processes. Effective strategies that have been employed to tackle these problems include fabricating carbon‐based composites to enhance electrical conductivity and designing unique nanostructures that can withstand volume changes.^165^ Carbonaceous materials have been widely used to improve the electrochemical performance of electrode materials for LIBs by forming desirable nanocomposites. Nanostructured transition metal oxides have been proven to exhibit superior electrochemical performance compared with that of their bulk counterparts. In general, nanostructures can provide a reduced distance for ion and electron transport, and larger electrode/electrolyte contact area. Hybrid nanostructures of metal oxides and carbon, in which the nanostructured metal oxides are assembled onto or embedded into the conductive carbon matrix, are under intensive investigation in attempts to achieve high capacity and high rate capability.[[qv: 143d]] Although in‐depth research efforts are in progress to improve the electrochemical performance, many challenges still exist; transition metal oxides offer hope in breaking the bottlenecks of energy storage technologies.

## 4 Multi‐Electron Reactions of Polyvalent Cation Insertion beyond Alkali Metals

Research into lithium‐ion batteries has striven for multi‐electron reactions, which offer the promise of improved energy density.^6^ With lithium, achieving multi‐electron reduction at a single metal redox center faces the challenges of volume expansion and structural collapse resulting from the insertion of more than one lithium ion. It is worth noting that polyvalent metals can undergo multi‐electron reaction.[[qv: 15c]] Polyvalent metals that have been proposed as anode candidates for secondary batteries include magnesium, calcium, zinc, and aluminium, as marked on the periodic table of elements shown in Figure 1. Multivalent secondary batteries with polyvalent ions represent the possibility of a capacity several times higher than that of lithium secondary batteries.

Metallic magnesium has been considered to be an attractive anode material owing to advantageous characteristics such as natural abundance, low cost, high melting point (649°C), and very negative potential of ≈2.4 V vs SHE.[[qv: 15a,15c]] The ionic radius of the divalent magnesium cation is 0.86 Å, similar to the 0.9 Å of monovalent lithium. Magnesium electrochemistry is better understood than that of lithium electrodes in non‐aqueous electrolyte systems. In battery applications, the charge capacity of Mg is 2205 mAh g^−1^, which is lower than that of lithium (3861 mAh g^−1^). However, this inferiority is compensated for by its superior volumetric capacity of 3833 mAh mL^−1^ (compared with 2062 mAh mL^−1^ for lithium metal candidates), enhanced safety, low cost, and easy handling of magnesium metal.[[qv: 15a,15c,166]] Magnesium secondary batteries (MIB) based on the construction Mg|organic electrolyte|intercalation cathode are analogous to the above‐described alkali metal battery systems. Interestingly, a magnesium rechargeable battery offers the possibility of two‐electron reaction through the intercalation of one Mg^2+^ accompanied by a two‐electron reaction per transition metal center.

However, the construction of a MIB faces many challenges, despite the number of similarities in the nonaqueous electrochemistries of Mg and alkali metals. Moreover, magnesium electrochemistry is more complicated. First, in the case of Li electrodes, the passivating surface film that forms on the Li metal may be composed of Li‐ion conductors, products of reaction between polar aprotic solvents and commonly used salt anions, and metallic Mg which blocks Mg^2+^ ion transport.^19^, ^167^ How to prevent the formation of these passive films on metal surfaces is a key problem. Consequently, ordinary organic electrolytes such as carbonates or nitriles are not suitable for Mg ion batteries.^168^ Mg electrodes are reversible only in unique, complex solutions which are capable of allowing electrochemically reversible Mg deposition and dissolution.^19^ It is well known that Mg can be electrochemically reversibly deposited and dissolved in ethereal/Grignard salt solutions. These processes are possible because magnesium surfaces in these solutions do not develop stable passivating surface films.^169^ Efforts have been made to highlight the importance of the electrolyte in magnesium‐containing systems. The discovery of tetrahydrofuran (THF) solvents and magnesium organo–haloaluminate complexes allowed the normal charge/discharge of these batteries.^170^ This system delivered a high room‐temperature conductivity of several millisiemens, close to that of electrolytes used in Li batteries. Meanwhile, many kinds of complex salts were studied for balancing species in the solution. Subsequently, novel solid polymer electrolytes based on Mg(CF_3_SO_2_)_2_ as a salt with high stability and safety were successfully used in a magnesium ion battery, which showed an ambient temperature conductivity of 4.42 × 10^−4^ S cm^−1^, as good as that of an liquid electrolyte.^171^ Very recently, magnesium deposition and dissolution obtained using magnesium bis(trifluoromethane sulfonyl)imide (Mg(TFSI)_2_) with glyme–diglyme has been reported.^172^ The higher than 3.0 V vs Mg^2+^/Mg anodic stability of this electrolyte enables it to be used with high‐voltage cathode materials.

Moreover, selecting suitable cathode materials for Mg ion batteries with high reversible capacity and operating voltage is a thorny issue, even though extensive work has been carried out.^173^ The challenge is finding a positive electrode material that can insert Mg^+2^ to a large extent and thus allows for a high theoretical energy density. A number of excellent reviews on rechargeable magnesium batteries have been conducted, covering either individual cathodes or whole systems.[[qv: 15a,15c,174]] Results obtained in previous studied were tested under different experimental conditions. Nevertheless, in light of very recent research attention, an up‐to‐date review of the breakthroughs in MIBs has become very necessary. Herein, we briefly review the cathode materials which allow reversible electrochemical intercalation of Mg^2+^ and further discuss the problems that hamper the commercialization of MIBs.

**Figure 14:**
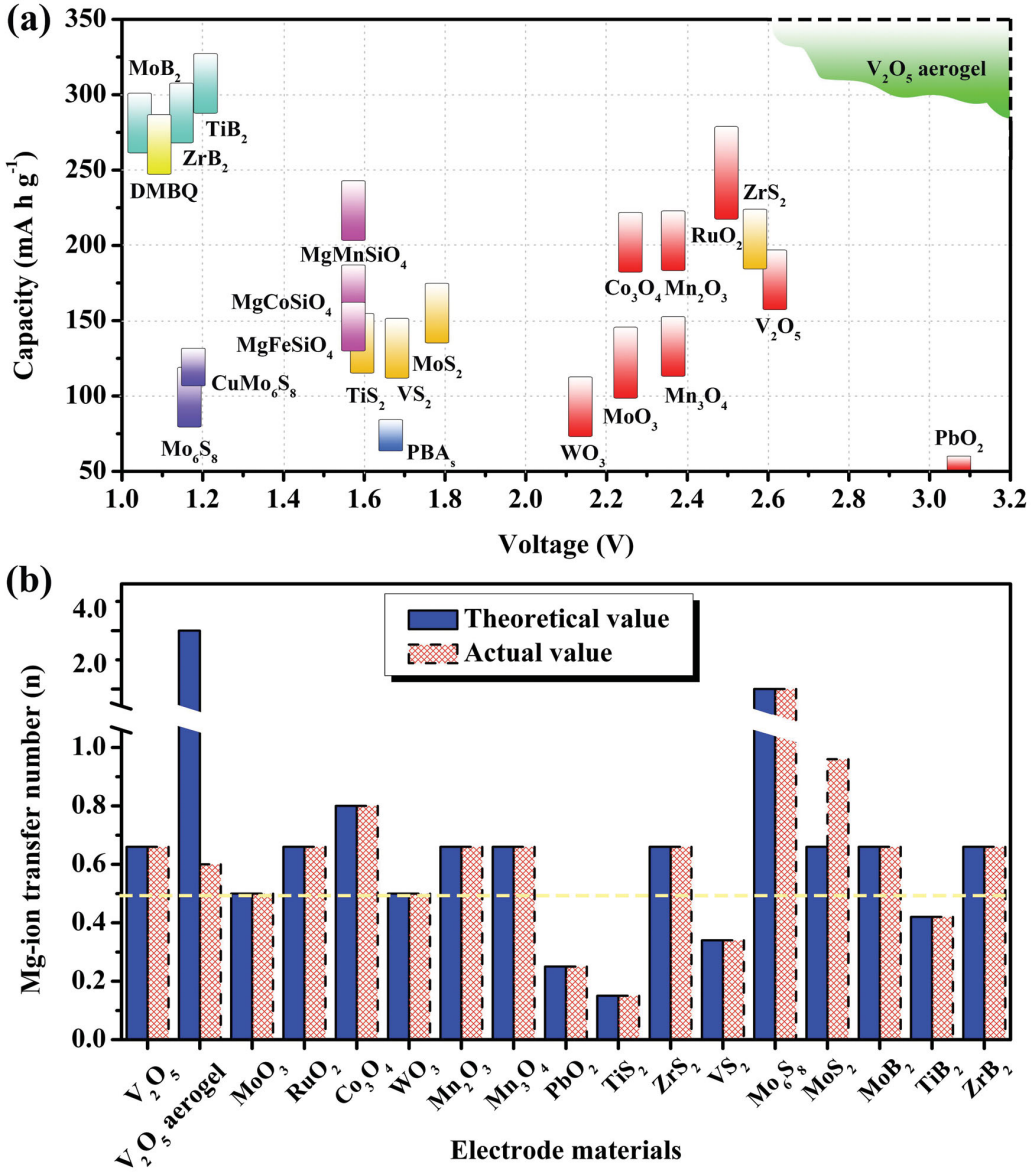
a) Capacity versus voltage for the reported electrode materials for secondary Magnesium batteries; b) intercalated Mg stoichiometry.

**Figure 15:**
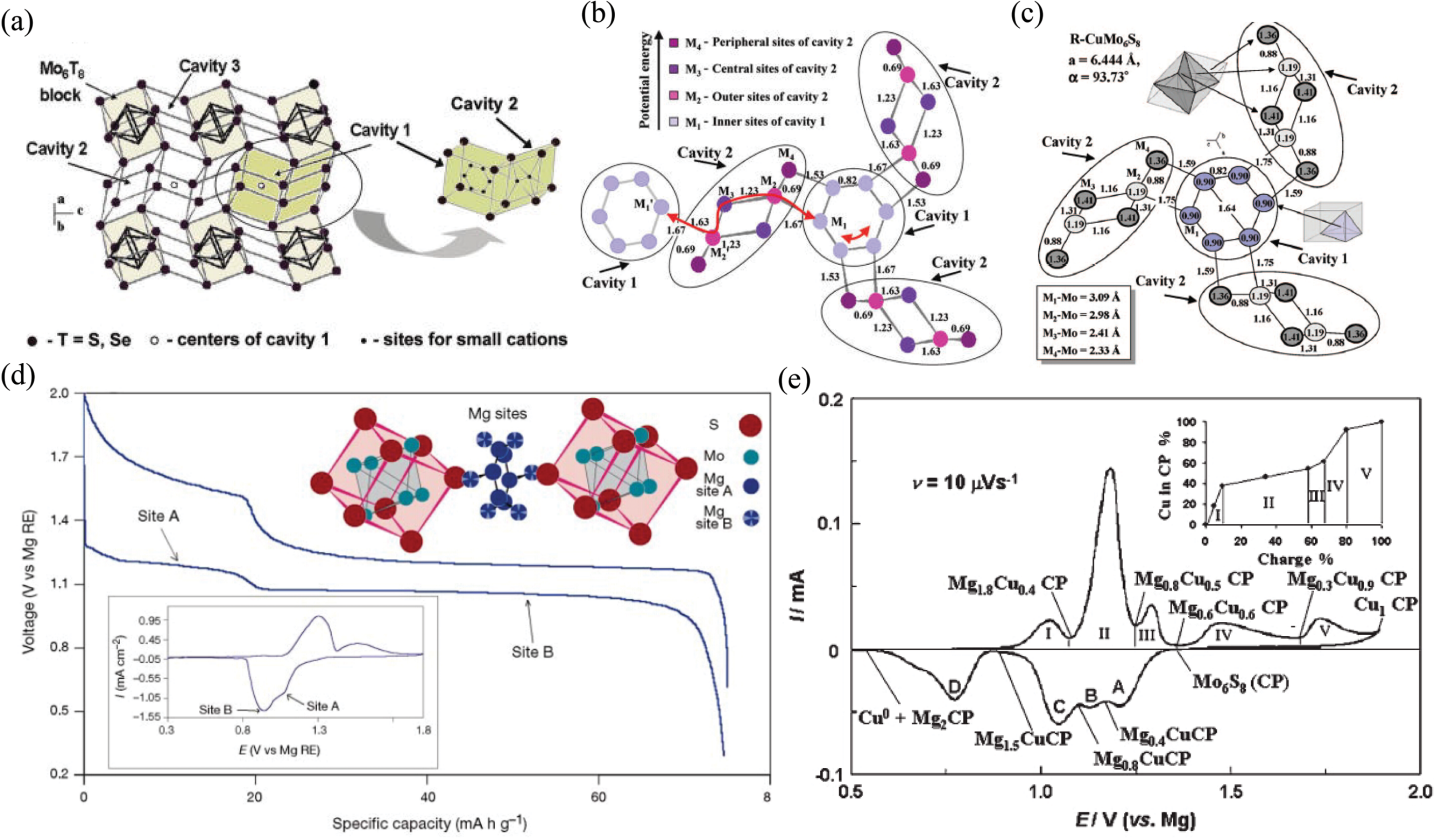
a) Basic Chevrel phases' structure: three types of cavities between Mo_6_X_8_ units. b) Simplified typical map of cation sites in Mo_6_S_8._ c) Maps of cation sites for CuMo_6_S_8_. d) Electrochemical characteristics of Mg^2+^ intercalation into Mo_6_X_8_. Cell discharge current density was 0.3 mA/cm^2^, and CV rate was 0.05 mV/s. e) Cyclic voltammetry curves for the Mg insertion/deinsertion into/from CuMo_6_S_8_ composite electrode measured at 10 μV s^−1^. Phase compositions are indicated. a,b) Reproduced with permission.[[qv: 175b]] Copyright 2009, American Chemical Society. c) Reproduced with permission.^182^ Copyright 2009, American Chemical Society. d) Reproduced with permission.^170^ Copyright 2000, Nature Publishing Group. e) Reproduced with permission.[[qv: 175a]] Copyright 2007, Royal Society of Chemistry.

Various types of materials have been proposed as Mg^+2^ intercalation hosts (as shown in **Figure** **14**), such as Chevrel phases, transition metal‐oxides, sulfides, borides, olivine‐type polyanion compounds, Prussian blue analogues, and organic materials. Among all these cathode candidates, Chevrel phases, M*_x_*Mo_6_X_8_ (M = metal, X = S, Se, Te), are a unique class of host materials that allow fast and reversible insertion of a variety of cations, such as Li^+^, Na^+^, Cu^+^, Fe^2+^, Zn^2+^, Ni^2+^, Co^2+^ and Mg^2+^, at ambient temperatures.^175^ Chevrel phase Mo_6_S_8_ has been proposed as the model cathode material for rechargeable Mg batteries owing to its unusual structure capable of reversibly electrochemically intercalating Mg ions with relatively fast kinetics. Chevrel phases have rhombohedral hexagonal crystal symmetry, space group *R* $\bar{3}$, which consists of octahedral MO_6_ clusters (**Figure** **15**a).^176^ The building block of the MO_6_ cluster unit is common to all these classes of materials (taking Mo_6_S_8_ as an example): 6 tightly packed Mo atoms situated inside an almost regular cubic cage of 8 S atoms.^177^ From an electronic structure perspective, Chevrel phase structures exhibit metallic ground states and Mo_6_S_8_ is intrinsically deficient by 4 electrons. Thus, 4 neutralizing electrons are adopted upon 2 Mg intercalation to stabilize the electron deficient structure and the MO_6_ clusters do not act as classical redox centers like those of other transition metal compounds such as Co_3_O_4_ and MnO_2_.^178^ The Mo_6_S_8_ host can therefore provide fast redistribution of electronic charge and easily compensate for the charge imbalance during insertion, ensuring a high mobility and reversible reaction of Mg^2+^ in the host material.

The first rechargeable Mg battery prototype was established in 2000, and was comprised of Mg*_x_*Mo_3_S_4_ cathodes and an electrolyte based on Mg organohaloaluminate salts.^170^ The progress of magnesium insertion into this host material occurs in two stages.[[qv: 175c]] Two discharge plateaus (at 1.2 V and 1 V) can be observed in the charge–discharge profile, corresponding to Mg^2+^ insertion (shown in Figure 15d). The Mg ions are located in the channels between the two Mo_6_S_8_ units, and the occupied sites are positioned at the inner ring (site 1) and outer ring (site 2) owing to geometrical and electrostatic limitations (simplified cation sites are shown in Figure 15b).^170^, ^179^ The corresponding electrochemical insertion reactions are given:

(29) $${\mathrm{Mo}}_{6}S_{8}+{\mathrm{Mg}}^{2+}+2e^{-}\to {\mathrm{Mg}}_{1}{\mathrm{Mo}}_{6}S_{8}$$

(30) $${\mathrm{Mg}}_{1}{\mathrm{Mo}}_{6}S_{8}+{\mathrm{Mg}}^{2+}+2e^{-}\to {\mathrm{Mg}}_{2}{\mathrm{Mo}}_{6}S_{8}$$

The first stage of intercalation involves Mg occupying one of the lower energy inner sites, and during the full magnesium in the second stage, the second Mg simultaneously occupies one of the outer sites. The prototype Mg battery exhibited more than 2,000 charge–discharge cycles with less than 15 % capacity fading. Nevertheless, its relatively low operating voltage (1.2 V vs Mg) and practical capacity of less than 100 mAh g^−1^ owing to partial Mg trapping in inner channels with low potential energy resulted in a limited energy density. Further efforts are needed to improve the reversible capacity of the system. Mg trapping may be avoided by introducing a foreign metal into the Mo_6_S_8_ host unit to form M*_x_*Mo_6_S_8_, which may improve its reversible discharge capacity.^180^ The electrochemical cycling of M*_x_*Mo_6_S_8_ is reversible based on displacement reactions, and the process becomes more complicated when a foreign metal introduced. Here, Cu*_x_*Mo_6_S_8_ is selected as an example to explain the electrochemical mechanism because it is of practical interest (typical map of cation sites is shown in Figure 15c). The corresponding displacement reaction is shown in Equation (31):

(31) $$2{\mathrm{Mg}}^{2+}+4e^{-}+{\mathrm{Cu}}_{x}{\mathrm{Mo}}_{6}S_{8}\to {\mathrm{Mg}}_{2}{\mathrm{Mo}}_{6}S_{8}+\mathrm{Cu}$$

Mg insertion is much more complicated and various phase compositions are formed during the whole intercalation process (shown in Figure 15e). The extraction of Cu during the electrochemical reaction is reversible and leads to an enhancement of the reversible capacity.^173^, ^181^

3D structures are promising cathode materials for rechargeable magnesium batteries. Prussian blue analogs (PBAs) with an open 3D frame structure are able to support the passage of a large number of Mg^2+^ ions through their channels.^183^ Furthermore, PBAs cathodes can be used in aqueous systems owing to their suitable working potential and stable structure (see Figure 14a for the structure of Ni_3_[Fe(CN)_6_]_3_). Redox‐active organic materials have been investigated because their weaker intermolecular forces than those of inorganic materials may cause them to interact less strongly with Mg^2+^. 2,5‐dimethoxy‐1,4‐benzoquinone (DMBQ) was evaluated for Mg^2+^ storage in a cell with the structure Mg|0.5 m Mg(ClO4)2‐γ‐butyrolactone|DMBQ‐acetylene black‐PTFE.^184^ The DMBQ undergoes two‐electron reaction with the insertion and de‐insertion of 1 Mg^2+^ per mole. The two voltage plateaus of the discharge curve correspond to successive one‐electron reduction steps.

**Figure 16:**
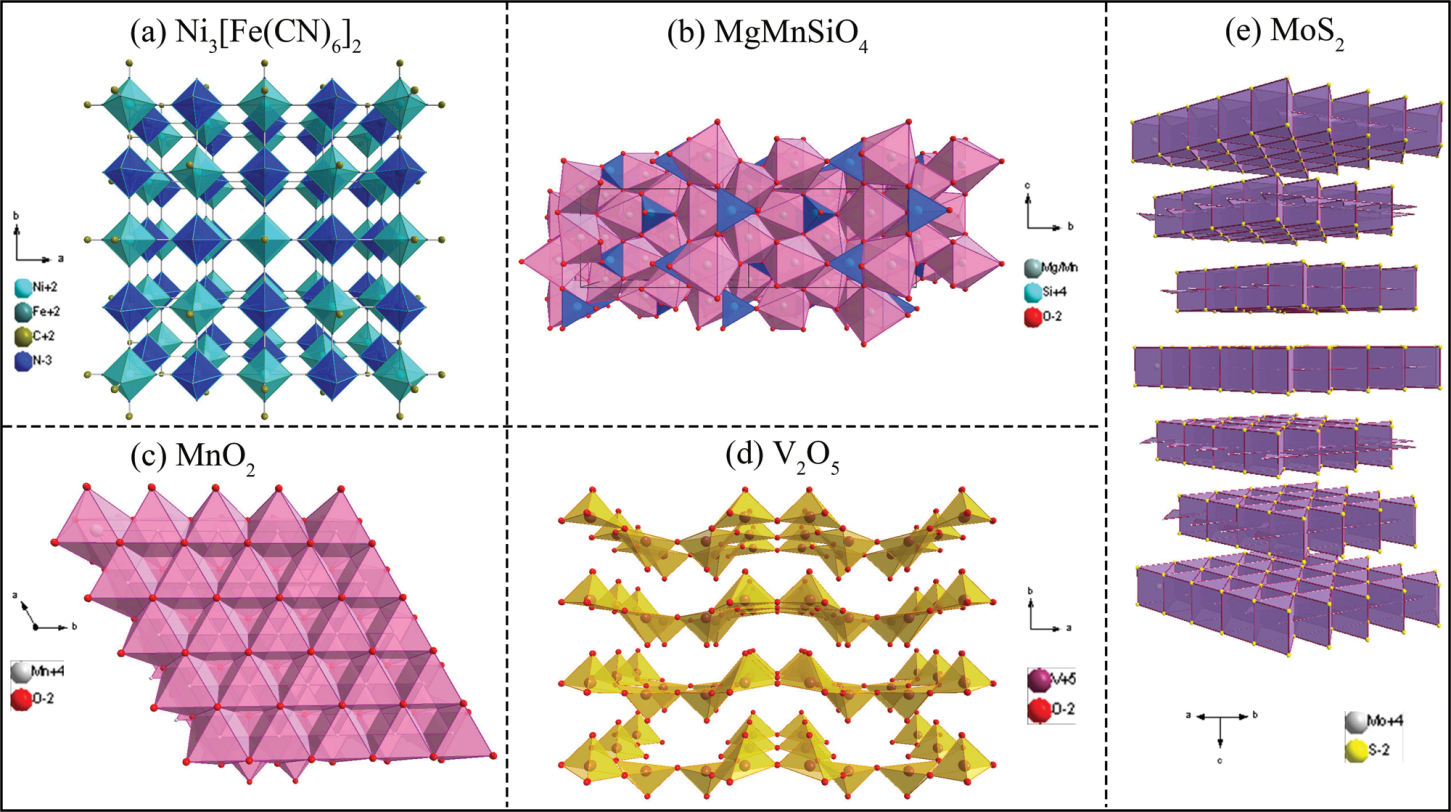
The crystal structure of a) metal–organic framework Ni_3_[Fe(CN)_6_]_3,_ b) olivine MgMnSiO_4_, c) layered MnO_2_, d) layered V_2_O_5_, and e) layered MoS_2_.

A variety of olivine‐type polyanion compounds have been evaluated as cathodes for MIBs owing to their good energy density and production from relatively “green” source materials and synthesis.^185^ Transition metal silicates Mg_x_MSiO_4_ (M = Mn, Fe, Co, etc.) with olivine structure (as **Figure** **16**b) allow for reversible Mg^2+^ ion insertion without structural damage. Among them, Mg_1.03_Mn_0.97_SiO_4_ prepared by a sol–gel method has been reported to exhibit a high discharge voltage plateau at 1.6 V.^185^, ^186^ MgFeSiO_4_, prepared via ion exchange, has delivered a capacity of ≈330 mAh g^−1^ in a magnesium cell, demonstrating the ability to insert Mg^2+^ into FeSiO_4_.^187^ It has also been reported that electrochemical magnesium insertion into Mg_0.5_Ti_2_(PO_4_)_3_ is possible, at up to one Mg atom per host molecule with negligible lattice expansion.^188^

In the early 1980s, Gregory et al. screened the Mg^+2^ intercalation ability of a number of transition‐metal oxides, sulfides, and borides by reduction with dibutylmagnesium.^20^ The magnesium composition and open‐circuit potentials of the materials were determined, and the oxide‐based materials were found to yield high OCVs similar to those of alkali metal systems. A layered MnO_2_ cathode (as Figure 16c) with a self‐standing nanowire structure reported by Sang Bok Lee and co‐workers delivered an initial capacity of 120 mAh g^−1^.^189^ Magnesium insertion into the MnO_2_ was very sluggish because the migration barrier was over 1 eV. With the results of density functional theory (DFT) and HR‐TEM studies, it has been concluded that the magnesium insertion process is a conversion reaction on the surface of MnO_2_ rather than an intercalation reaction.^190^ MnO_2_ with BET surface area above 70 m^2^ g^–1^ delivered a discharge capacity of 250 mAh g^−1^, suggesting that a higher surface area makes it easier to conduct the conversion reaction and enables a greater capacity, although the discharge capacity faded quickly to less than 50 mAh g^−1^ after 10 cycles. Another two dimensionally structured cathode material, molybdenum disulfide, has also attracted wide attention owing to its layered structure (Figure 16e).^191^ Especially, a cell comprising a graphene‐like MoS_2_ cathode and ultrasmall Mg nanoparticle based anode exhibited a high initial capacity of 170 mAh g^−1^ and high operating voltage of 1.8 V.^192^ In this experiment, the larger surface area possessed by the nanosized metallic Mg compared with that of bulk Mg anodes led to the formation of a thinner and porous passivating film. Meanwhile, the graphene sheet‐like structure of the MoS_2_ provided a large interlayer spacing and defect‐free single layers.

Among various cathode materials, vanadium pentoxide (V_2_O_5_) has attracted great attention for many decades owing to its multiple possible valence states (V^5+^ → V^3+^), associated with multiple electron transfer, and its excellent lithium and sodium insertion properties as mentioned above. Research on V_2_O_5_ has shown that it also has potential as a host for polyvalent cationic guests such as Mg^2+^, Al^3+^, and Zn^2+^.^45^ Layered V_2_O_5_ (which consists of layers of VO_5_ pyramids, shown in Figure 14d) provides pathways for insertion/extraction of cations with large ionic radius, and is one of the three cathode materials that will reversibly intercalate Mg^2+^.[[qv: 174d,193]]

Pure V_2_O_5_ crystallizes in the α‐phase and remains stable at high temperature. Chemical insertion tests have revealed that V_2_O_5_ can uptake as high as 0.66 Mg per host molecule with a fairly high voltage of 2.66 V.^20^ As a rechargeable Mg battery cathode material, the overall scheme for the electrochemical reaction can be described as shown in Equation (32).^23^, ^194^

(32) $$x{\mathrm{Mg}}^{2+}+2xe^{-}+V_{2}O_{5}↔{\mathrm{Mg}}_{x}V_{2}O_{5}$$

**Figure 17:**
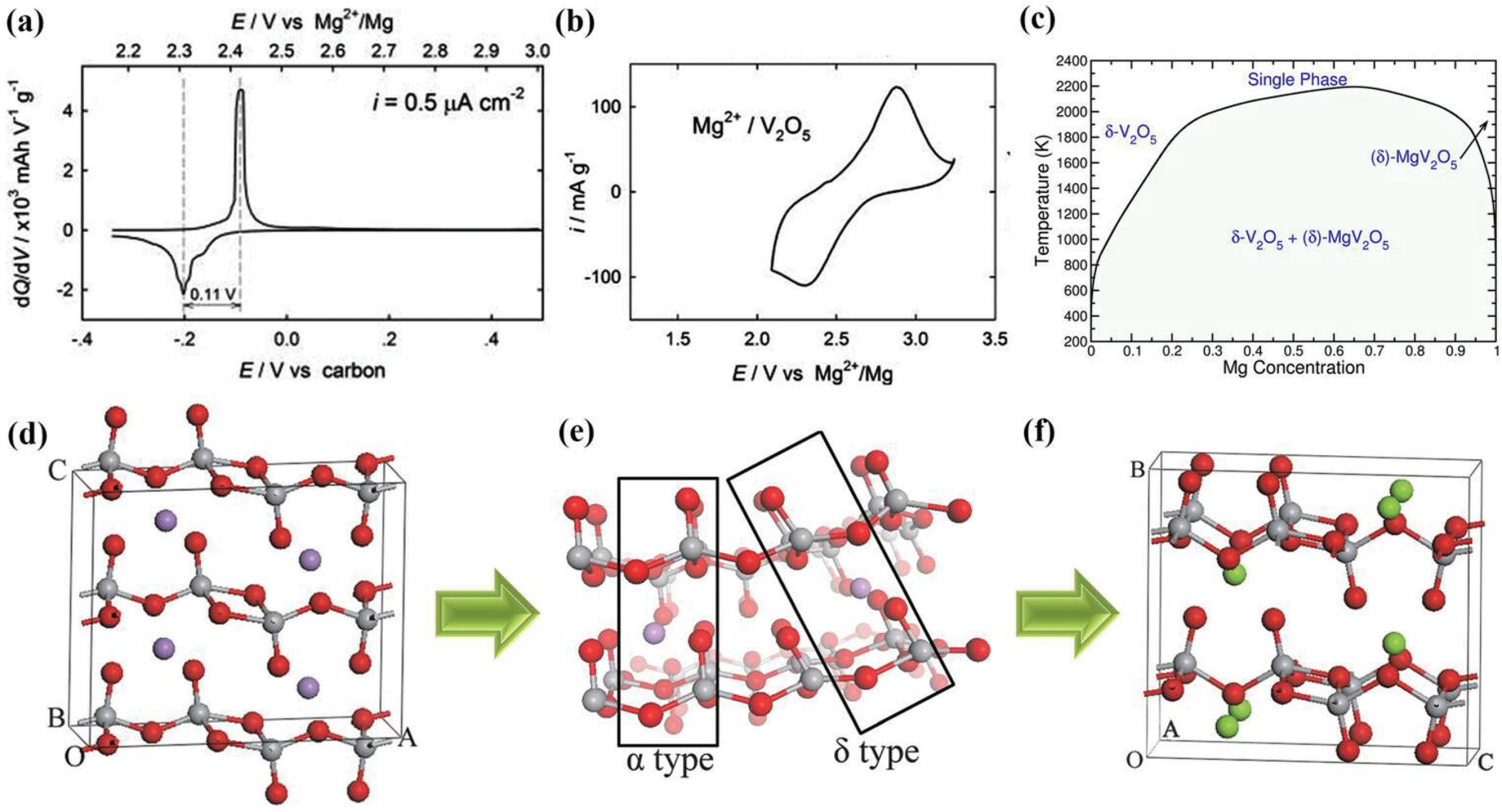
a) Typical differential capacity (*dQ/dV*) plot of the V_2_O_5_ thin‐film electrode, b) cyclic voltammograms for Mg^2+^ intercalation processes into V_2_O_5_ thin‐film electrode. c) Mg‐V_2_O_5_ intercalation phase diagram. d) The crystal structure of the α‐V_2_O_5,_ e) schematic diagram of the α‐V_2_O_5_ to δ‐V_2_O_5_ transformation, f) the crystal structure of the δ‐V_2_O_5_. a,b) Reproduced with permission.^19^ Copyright 2013, American Chemical Society. c) Reproduced with permission.^193^ Copyright 2015, American Chemical Society d,e,f) Reproduced with permission.^197^ Copyright 2014, Royal Society of Chemistry.

Theoretically, electrochemical insertion of Mg^2+^ leads to reduction of V^5+^/V^3+^, corresponding to the formation of the final state MgV_2_O_5_, which has found to form as the pure δ polymorph (shown in **Figure** **17**f). Reversible electrochemical insertion of Mg into crystalline V_2_O_5_ in organic electrolytes at room temperature has been achieved and a superior capacity of over 150 mAh g^−1^ was reported. GO/V_2_O_5_ composites have been reported as a cathode material for rechargeable magnesium batteries with a high discharge capacity of up to 178 mAh g^−1^ at a rate of 0.2C.^195^ Electrochemical insertion of Mg into V_2_O_5_ nanotubes has reported with the manipulation of the average state of V.^196^ The presence of V^3+^ reduced the impedance of the nanotubes, which in turn improved their cycling stability to 71% capacity retention after 20 cycles. The improvement in electrochemical performance was attributed to a lower repulsion between Mg^2+^ and V^3+^ ions relative to that with vanadium in higher oxidation states, which enhanced the transport and structural stability of the nanotubes.

The mechanism of Mg^2+^ insertion into V_2_O_5_ has been studied using highly pure thin films on Pt substrates.^19^ A capacity of 150 mAh g^−1^ was achieved within a narrow window of 2.2–3.0 V, corresponding to 1 e^−^ and 0.5 Mg per mole V_2_O_5_. *dQ/dV* analysis gave clear insight that the magnesiation process is a multi‐step process, and provided distinction between the multiple insertion processes involved with different thermodynamic and kinetic properties (Figure 17a). Moreover, the *dQ/dV* curves also demonstrated that the magnesiation process is appreciably slower than de‐magnesiation. In the case of Mg^2+^ intercalation into V_2_O_5_, only one broad peak was observed (Figure 17b). The intercalation charge is the same for Li^+^ and Mg^2+^ insertion, and the broad peak can be attributed to the merging of several peaks. However, the magnesium insertion process exhibits a larger overpotential than that of Li^+^ insertion owing to its slower kinetics.

Because Mg intercalation is accompanied by twice the number of electrons of Li intercalation, the Mg–V_2_O_5_ intercalation phase diagram is the comprehensive result of the insertion of a different ion and a different number of electrons. The intercalation process at room temperature has also been explored using first‐principles calculations. The equilibrium state of Mg*_x_*V_2_O_5_ was determined to be two‐phase coexistence between the fully magnesiated δ phase and pristine α phase. The equilibrium phase‐separating behavior between these phases was calculated as shown in Figure 17c. At the initial stage of Mg intercalation, only a small amount of Mg has inserted into the host, forming a similar structure to that of α‐V_2_O_5_ (Figure 17d). The initial electrochemical intercalation of Mg^2+^ into bulk V_2_O_5_ crystals indicates an incomplete solid state diffusion which proceeds with surface processes, and the intercalated Mg^2+^ becomes mainly located on the surface of the crystal (with a very small occupation of Mg, *x* = 0.011, in the Mg*_x_*V_2_O_5_ crystal).^194^ With further Mg^2+^ intercalation, the *c* lattice parameter of Mg*_x_*V_2_O_5_ shows a steady expansion, and high Mg concentration intercalation leads to phase transition of α‐V_2_O_5_ to δ‐V_2_O_5_, which involves V_2_O_5_ layers sliding along the *a*‐direction.^193^, ^197^ The main difference between the α phase and δ phase is a rotation of [V_2_O_5_]_n_ layers in the *a*‐direction, as shown in Figure 17e. When the α phase has completely transformed to δ phase, the angle of rotation reaches 22°. Additionally, the layer spacing increases by ≈2% from *x* = 0 (α‐V_2_O_5_) to *x* = 1 (δ‐V_2_O_5_). Still, α‐V_2_O_5_ is a promising cathode material that shows reversible cycling of Mg, although the computed α phase migration barrier indicates poor Mg mobility. So far, all reported experimental efforts have succeeded in reversibly inserting about 0.5 Mg per V_2_O_5_ mole. Reducing the grain size of V_2_O_5_ is an effective method of improving the insertion stoichiometry to x ≈ 0.6.

Moreover, divalent Mg ion insertion into the host structure is difficult owing to its slow solid‐state diffusion, caused by the higher charge density arising from its divalent nature and variations in experimental conditions.[[qv: 15c]] Furthermore, the hopping barrier of Mg ions in α‐V_2_O_5_ is calculated to be 1.26 eV using the nudged elastic band method (NEB, which is an efficient method to determine the minimum energy path and saddle points between given initial and final positions), much higher than the 0.35 eV for Li ions, which explains the slow diffusion observed in experiments.^197^ Particularly interesting is the finding that the amount of electrochemically inserted Mg^2+^ depends on the amount of H_2_O in the electrolyte.^23^ The positive effect of water in the electrolyte arises from a strong solvation effect and partial shielding of the divalent magnesium‐ion charge, which allow its easier insertion and deinsertion into the host.^198^ However, an electrolyte containing a significant concentration of water is likely to be impractical for a secondary Mg battery owing to the reactivity between water and the negative electrode. The importance of the presence of H_2_O in the structure of the cathode material was illustrated by the use of vanadium bronzes; variations in the water content of identical bronzes dried at different temperature were found to be responsible for the difference in their electrochemical properties.[[qv: 198a]] The positive effect of incorporating water into the host crystal structure is believed to originate from a shielding mechanism of Mg^2+^ from direct intercalation with lattice oxygen atoms that further improves the Mg‐ion kinetics.^199^

Besides crystalline V_2_O_5_, promising polyvalent cation insertion results have been reported for V_2_O_5_ aerogels. Aerogels are attractive hosts because of their high surface area and small diffusion distances for a solid‐state material.^200^ V_2_O_5_ aerogels are able to reversibly insert ions of high charge and large ionic radius beyond lithium, such as Na^+^, K^+^, Mg^2+^, Ba^2+^, and Al^3+^.^46^, ^200^ It has been suggested that a pseudocapacitive surface mechanism is responsible for the charge‐storage process in this special non‐crystalline material.^46^ The pesudocapacitance process involves Faradaic reactions and is more prevalent in high‐surface‐area systems with nanostructure, because surface chemistry features such as vacancies and atomic disorder will be amplified. However, in electrochemical experiments, V_2_O_5_ aerogels have been found to reversibly insert only 0.6 mol of Mg^2+^, which is in agreement with the values reported for nanocrystalline V_2_O_5_. The reason for this may be chemically bound water, which can react with the inserted metal in the aerogels and create hydrate compositions of up to V_2_O_5_•5H_2_O. The major difference between aerogel and bulk material is that the diffusion of Mg^2+^ ions into bulk V_2_O_5_ is slow and incomplete, and the majority of the Mg becomes located on the surface of the V_2_O_5_ crystals, whereas the high surface area and short diffusion distance of aerogels yields improved Mg^2+^ diffusion. The measured capacity of 180 mAh g^−1^ corresponds to a reduction of V from +5 to +3 oxidation state. Additionally, aerogels are excellent and versatile hosts for multi‐valent cations such as Mg^2+^, Ba^2+^, and Al^3+^.^200^ However, most of these reported cathode materials uptake no more than 0.5 Mg^2+^ per mole, as shown in Figure 14b, thus the total number of electrons transferred during a practical electrochemical reaction is no more than one. Moreover, the stoichiometry of Mg^2+^, which can be reversibly inserted into the host structure, depends on temperature, morphology, particle size, synthesis method, and electrolyte system.

Research on rechargeable Mg batteries remains a challenge, even though Mg ion batteries are expected to become a new generation of batteries for large‐scale energy storage. The primary reason for the poor electrochemical performance of these batteries is the sluggish diffusion of Mg^2+^ ions.^166^ This limits the rate capability or even the practical insertion of Mg^2+^ ions into the traditional cathodes. Much effort has been concentrated on the modification of ionic transport channels to improve the mobility of Mg^2+^ within intercalation cathodes.[[qv: 15a]] Three main strategies can be concluded to improve the kinetics of Mg transport: 1) Downsizing the particle size of the materials; 2) shielding of the Mg^2+^ ion charge with additional anion groups such as the oxygen of H_2_O molecules; 3) using cluster compounds as unique hosts, such as the Chevrel phases discussed above, that easily attain local electroneutrality.[[qv: 175d]]

A conceptual rechargeable Mg battery in which charge transfer is achieved via simultaneous transport of Mg^2+^ cations and X*^n^* ^−^ anions during electrochemical cycling has been proposed.^201^ The diffusion of monovalent halogen anions in the cathode should be significantly faster than that of divalent Mg^2+^ cations, and therefore ultra‐high rate capability is expected for this battery. This concept has provided a new viewpoint from which to develop Mg and other multivalent (Ca, Al, etc.) batteries.

Calcium batteries are multivalent systems which have a higher cell voltage than magnesium systems and possess a high volumetric capacity of 2073 mAh mL^−1^.^202^ The reversible cycling of a calcium metal anode has not yet been reported owing to a lack of suitable electrolytes. Zinc and aluminum are denser multivalent metals with less negative reduction potentials than those of magnesium and calcium. Research on zinc has mainly focused on aqueous electrochemistry.

Aluminum is the most abundant metallic element in the earth's crust, and its cost is significantly lower than that of most other metals.^203^ Moreover, aluminum‐based batteries exhibit a three‐electron redox reaction and high volumetric capacity of 8040 mAh mL^−1^, making aluminum a strong candidate for energy storage.[[qv: 15b]] The concept of a nonaqueous rechargeable Al‐ion battery in which Al cations can be reversibly intercalated has been proposed.^204^ Rechargeable aluminium‐based batteries offer the possibility of a low cost and low flammability, and their three‐electron‐redox properties are expected to yield a high discharge capacity.[[qv: 15b]]

During the few past years, many kinds of cathode materials have been explored for Al‐ion batteries, such as V_2_O_5_ nanowires, anatase TiO_2_ nanotube arrays, copper hexacyanoferrate, and Chevrel phase M_6_S_8_ as mentioned above.^204^, ^205^ So far, challenges such as the decomposition of the positive material, low cell discharge voltage, and poor cycling performance still restrict the development of rechargeable aluminium‐based batteries.[[qv: 205b,206]] Recently, a novel battery system with an aluminium metal anode, 3D graphitic‐foam cathode, and AlCl_3_/1‐ethyl‐3‐methylimidazolium chloride ([EMICl]) electrolyte has realized a new revolution in battery technology.[[qv: 15b]] The respective reactions that occurred on the positive and negative materials can be expressed as follows:

(33) $$\mathrm{Anode}:\;\mathrm{Al}\;+\;7\;{\mathrm{AlCl}}_{4}{}^{-}\to \;4{\mathrm{Al}}_{2}{\mathrm{Cl}}_{7}{}^{-}\;+\;3e^{-}$$

(34) $$\mathrm{Cathode}:\;C_{n}\left[{\mathrm{AlCl}}_{4}\right]+e^{-}\to C_{n}+\;{\mathrm{AlCl}}_{4}{}^{-}$$

**Figure 18:**
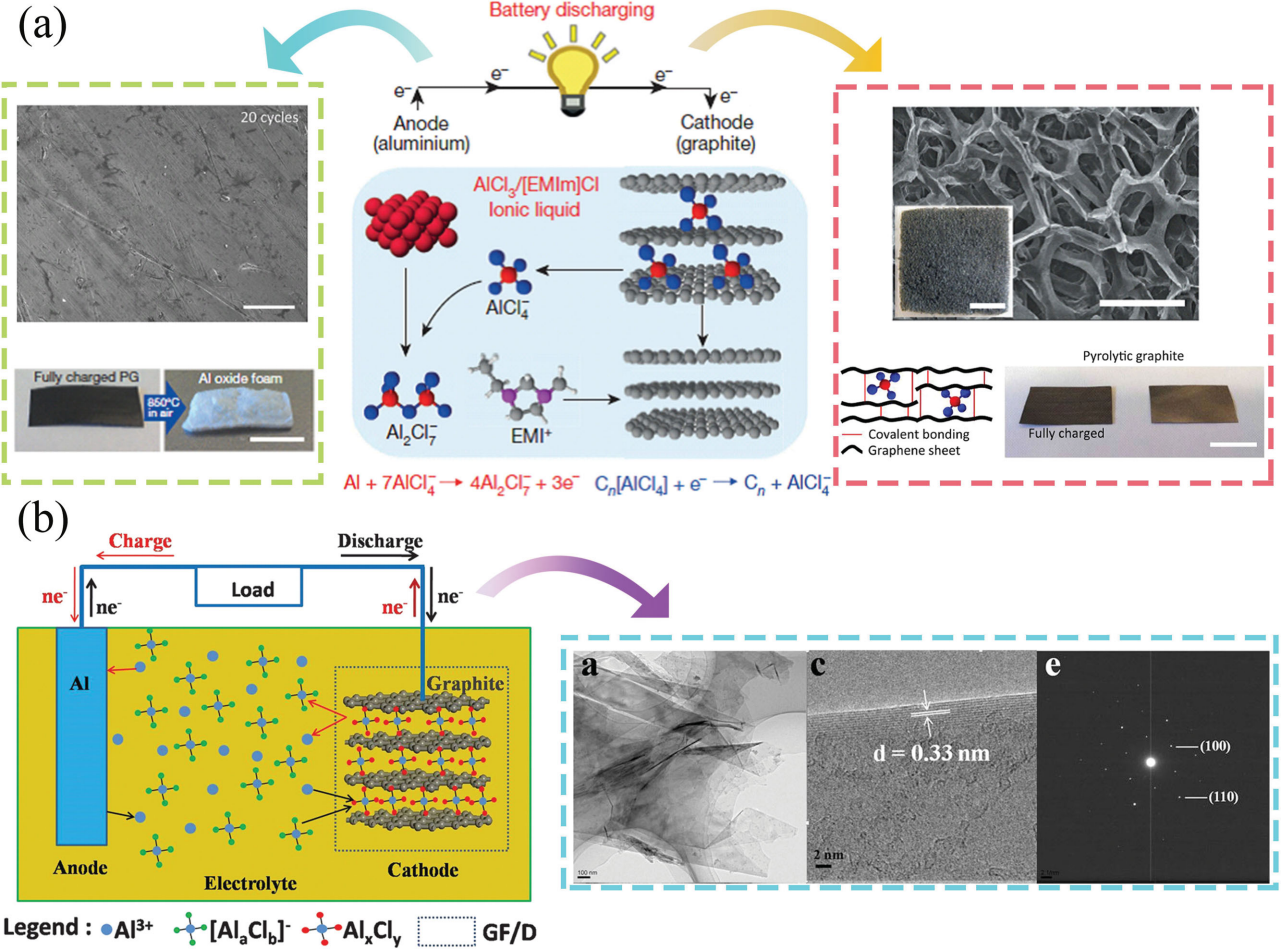
a) Schematic illustration of the Al/graphite cell during discharge, inset in left and right are photos of metal Al and pyrolytic graphite before and after being fully charged, respectively. b) Schematic representation of Al/graphite (carbon paper) full‐cell during the charge and discharge process, inset in right is TEM images of carbon paper. Commun. a) Reproduced with permission.[[qv: 15b]] Copyright 2015, Nature Publishing Group. b) Reproduced with permission.^207^ Copyright 2015, Royal Society of Chemistry.

The aluminum electrode and pyrolytic graphite electrode, which had a foam structure (shown in **Figure** **18**a), greatly decreased the diffusion length for electrolyte penetration and facilitated a rapid charge/discharge rate. As a result, this Al‐ion battery with three electron reaction exhibited a specific capacity of about 70 mAh g^−1^ and amazing cycle life of more than 7500 cycles at a current density of 4000 mA g^−1^. The cell also exhibited well‐defined discharge voltage plateaus in the ranges of 2.25–2.0 V and 1.9–1.5 V, which were unprecedented when compared with past Al‐ion batteries. Moreover, the Al‐ion batteries had an ultrafast Al^3+^ ions insertion/extraction ability that exhibited stable cycling performance both at low and high current densities. Based on the “multi‐coordination ion/single ion intercalation and deintercalation” theory described above, a similar battery system was designed using Al foil as the anode and carbon paper as the cathode (seen in Figure 18b).^207^ In the discharge process, metallic aluminium was oxidized into Al^3+^ ions at the anode and Al^3+^ gained electrons to form Al*_x_*Cl*_y_* at the cathode. The inverse process occurred during charging. This kind of Al‐ion battery delivered a capacity of 69.92 mAh g^−1^ even after 100 cycles at a current of 100 mA g^−1^. Moreover, it showed a high average voltage plateau of ca. 1.8 V vs Al^3+^/Al. Such rechargeable Al‐ion batteries possess advantages including low cost, high performance, and environmental friendliness, which make them promising candidates for energy storage in the future. Moreover, the Al^3+^ cation has a smaller radius (5.35 Å) than that that of the Li^+^ cation (7.6 Å) and Mg^2+^ cation (7.2 Å), which indicates a possibility of using Al^3+^ cation as a guest in intercalation chemistry and with lower volume expansion of the host compounds upon tri‐valent aluminum ion insertion.^204^

Research into multivalent chemistries is helpful for gaining insight into new intercalation materials based on multivalent cations as guest species for rechargeable batteries. Movement of one multivalent cation from anode to cathode induces multiple charge transfer in the external circuit as compensation.^206^ However, research progress has been hindered by difficulties in identifying suitable electrolytes and cathode materials. The strong columbic interaction with the host lattice hinders the insertion kinetics of multivalent cations, which places restrictions on the selection of potential cathode materials.[[qv: 175d,205a]] The poor utilization of intercalation compounds during polyvalent cation insertion can be traced to diffusion limitations attributed to the large charge density of these cations.^208^ The charge density of the cations is affected by localized cation‐cation repulsion and cation‐cation attractive forces from anions during the intercalation process, and these interactions increase with the iconicity of the M–X bond. Practical methods of improving the poor diffusion involve using hydrated compounds and fabricating intercalation compounds with smaller diffusion distances. Additionally, Mg^2+^ insertion into V_2_O_5_ nanocrystals and V_2_O_5_ aerogel exemplifies the special applications that are possible. Based on the current knowledge of multivalent battery chemistries, future research should be focused on finding suitable cathode materials with higher capacity and working potential.

## 5 Multi‐Electron Reactions on Metal–O_2_ Batteries

Energy density is an important indicator in the development of the next generation of energy storage devices. Another of the proposed post lithium‐ion technologies, metal–air batteries have stood out as promising electrochemical energy storage and conversion devices.[[qv: 16c,209]] In metal–air batteries, the intercalation material at the cathode is replaced with a catalytically active oxygen reduction reaction (ORR) electrode and an oxygen evolution reaction (OER) porous electrode, and operates using the continuous and almost inexhaustible oxygen supply from the surrounding air.[[qv: 16a,210]] The overall chemical reaction occurring inside a metal–air battery can be described as follows (Equation (35)).^211^ Multi‐electron charge transfer endows this new type of battery system high energy density.

(35) $$\mathrm{Me}+\frac{x}{2}O_{2}↔{\mathrm{MeO}}_{x}$$

The reason why metal–air batteries can be regarded as multi‐electron transport systems is related to the series of complex electrochemical reactions that occur on the air cathode. These reactions involve the participation of multiple electrons and generate several oxygen‐containing compounds.^212^

**Figure 19:**
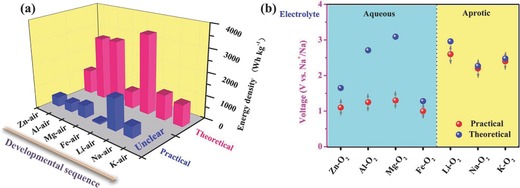
Comparison of different types of metal–air systems.

There are several kinds of metal–air batteries based on different reaction mechanisms with different metal species.^213^ Metal–air batteries are divided into two types according to electrolyte: cell systems with an aqueous electrolyte, and those with an aprotic electrolyte. A comparison of developed metal–air systems is shown in **Figure** **19**. Notably, different reaction mechanisms occur in aqueous and organic environments. Metals such as Mg, Al, Zn, and Fe are appropriate for aqueous systems, and **Table** **2** compares the anode reactions and standard potential of these metal–air systems. Aqueous systems are somewhere between traditional batteries and fuel cells. Owing to their better dynamics and lower overpotentials, neutral or alkaline aqueous solutions are very suitable for aqueous systems. Additionally, the ionic conductivity of aqueous systems is much higher than that of organic systems. However, the limited electrochemical window between the evolution potentials of hydrogen and oxygen is a negative factor. Reactions between metallic lithium and aqueous electrolytes are dangerous, which imposes high requirements for the metal protection layer.

**Table 2:** Electrochemical anode reactions and the theoretical standard potential of selected metal–air batteries with aqueous alkali electrolytes.[[qv: 16a,216a]]

Metal–air batteries	Anode reactions	E^o^/V
Mg–air	(a) 2Mg + 4OH^–^ → 2Mg(OH)_2_ + 4e^–^	–2.69
Al–air	(a) Al + 3OH^–^ → Al(OH)_3_ + 3e^–^	–2.31
Zn–air	(a) Zn + 2OH^–^ → ZnO + H_2_O + 2e^−^	–1.25
Fe–air	(a) Fe + 2OH^–^ → Fe(OH)_2_ + 2e^–^	–0.877
Li–air	(a) Li + OH^–^ → LiOH + e^–^	–2.95

Mg–air batteries have a high theoretical energy density of 2840 Wh kg^−1^ and a high theoretical voltage of 3.09 V. However, practically attainable values are much lower owing to the parasitic corrosion reaction that evolves hydrogen gas at the metallic negative electrode.[[qv: 16c]] Considerable efforts have been directed towards the development and commercialization of primary and mechanically rechargeable batteries. Mg–air batteries are not electrically rechargeable with the reversible oxygen reduction and evolution reactions because the electrodeposition of magnesium is not thermodynamically feasible in aqueous electrolytes.[[qv: 16c]] These versions are also applicable to Al–air batteries.^214^ Mg–air batteries containing nonaqueous electrolytes are unable to carry out the reversible oxygen reaction because the probable discharge product MgO is electrochemically irreversible as well as nonconductive and insoluble in organic electrolytes.[[qv: 15c]] Zinc is the most active metal that can be plated from an aqueous electrolyte. Traditional zinc–air batteries are non‐rechargeable. Primary Zn–air batteries have been the most successful among the primary battery systems owing to their noted high theoretical energy density of 1084 Wh kg^−1^.^213^ The development of electrically rechargeable Zn–air batteries relies on both zinc dissolution and deposition, and satisfactory bifunctional air catalysts that are capable of catalyzing both the oxygen reduction and evolution reactions effectively.[[qv: 209b,215]] Fe–air batteries stand out as promising candidates because of their low cost, sustainability, and environmental friendliness.^211^ With further improved efficiency and cycle life, the Fe–air battery is expected to become viable for grid‐scale electrical energy storage applications, although its energy density of 50–75 Wh kg^−1^ is relatively low.^216^

**Figure 20:**
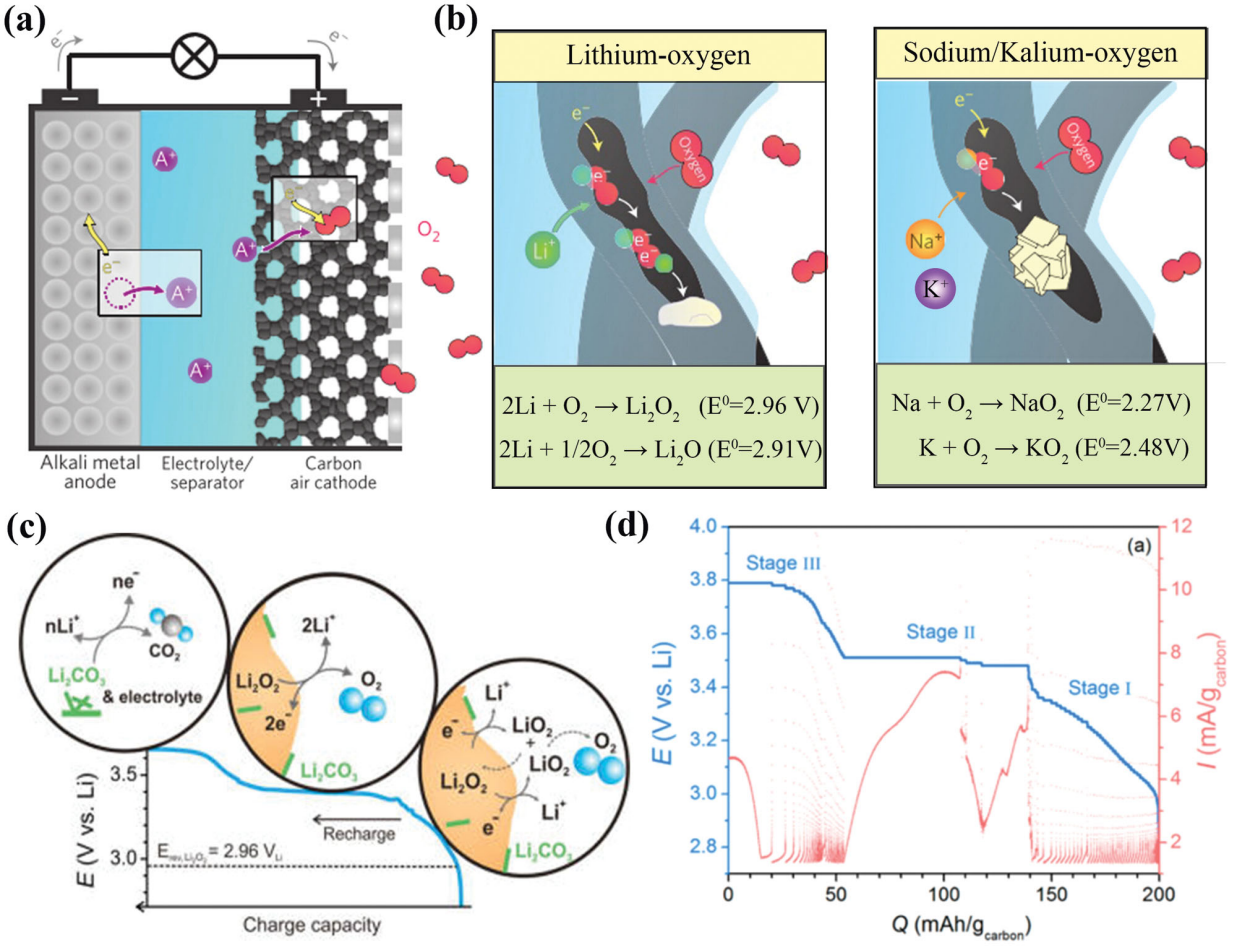
a,b) The general function principle of an alkali‐oxygen battery. c) Proposed reaction mechanism of Li–O_2_ recharge, d) Potential steps (blue) and corresponding current response (red) during PITT measurements. a,b) Reproduced with permission.^218^ Copyright 2013, Nature Publishing Group. c,d) Reproduced with permission.^219^ Copyright 2013, American Chemical Society.

Non‐aqueous metal–air batteries such as Li–O_2_, Na–O_2_, and K–O_2_ are gaining rapidly increasing attention. In fact, most non‐aqueous metal–air systems based on alkali metals can be referred to as A–O_2_ batteries because most laboratory research has involved the use of an O_2_ tank, although many papers refer to the systems as metal–air. Non‐aqueous A–O_2_ batteries have a short history and research is still in progress. The general operating principles of an A–O_2_ battery are shown in **Figure** **20**a,b. During discharge, metal A is oxidized to a soluble A^+^ cation at the anode/electrolyte interface, and the electron is transferred to the outer circuit. At the cathode side, oxygen is reduced to an O_2_^−^ species (superoxide radical) that may form an alkali metal superoxide (AO_2_) in the presence of A^+^ cations. In fact, the reactions of lithium, sodium, and potassium with oxygen are quite different, despite their close chemical relationship.^217^

Because LiO_2_ is highly unstable, it is only found as an intermediate species in Li–O_2_ cells. The Li–O_2_ system has a theoretical specific capacity of 3458 Wh g^−1^, which will fully meet the future battery needs. The possible reaction mechanism in Li–O_2_ batteries based on non‐aqueous electrolyte systems is envisioned as follows:

(36) $$\begin{array}{l} 4{\mathrm{Li}}^{+}+4e^{-}+O_{2}\to 2{\mathrm{Li}}_{2}O\\ \;(4e^{-}\;\mathrm{transferred}\;\mathrm{for}\;\mathrm{per}\;O_{2}\;\mathrm{molecular},\;E^{0}=2.91\;V) \end{array}$$

(37) $$\begin{array}{l} 2{\mathrm{Li}}^{+}+2e^{-}+\;O_{2}\to \;{\mathrm{Li}}_{2}O_{2}\\ \;(2e^{-}\;\mathrm{transferred}\;\mathrm{for}\;\mathrm{per}\;O_{2}\;\mathrm{molecular},\;E^{0}\;=\;2.96\;V) \end{array}$$

The two respective electron transfer processes involve four‐electron and two‐electron pathways, which produce different reduction products. Oxygen is reduced on discharging to form Li_2_O_2_ or Li_2_O. These reactions are usually called “ORRs” (oxygen reduction reactions). The generated lithium oxides are electrochemically decomposed to Li and O_2_ on charging. Generally, the formation of Li_2_O_2_ is an accepted mechanism which may involve two intermediate processes:

(38) $${\mathrm{Li}}^{+}+e^{-}+O_{2}\to {\mathrm{LiO}}_{2}$$

(39) $${\mathrm{Li}}^{+}+e^{-}+{\mathrm{LiO}}_{2}\to {\mathrm{Li}}_{2}O_{2}$$

It cannot be ignored that the solubility of the LiO_2_ intermediate is an important factor in the pathway of O_2_ reduction.

Meanwhile, the electrochemical decomposition of Li_2_O_2_ to Li and O_2_ on charging reported by Ogasawara and co‐workers,^220^ referred to as the “oxygen evolution reaction” (OER), can be written as:

(40) $${\mathrm{Li}}_{2}O_{2}\to 2{\mathrm{Li}}^{+}+2e^{-}+O_{2}$$

There are a number of other theories used to explain the cathodic reaction process, such as the generation and decomposition of LiO_2_.^221^ As shown in Figure 19c,d, the OER process of Li_2_O_2_ can be divided into three stages.^219^ Stage I is a de‐intercalation process corresponding to the formation of LiO_2_‐like species on the surface (Equation (41)) via a solid‐solution route. These LiO_2_‐like species further evolve O_2_ via Equation (42). Thus, the OER process in stage I yields an overall two‐electron reaction as shown in Equation (43).

(41) $${\mathrm{Li}}_{2}O_{2}\to {\mathrm{LiO}}_{2}+{\mathrm{Li}}^{+}+e^{-}$$

(42) $${\mathrm{LiO}}_{2}+{\mathrm{LiO}}_{2}\to {\mathrm{Li}}_{2}O_{2}+O_{2}$$

(43) $${\mathrm{Li}}_{2}O_{2}\to 2{\mathrm{Li}}^{+}+2e^{-}+O_{2}$$

Stage II shown in Figure 20c,d is a flat plateau, which is caused by the oxidation of bulk Li_2_O_2_ particles. Lastly, stage III exhibits a rising charge plateau assigned to the decomposition of carbonate‐type byproducts and electrolyte.

In a Na–O_2_ cell, even though the formation of peroxide Na_2_O_2_ is thermodynamically favored, the formation of NaO_2_ requires the transfer of only one electron per formula unit and as such will be kinetically preferred over the two‐electron transfer towards the peroxide. Thus, a stable superoxide NaO_2_ is formed according to the relative active cathode electrochemistry of the Na‐air battery, as described as follows:^218^

(44) $$\begin{array}{l} {\mathrm{Na}}^{+}+e^{-}+O_{2}↔{\mathrm{NaO}}_{2}\\ \;(1e^{-}\;\mathrm{transferred}\;\mathrm{for}\;\mathrm{per}\;O_{2}\;\mathrm{molecular},\;E^{0}=2.27\;V) \end{array}$$

As for potassium, KO_2_ is thermodynamically stable and commercially available. Within a controlled potential range, the reaction is expected to be:

(45) $$\begin{array}{l} K^{+}+e^{-}+O_{2}↔{\mathrm{KO}}_{2}\\ \;(1e^{-}\;\mathrm{transferred}\;\mathrm{for}\;\mathrm{per}\;O_{2}\;\mathrm{molecular},\;E^{0}=\;\;2.48\;V) \end{array}$$

The potential of the formation of K_2_O_2_ is 0.28 V lower than that of KO_2_, however, formation of K_2_O_2_ will lead to passivation of the electrode and result in a large negative polarization.^222^ Na–O_2_ and K–O_2_ batteries can form the stable superoxides NaO_2_ and KO_2_ (shown in Figure 20b), respectively, which could reduce decomposition of the electrolyte and carbon cathode during the discharge process.^223^ The quasi‐reversible one‐electron reaction between the O_2_/O_2_^−^ redox couple is favored owing to its lower energy barrier compared with that of O_2_/O_2_^2−^.^222^, ^224^ Throughout the charging and discharging cycle, the charge overpotential in both Na–O_2_ and K–O_2_ systems is lower than that in the Li–O_2_ system.

The current status of catalytic materials and structures for air electrodes has been reported in many important journals. Oxygen catalyst materials still face great challenges. Electrocatalysts for the ORR and OER play key catalytic roles and determine the power, energy density, and energy efficiency of metal–air systems based on both aqueous and nonaqueous electrolytes. Several groups of oxygen catalysts, including transition metal oxides, functional carbon materials, metal oxide–nanocarbon hybrid materials, metal–nitrogen complexes, transition metal nitrides, conductive polymers, and precious metal alloys have been discussed.^210^ Transition metal oxides such as MnO_2_, Co_3_O_4_, and Fe_3_O_4_ have been used as catalysts and further studied.^225^ For example, a 3D graphene–Co_3_O_4_ cathode was prepared by growing Co_3_O_4_ on nickel foam with graphite deposits.^226^ This special structure without binder provided interconnected channels and superior catalytic activity, and delivered a high specific capacity of 2453 mAh g^−1^ at 0.1 mA cm^−2^. Some metal oxides are also suitable for air cathodes. Spinel MFe_2_O_4_ (M = Co, Ni) and multi‐walled carbon nanotube (CNT) composites synthetized by simple hydrothermal method have exhibited excellent electrochemical performance.^227^ In these composites, the MFe_2_O_4_ exhibited high catalytic activity while the CNTs offered facile electron transport pathways. Lu and co‐workers designed sandwich structured Pd/MnO*_x_*/Pd nanomembranes synthetized by a roll‐up technique.^228^ The encapsulation of the MnO_x_ layer by the two Pd outer layers improved the power efficiency of the battery to 86%, and lowered the overpotential to 0.2 V. This work can be regarded as the first attempt to create a composite of two different types of catalyst.

A novel nitrogen‐doped exfoliated graphene air electrode with high surface area (2980 m^2^ g^−1^) and ORR activity has exhibited excellent practicability in metal–air batteries.^229^ Sun and co‐workers^230^ proposed a 3D nanostructured air electrode with full space utilization, which led to an enhanced discharge capacity. However, studies suggested that a parasitic side product with a carbonate‐like structure coated the air electrode during the cycling. This by‐product may affect the cycling stability of the cell.

Besides catalysts, non‐aqueous solvents for metal–air batteries have also been studied in detail. Many kinds of organic solvents such as dimethyl sulfoxide (DMSO), acetonitrile (MeCN), dimethoxyethane (DME), and tetraethylene glycol dimethyl ether (TEGDME) have been tested in Li–air batteries.[[qv: 221c,231]] Zhou and co‐workers developed an all‐solid‐state Li–air battery using Li_1+_ *_x_*Al*_y_*Ge_2−_ *_y_*(PO_4_)_3_ (LAGP) as the electrolyte and a LAGP/single‐walled carbon nanotube (SWCNT) composite as the air cathode.^232^ Recently, an integrated solid‐state electrolyte and cathode structure used in a Li–S battery was reported by Zhao et al. Their proposed structure effectively reduced the battery volume, increased the cathode porosity, and reduced the internal resistance of the battery.^233^ Solid‐state electrolytes are very suitable for Li–air batteries because they can prevent side reactions on the Li metal anode and enhance the safety of the batteries. Nonvolatile solid electrolytes can also be used for long‐life cells.

In summary, metal–air batteries are a promising energy storage technology owing to their high potential energy density. However, technical issues relating to the catalysts and the use of O_2_ still exist, and there is a big gap between the actual and theoretical capacity. Rational design of the electrode structure is important because the reactions involve the diffusion of O_2_ and precipitation of the discharged products. Pure oxygen is too difficult to handle in practical application, and substituting pure oxygen with air (a mixture containing O_2_, CO_2_, H_2_O, etc.) may lead to irreversible reactions. For the sake of improving the actual capacity of air batteries, further research aiming at overcoming these technical issues is urgently needed, and the details of the operating mechanism should be deeply investigated.

## 6 Multi‐Electron Reactions Occurring in Li–S Batteries

Lithium sulfur batteries have been extensively studied because they have the potential to reach practical energy densities of 500–600 Wh kg^−1^ within the next few years.^234^ Li–S batteries break through the limitations in the capacities of insertion compounds currently used in both lithium and sodium batteries because sulfur offers a high theoretical capacity of 1672 mAh g^−1^ with a two‐electron redox reaction per atom.[[qv: 234b]] Additionally, the abundant resources and environmental friendliness of these systems are beneficial in terms of sustainable development.

The first room temperature Li–S systems can be traced back to 2009, which successfully enhanced the capacity and cycling stability capable for batteries using ordered mesoporous CMK‐3 composited with sulfur.^235^ Assuming that the sulfur molecule completely converts to Li_2_S in an ambient environment, the total reaction can be inferred as follows:

(46) $$S_{8}+16{\mathrm{Li}}^{+}+16e^{-}\to 8{\mathrm{Li}}_{2}S$$

**Figure 21:**
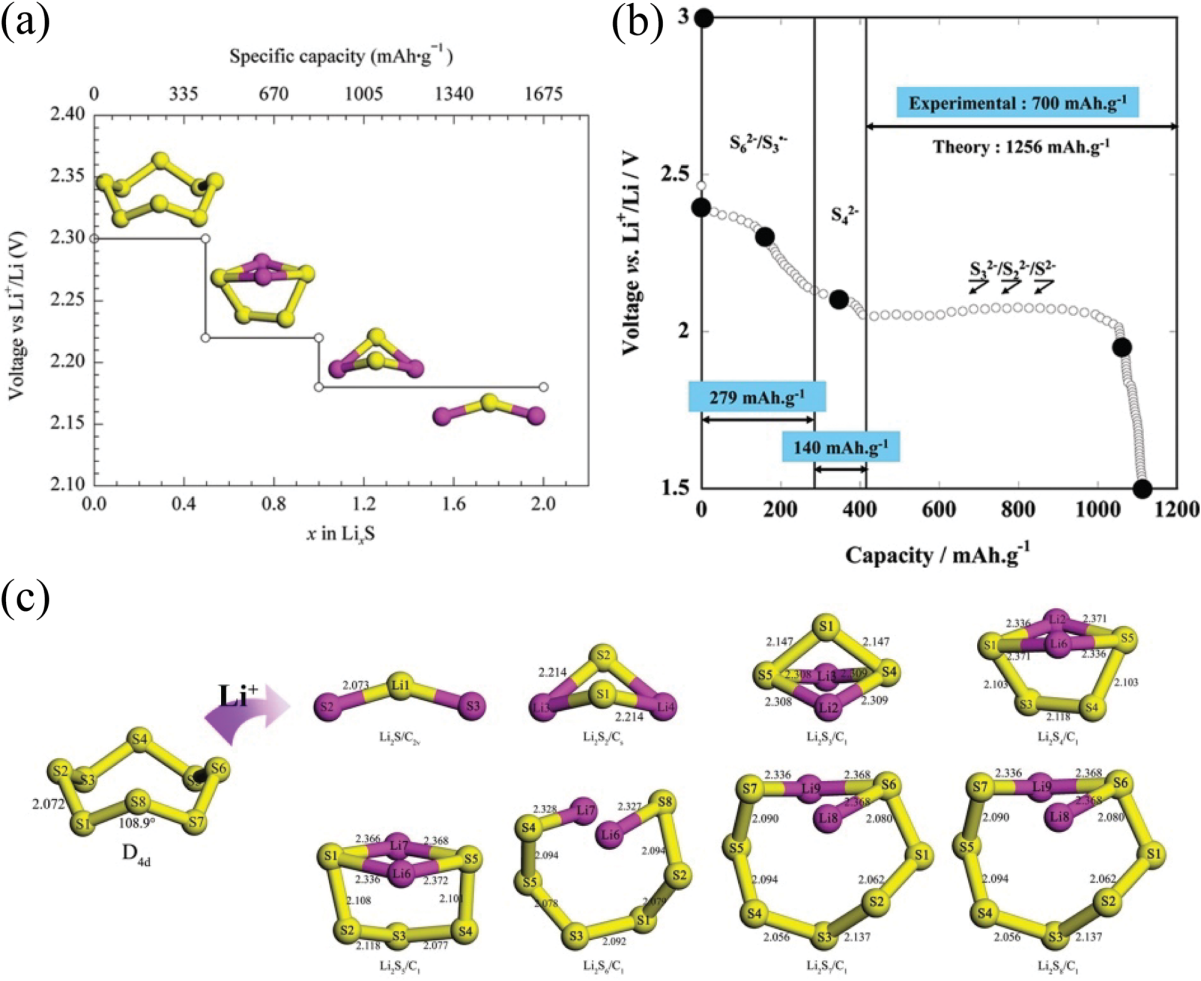
a) Structures and corresponding discharging potential plateaus of lithium polysulfide obtained by computer simulation based on DFT; b) Sulfur reduction products and their discharging potential plateaus, as well as the specific capacities corresponding to each step; c) Optimized geometries, structural parameters of lithium polysulfide. a) Reproduced with permission.^237^ Copyright 2013, Elsevier. b) Reproduced with permission.^239^ Copyright 2012, American Chemical Society.

Through theoretical calculation using the Nernst equation, the reaction process can be divided into a two‐stage scheme.^236^ The high plateau corresponds to the conversion between S_8_ molecules and S_4_^2−^ ions with 4e^−^ transfer, which delivers a capacity of 419 mAh g^−1^. The low plateau provides a high capacity of 1256 mAh g^−1^ and corresponds to the reduction from S_4_^2−^ to S^2−^ with 12e^−^ transfer. Therefore, Li–S batteries exhibit a high theoretical capacity of 1675 m Ah g^−1^ corresponding to the transfer of 16 mol of electrons. According to the further reported results of density functional theory (DFT),^237^ the three‐step reaction process can be described as: S_8_ → S_4_^2−^ → S_2_^2−^ → S^2−^, as shown in **Figure** **21**a. The similar equilibrium potentials of the two latter steps mean that they appear as one discharge plateau.

The complex reaction process at the sulfur electrode during the lithiation can be distinguished from other multi‐electronic materials, which is a key factor for classifying it as a separate category. Actually, the various forms of sulfur will be obtained in different reaction environments (operating temperature, electrolyte system, and so on).[[qv: 234b,238]] Many studies have identified the presence of abundant polysulfide species in solution arising from disproportionation reactions. For instance, Barchasz et al.^239^ used a series of methods including high‐performance liquid chromatography, electron spin resonance spectroscopy, and UV−visible absorption spectroscopy to investigate the composition of the electrolyte at different discharge stages, which provided indirect evidence of the complex multistep process of the discharge mechanism of sulfur electrode. Their results divided the sulfur reduction process into the three steps shown in Figure 21b. In the first step, S_8_ gains two electrons to form S_8_^2−^ ions at 2.4 V. However, these S_8_^2−^ ions may undergo a disproportionation reaction to form S_5_^2−^ and S_3_^•−^. In the second step, sulfur free radicals S_3_^•−^ and S_6_^2−^ species are reduced to S_4_^2−^ and S_3_^2−^ ions at 2.1 V. The disproportionation reactions occurring during the first two steps can be summarized as follows:

(47) $$2S_{n}{}^{2-}↔S_{m}{}^{2-}\frac{n\;-\;m}{8}S_{8}$$

(48) $$2S_{n}{}^{2-}\to S_{n+m}{}^{2-}+S_{n-m}{}^{2-}$$

(49) $$S_{n}{}^{2-}↔2S_{n/2}^{•-}{}_{}$$

Together, these reactions provide a capacity of 419 mAh g^−1^. In the third step, the S_4_^2−^ ions obtain six electrons to generate S^2−^ ions at 2 V, producing 1256 mAh g^−1^. This process involves three separate transformation steps:

(50) $$3S_{4}{}^{2-}+2e^{-}\to 4S_{3}{}^{2-}$$

(51) $$2S_{3}{}^{2-}+2e^{-}\to 3S_{2}{}^{2-}$$

(52) $$S_{2}{}^{2-}+2e^{-}\to 2S^{2-}$$

At the end of the reduction process, various shorter polysulfide compounds, such as S_3_^2−^, S_2_^2−^, and S^2−^, can be detected in the final products. Additionally, advanced in situ X‐ray absorption near edge structure (XANES) spectroscopy was applied to analyze the mechanisms of redox behavior at sulfur electrodes.^240^ This work verified the existence of the soluble intermediates S_4_^2−^ and S_6_^2−^ despite their very short period. Subsequently, Lowe and co‐workers^241^ proved the involvement of some key species in Li–S battery operation, especially radical anions S_3_^•−^ and S_4_^•−^, which appeared at nearly 2.1–1.6 V. They also proposed that elemental sulfur is initially reduced to S_8_^−^ rather than S_8_^2−^. Therefore, there is no definite conclusion on the types of polysulfide involved. However, we have determined that the chemical equilibria of these polysulfides (shown in Figure 21c) lead the reaction to approach chemical reversibility under appropriate conditions. Similar research methods and conclusions can be partly applied to other sulfur‐like species such as in Li–Se batteries.^242^ According to the results of in situ (XRD) and X‐ray absorption spectroscopy (XAS), lithium polyselenides exist in Li–Se batteries when ether‐based solvents are used in the electrolyte.^243^ However, an absence of lithium polyselenides was reported for carbonate‐based electrolyte, indicating that Se was directly reduced into Li_2_Se, corresponding to one plateau during the discharge process. Meanwhile, the discharge capacity was limited to about 400 mAh g^−1^ in the voltage window of 0.8–3.5 V.

Although Li–S batteries have many advantages such as high energy density, low cost, abundant material reserves and environmental friendliness, there are still many problems plaguing their development. The sulfur cathode suffers from low electrochemical utilization and poor cycle life owing to the insulating nature of S and the Li_2_S discharge product, and the shuttling of lithium polysulfide species.^2^, ^244^ Enormous progress has been achieved in terms of capacity and static life during the past few years to overcome these issues, by employing sulfur‐carbon or sulfur‐conductive polymer nano‐composites or carbon‐coated separators.^2^, ^245^ These strategies not only restrain the “shuttle effect”, but also improve the electrical conductivity of the cathode. Additionally, biomass materials have been used to create sulfur based composite electrodes.^246^ The CV curves of sulfur/hardwood charcoal electroded exhibit high repeatability during charging/discharging. Further, because of the solubility of polysulphides is directly determined by the donor ability of the anionic structure, weaker donor ability is conducive to inhibiting dissolution.^247^ Developing novel electrolytes with low sulfur solubility as alternatives to conventional ether‐based electrolytes is a good problem‐solving approach. Solid electrolytes are also a good choice, because they not only restrain the dissolution of polysulfides, but also protect the metallic lithium electrode.^248^

## 7 Conclusions and Outlook

This brief overview of a series of secondary battery systems and related electrode materials that undergo multi‐electron mechanisms highlights the impressive developments achieved in multi‐electron reactions in recent years. Multi‐electron mechanisms provide a novel horizon for developing future batteries that will meet the demands for high energy density. In view of the requirements of EES, materials and advanced batteries based on multi‐electron concepts show promise for great breakthroughs in high energy density and high power density. There is still ample room left for improvement of multi‐electron systems. Future optimization of electrodes and intensive study of structural changes during electrochemical reactions will lead to further enhancement of the electrochemical performance of these systems. Certainly, the proposed solutions for the technical challenges related to the practical issues of various electrodes are far from mature and still require significant improvements and innovations from fundamental research to large‐scale applications.

Different battery chemistries present their own technical challenges. Commercial LIBs cannot seem to compete with the state‐of‐the‐art systems currently under development. An advanced Li‐ion battery using Li metal anodes and advanced cathode materials might give a practical energy density of ≈300 Wh kg^–1^, if a solution for the problems related to metallic lithium and the structural changes in the cathodes caused by multiple lithium intercalation is achieved. Anodes based on multi‐electron reactions such as metal oxides and alloys are superior substitutes for alkali metals for the moment. Cell configuration and detailed mechanism investigations are necessary strategies for current research. The Materials Genome Initiative has provided a new approach to the screening of materials with multiple electronic systems in the secondary battery field. Additionally, organic rechargeable batteries with flexible structure have a large migrating space for cations with large ionic radius such as Na^+^, Mg^2+^, and Al^3+^, providing further opportunities for researchers.

Other potential battery systems beyond secondary batteries based on lithium and sodium such as two electron Mg‐ion batteries, Li–S, and metal–air systems are also being considered as promising energy storage technologies. For Mg‐ion batteries, stable electrolytes and high potential reversible Mg intercalation compounds must be urgently found. Further, current research is based on wide variety of experimental conditions. Fundamental research on the intercalation mechanism of Mg and exact intercalation composition is important, because divalent Mg presents particular advantages. Research on Li–S batteries, an example of anions taking part in multi‐electron redox reactions, have entered an advanced practical stage. Two challenges still existing for these systems include the insulating nature of sulfur and the high solubility of the polysulfides produced during the multi‐transfer process. Therefore, how a balance between high capacity and cycling stability may be obtained is an important question. Limiting partial irreversible reaction as well as retaining reversible reaction may be a good strategy. Lastly, metal–air batteries belong to the multi‐electron system family in which anions also take part in the reaction, and possess advantages such as high energy density and low cost. The electrolytes involved can be divided into aqueous and non‐aqueous; aqueous systems have a long history and can be regarded as large‐scale storage technology, but exhibit relatively low energy density compared with that of non‐aqueous batteries. The principles underlying the multi‐electron mechanism involved in non‐aqueous Li–O_2_ batteries have been briefly reviewed in this paper. Fundamental studies of the oxygen reaction mechanisms in different electrolytes are important for developing highly efficient, high energy density batteries, especially in non‐aqueous systems.

Undoubtedly, multi‐electron reactions pave the way for improving the energy densities of sustainable secondary batteries. The intensive research carried out on this subject in recent years has broadened our horizons and understanding of the multi‐electron concept. Many different strategies have been applied in attempts to break the energy density barriers that exist in current battery technologies. Practical improvements in energy density are far from theoretical exceptions, even in laboratory studies. On the one hand, voltage limitation and electrolyte instability are factors that hinder multi‐electron redox reactions. On the other hand, some electrode materials can reversibly transfer more than one electron, although the resulting increased capacities are compromised by the heavy mole weight of the electrode material. Multi‐electron materials with two‐dimensional structure are research hotspots in recent years. Both of phosphor materials (red P and black P), chalcogenides (MoS_2_, SnS_2_, TiS_2_ and so on), graphene series materials, alloy materials (SnGe, SiGe and so on) etc. can be used as superior electrode for LIBs with high energy density since they contain the most of multi‐electron elements in our summary (Figure 1). These elements can provide a variable valence to achieve a multi electron transfer process. In addition, they exhibit stable architecture for monovalent/multivalent cations storage. Owing to the complementary effect of 2D structure and multi‐electron reaction, these novel electrode materials deserve to be sustained attention. Thus, the further development of multi‐electron reaction based systems must rely on lighter elements, as shown in Figure 2b, combined with the rational design of electrode architectures.
